# SiNG-PCRseq: Accurate inter-sequence quantification achieved by spiking-in a neighbor genome for competitive PCR amplicon sequencing

**DOI:** 10.1038/srep11879

**Published:** 2015-07-06

**Authors:** Soo A. Oh, Inchul Yang, Yoonsoo Hahn, Yong-Kook Kang, Sun-Ku Chung, Sangkyun Jeong

**Affiliations:** 1 Medical Research Division, Korea Institute of Oriental Medicine (KIOM), Daejeon 305-811, Korea; 2 Center for Bioanalysis, Korea Research Institute of Standards and Science (KRISS), Daejeon 305-340, Korea; 3 Department of Life Science, Chung-Ang University, Seoul 156-756, Korea; 4 Development and Differentiation Research Center, Korea Research Institute of Bioscience and Biotechnology (KRIBB), Daejeon 305-806, Korea

## Abstract

Despite the recent technological advances in DNA quantitation by sequencing, accurate delineation of the quantitative relationship among different DNA sequences is yet to be elaborated due to difficulties in correcting the sequence-specific quantitation biases. We here developed a novel DNA quantitation method via spiking-in a neighbor genome for competitive PCR amplicon sequencing (SiNG-PCRseq). This method utilizes genome-wide chemically equivalent but easily discriminable homologous sequences with a known copy arrangement in the neighbor genome. By comparing the amounts of selected human DNA sequences simultaneously to those of matched sequences in the orangutan genome, we could accurately draw the quantitative relationships for those sequences in the human genome (root-mean-square deviations <0.05). Technical replications of cDNA quantitation performed using different reagents at different time points also resulted in excellent correlations (*R*^*2*^ > 0.95). The cDNA quantitation using SiNG-PCRseq was highly concordant with the RNA-seq-derived version in inter-sample comparisons (*R*^*2*^ = 0.88), but relatively discordant in inter-sequence quantitation (*R*^*2*^ < 0.44), indicating considerable level of sequence-dependent quantitative biases in RNA-seq. Considering the measurement structure explicitly relating the amount of different sequences within a sample, SiNG-PCRseq will facilitate sharing and comparing the quantitation data generated under different spatio-temporal settings.

Recent advances in sequencing technology allow transcriptome-wide sequencing (RNA-seq) to generate information on abundance and variation at the level of a single nucleotide^1^,^2^,^3^,^4^,^5^. While this approach is highly reproducible and effective for estimating inter-sample differences^6^, it is yet to be used to accurately represent the relative quantities of different transcripts. Various procedural biases including random hexamer priming and flow cell attachment lead to incorrect inter-sequence quantities, resulting in a distorted transcriptomic landscape^7^,^8^,^9^. To negate such biases, adjustments in standard RNA-seq have implemented statistical and/or in-experiment designs such as incorporating standard RNA molecules or uniquely labeling each molecule for identification^7^,^8^,^9^,^10^,^11^,^12^,^13^. Nonetheless, more work is still necessary to determine whether these biases are completely corrected.

Besides transcriptome-wide analysis, a large part of biological and clinical research requires approaches that target for a selected group of transcripts. These approaches, in general, construct sequence libraries with target sequences captured using a tiling array^14^ or soluble probes^15^, or with competitive PCR amplicons^16^. In terms of inter-sequence quantitative representation, capture approaches suffer procedural biases similar to those of RNA-seq. Competitive PCR approach provides a means to account for the procedural biases by normalizing target quantities with known quantities of corresponding competitor templates. Because competitor templates have undergone the same biochemical events, they are assumed to have the same biases as their targets^16^. However, a critical limitation of this procedure is that different sets of competitor templates need to be manufactured, potentially by different manufacturers and under precise conditions to maintain the quantitative integrity.

The ultimate goal of nucleic acid quantitation is to estimate the number of molecules of every kind in a sample. This measurement is most effective for and applicable to samples that represent a quantitatively defined entity, such as a whole, single blastomere. For a sample that is a representative of a population, such as most biological samples are, it is more practical and useful to determine the relative quantities of the components in the sample. This enables one to define the quantity of a sequence as the relative value to the internal standard that represents the quantity of a standard sequence or group of sequences in the sample. If two or more independent measurements for different sets of partially overlapping components are acquired for the same sample, the measurement facilitates the quantitative comparison between the sets using the shared components.

We previously reported a method that determines the relative quantities of different sequences using a quantitative competitive PCR strategy^17^. In this approach, competitor templates were arranged in a plasmid to form a competitor array so that their relative quantities in the plasmid are explicitly known, thus providing definitive quantitative relationship of the competitor templates. In combination with melting analysis for allele quantitation^18^, this quantitation approach was proven to be highly accurate and precise in quantitating small numbers of sequences. Here, we further extended this concept to develop the spiking-in neighbor genome-coupled competitive PCR amplicon sequencing (SiNG-PCRseq for short). SiNG-PCRseq employs the genomes of species that are evolutionary neighbors (neighbor genome) as competitor templates, and achieves high-throughput using next generation sequencing (NGS) to quantitate competing sequences in amplicons. Since the neighbor genome contains a repertoire of competitor templates with known copy number arrangement for a wide range of target transcripts, this approach meets the demand for targeted inter-sequence quantitative RNA-sequencing for any category of sequences.

## Results

### Experimental structure of SiNG-PCRseq for accurate inter-sequence quantitation

**Figure 1:**
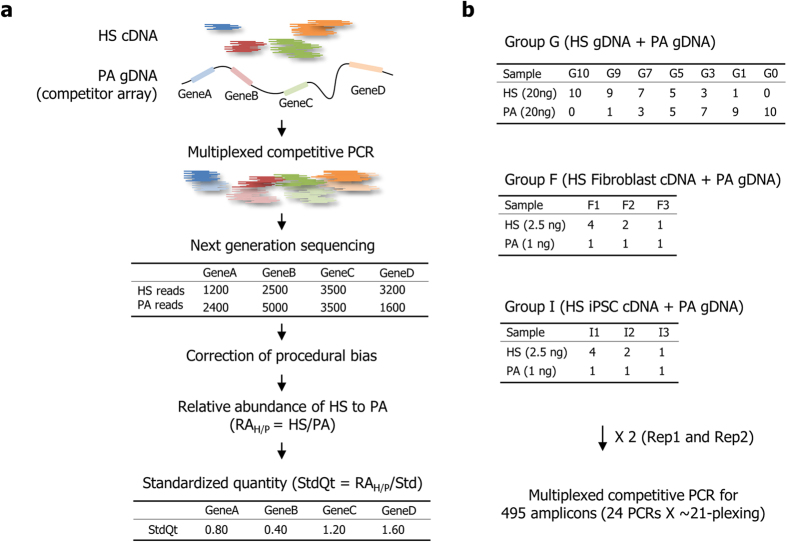
Quantification scheme and experiment design. (**a**). Quantification of human cDNA (HS cDNA) for four example genes (GeneA-GeneD) to represent their relative quantities to a standard value is step-by-step illustrated with arbitrary read numbers. Multiplexed PCR amplicons generated after spiking-in of the orangutan genome (PA gDNA) to the HS cDNA were sequenced to distinguish and quantitate the sequences according to their genic and species origins. After bias correction, the relative abundance of human sequence to corresponding orangutan sequence (RA_H/P_) was determined. RA_H/P_ was next processed to represent it as a standardized quantity (StdQt), which is a relative RA_H/P_ to a standard value such as the average of RA_H/P_s. (**b**). An experimental set was composed to determine the standard quantities of a subset of sequences in three sample groups for analyzing human gDNA (Group G), human fibroblast cDNA (Group F) and human iPSC cDNA (Group I). Each group includes a series of samples with the indicated proportion of spike-in orangutan gDNA. The quantity of cDNA denotes a corresponding RNA quantity for cDNA conversion. Two experimental sets (Rep1 and Rep2) were separately made with different Taq polymerases to form a technical replicate.

Figure. 1a illustrates the systematic procedure of SiNG-PCRseq to represent the quantities of a group of selected human cDNA sequences to standardized values with a reflection of their relative quantities. A set of experiments was composed with three sample groups using the orangutan genomic DNA (gDNA) as a spike-in competitor array to test our method for the quantitation of human sequences in gDNA (Group G) and cDNA samples (Group F and I) (Fig. 1b). Since the relative quantities of most transcriptomic sequences in human gDNA are explicitly known, analysis of gDNA sample allows us to determine the accuracy of our method. This set of experiments was repeated from the spike-in step for gDNA samples and from cDNA preparation for cDNA samples with an alternation of two different Taq polymerases to form Rep1 and Rep2 experimental sets as a technical replication. This configuration also enabled us to test the reliability and consistency of measurements between different experimental conditions and timing (such as occurs between different laboratories).

By aligning the human reference mRNA sequences for 263 genes to orangutan genome sequence, we designed, in total, 495 primer pairs to generate the amplicons, with an average size of 67 base pairs (SD = 10.4), carrying mostly single nucleotide inter-species variations (ISVs, for more information, see Supplementary table 1). The amplicons generated by 24 rounds of ~21-plexing PCR for each spiked-in sample were pooled and processed for sequencing library construction, during which the integrity and inter-sample consistency of the intermediary amplicon DNAs were verified by gel electrophoresis (Supplementary figure 1). Analysis of amplicon sequences that were generated with human (G0) or orangutan gDNA only (G10) identified 425 ISV-containing amplicons mapped onto 248 genes. The remaining 70 sequences were either not amplified at all (n = 4) or deficient for ISV (n = 66). The species-specific sequences were then used to construct a reference sequence library to which the amplicon sequences of other samples were aligned. The number of sequences aligned with 100% identity to the library sequences ranged between 1.4–2.9 and 1.2–1.9 million reads per sample in Rep1 and Rep2 sets, respectively. This figure accounts for approximately 61% of total sequence reads for the corresponding samples (Supplementary figure 2). Read depths for each amplicon (the sum of both the target and competitor sequences for the respective amplicons) in different samples were highly correlated in the within-set comparison, while inter-set comparison showed weaker correlations (Supplementary figure 3), demonstrating a consistent performance of each polymerase across the samples but different amplification characteristics between two polymerases.

### Application of SiNG-PCRseq for gDNA samples demonstrates high measurement accuracy

Using the human gDNA samples with varying fractions of orangutan gDNA spike-in (Fig. 1b), we determined the relative abundances of subjected sequences in the genome. After sequence reads alignment, amplicons achieving an average of less than 200 reads per sample and more than 5% impurity in G0 and G10 samples were abandoned, yielding 367 (86%) and 299 (70%) amplicons for subsequent assessment in the Rep1 and Rep2 sets, respectively.

**Figure 2:**
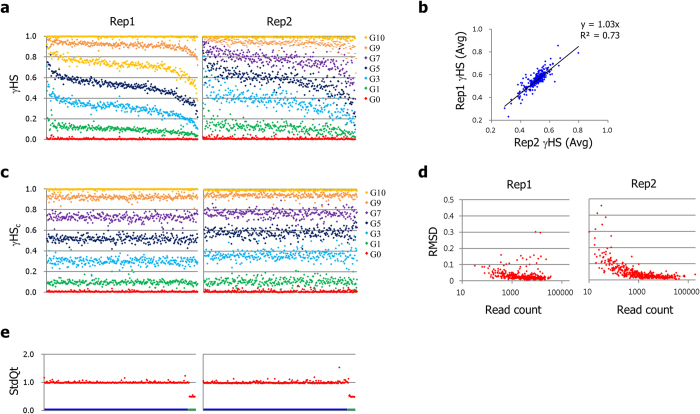
Determination of standard quantities of subjected sequences in human gDNA. (**a**). Human fractions (γHSs) in the amplicons of gDNA samples were determined using sequence analysis and arranged in the plot such that amplicons with higher averaged γHS in Rep1 set are placed to the left. (**b**). The averaged γHS values of the amplicons obtained from two experimental sets are plotted in a scatter diagram to reveal non-random procedural biases. (**c**). Bias corrected human fractions (γHS_c_s) are plotted with the same amplicon arrangement as in panel (**a**). (**d**). Root mean square deviation (RMSD) of each amplicon’s γHS_c_s from the expected human fractions in the samples plotted in a scatter diagram with the amplicon’s read counts. (**e**). Averaged standard quantities of all amplicons as measured from five gDNA samples plotted with an arrangement such that autosomal sequences come before X-linked ones as indicated with colored X-axis (blue for autosomal, green for X-linked sequences).

The fractions of human sequences (γHSs) in individual amplicons were determined based on the read alignment data and are plotted in Fig. 2a. Plot patterns that are uneven within each sample but synchronized across samples demonstrate directional quantity biases toward either their human or orangutan variant in a large part of the amplicons. The average γHS for each amplicon in all samples was highly correlated between two experimental sets (Fig. 2b). The extent and preference of these biases suggests that their cause is intrinsic to the variant structure rather than randomly occurring. Multiple rounds of reactions and purifications in our experimental procedures may cause such biases by enriching a competing sequence more favorably. Indeed, the variants that carry the nucleotides mediating strong hydrogen bonds (G and C) appeared to be more represented than those with weak hydrogen bonds (A and T) in our procedures (p = 6.8E-11, Mann-Whitney test) (Supplementary figure 4).

In our approach, we assumed that the total biases toward human or its competitor sequence would occur to a similar extent when the biases in the sample were summed. This assumption leads to the conclusion that the average γHS of all amplicons in each sample represents a close estimation of the fraction of human gDNA in that sample. Because one can easily calculate the extents of biases from any amplicons in each sample, we therefore corrected γHS values of all amplicons in a sample by evenly referring the extents of their biases in other samples (see method for more detail). As shown in Fig. 2c, the evenly arranged patterns of bias-corrected γHSs (γHS_c_s) in all samples emphasize efficient bias correction. The coefficients of variation (CVs) of γHS_c_s for each sample were in the range of 0.1–0.2. The extent to which the γHS_c_ values deviate from expected values were highly affected by the read depth in the Rep2 set but only marginally in the Rep1 set (Fig. 2d), again revealing different catalytic differences between polymerases.

We next obtained the standardized quantities for the gDNA sequences in the sample by applying the γHS_c_s to a two-step formula. Each γHS_c_ were first converted to the relative abundance (RA_H/P_) of the human sequence to the copy representation of its competitor sequence in the orangutan genome so that they reflect the copy number differences between autosomal and X-linked sequences in male orangutan genome. The RA_H/P_ quantities were then standardized to represent quantities relative to the average RA_H/P_ values of all autosomal sequences in each sample. Because the quantities as such obtained for all amplicon sequences become comparable on the same quantitative scale, we here used the term ‘standardize’ rather than ‘normalize’. The root mean square deviations from expected values (RMSDs) of standardized quantities (StdQts) ranged from 0.15–0.26 and 0.19–0.39 for Rep1 and Rep2 sets, respectively. Since StdQt values are taken over five independent samples with a varying composition of spiked-in gDNA, we averaged the StdQt values of each amplicon in all samples (Fig. 2e). This resulted in RMSDs of 0.03 and 0.05 for Rep1 and Rep2 sets, respectively, demonstrating that we achieved high accuracy over repeated measurements.

### SiNG-PCRseq is a consistent and reproducible method in cDNA quantitation

The analysis of human cDNAs with spike-in orangutan gDNA was performed to obtain their quantitative relationships in each of two human cell lines, a fibroblast (Fib) and an induced pluripotent stem cell line (iPSC) (Fig. 1b). Three cDNA samples were prepared with varying compositions of spike-in gDNA for each cell line. Procedural biases in γHS values were corrected by referring the extents of biases that the same amplicons in the gDNA samples (G1–G9) exhibited in the corresponding replication set. Amplicons with read counts below 200 and those devoid of corresponding gDNA data for bias correction were removed from the analysis.

**Figure 3:**
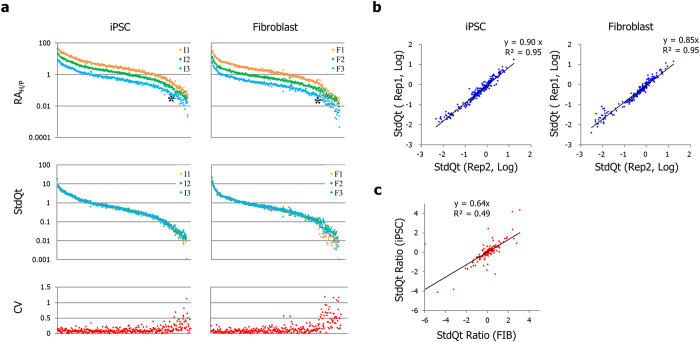
Determination of standard quantities in cDNA samples. (**a**). The quantity of each human sequence relative to the spike-in genome (RA_H/P_) with an adjustment of copy number imbalance of X-linked sequences, the standardized quantity of each sequence in each cDNA sample (StdQt) and the CV of three StdQt measures for each amplicon are separately plotted for two cell lines such that amplicons with higher averaged StdQts are placed to the left. The point that CV values are badly arisen is indicated by asterisks. (**b**). Log-transformed StdQts of human sequences in cDNA samples as determined from two experimental sets (Rep1 and Rep2) plotted in a scatter diagram for each cell line. (**c**). The ratio of standard quantities of two sequence segments in a single gene were obtained from two cell lines and visualized as a scatter plot.

The RA_H/P_ values for the Rep1 obtained thus far displayed averages of 5.3, 2.5, and 1.0 for each sample of iPSC and 3.0, 1.1 and 0.5 for each sample of Fib, which well reflects the varying composition of spike-in gDNA in different samples. Each of these average values was then used as the standard for determining the StdQt values for the corresponding sample (Fig. 3a), which displays a good agreement of StdQts obtained from three spike-in samples over a wide quantity range (RA_H/P_ > 0.05). The CVs of StdQts from three samples in this quantity range were kept relatively low (less than 0.5), with averages of 0.13 and 0.16 for iPSC and Fib, respectively. The copy number of an autosomal unique sequence in 1 ng of spike-in gDNA is approximately 300, which corresponds to an estimate of 15 copies of corresponding human sequences with an RA_H/P_ of 0.05. Therefore, sampling variance due to small numbers of templates in the PCR can be a demonstration of high measurement variance in the low RA_H/P_ range. When we rearranged the data according to the order of read depth, we observed a trend indicative of a low read count associated with high measurement variance, but the association was not as tight as that seen in Fig. 3 (Supplementary figure 5). One potential reason for this weak association might be because of drop-out of the amplicons that did not reach a read count of 200 in the analysis. This data provides an insight into a dynamic measurement range for directly comparing the abundances of different sequences within the RA_H/P_ of 20–0.05; these values correspond to a 400 fold difference in relative abundance. This measurement window can be arranged to meet one’s need simply by adjusting the ratios of spike-in gDNAs in cDNA samples.

The Rep2 set had inferior quantitation performance compared to the Rep1 set, as many of the amplicon sequences in the Rep2 set were excluded from the analysis due to low read depths, resulting in 240 (56%) retrieved sequences as compared to 365 (86%) in the Rep1 set. Nonetheless, the correlation (*R*^*2*^) of log-transformed StdQts obtained for the 240 sequences common to both experimental sets were 0.95 for both iPSC and Fib, highlighting the highly reproducible nature of our method even when performed under different reaction conditions at different time points (Fig. 3b).

One of our concerns in cDNA quantitation was that the quantity of a sequence so far obtained might be insufficient for representing the transcript abundance of the corresponding gene due to the segmental quantitative variations in the cDNA of the gene. We therefore wanted to address the extent of the consistency in the quantitation of different cDNA positions in the same gene. Of the 230 genes that were initially designed to assess the cDNA abundances at their multiple loci, we were able to quantitate 106 in the Rep1 set (105 at two loci; 1 at three loci). The CVs of multiple loci of individual genes were averaged to 0.32 and 0.28 for iPSC and Fib, respectively, indicating relatively consistent quantitative representation of multiple loci for the same transcript. The plot of the StdQt ratios between two measurement points of single genes in iPSC against those in Fib revealed a relatively high correlation (*R*^*2*^ = 0.49), which suggests common factors in the two cell lines that account for the differences in quantitative representation between multiple cDNA segments observed for some genes (Fig. 3c). It appears to reflect the differential abundances in the cDNA pool of those sequences as likely occurred by differential reverse transcription efficiencies on those loci and/or the existence of different isoforms in the transcript that differentiate the local abundance. In supporting the latter case, 11 out of 28 genes (39%) with CVs more than 0.47 (a value representing more than two-fold difference between two loci) were found or predicted to express at least one isoform that lacks one of the two loci, whereas only 17% of the remaining genes with relatively consistent representation by multiple loci show such isoform profiles. This finding awaits further validation.

### Differential performance in inter-sequence quantitation between the SiNG-PCRseq and RNA-seq

**Figure 4:**
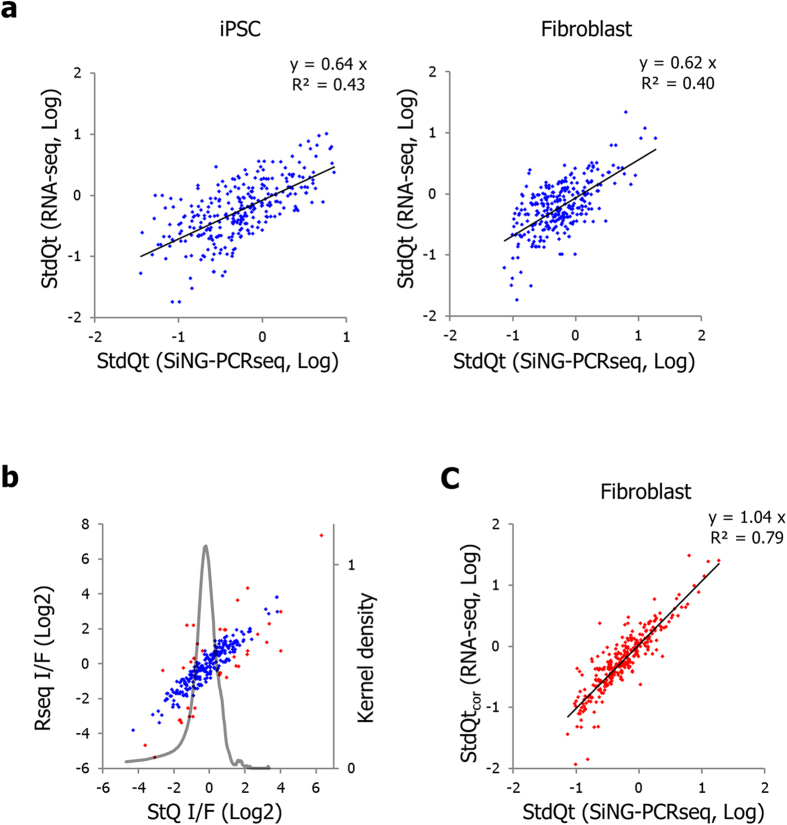
Comparison of SiNG-PCRseq with RNA-seq. (**a**). The log-transformed standardized quantities obtained from two different quantitation approaches, SiNG-PCRseq and RNA-seq, plotted as scatter diagrams for two cell line samples. (**b**). The fold differences of cDNA quantities in two cell lines (I/F) determined for two different quantitation approaches and scatter plotted. Genes showing more than two fold differences in I/F are indicated as red. Kernel density (black solid line, right axis) plotted for fold differences in I/F between two measurement approaches. (**c**). Standard quantities derived from the RNA-seq method were corrected for systematic bias (StdQt_cor_) and are presented as a scatter plot against the standard quantities from SiNG-PCRseq.

Although the quantitative landscape among different sequences determined with RNA-Seq is highly reproducible, these experiments have not been thoroughly validated for the fidelity of inter-sequence relative quantities due to lack of available technology. To address this issue, we compared the RNA-seq data with our measurements. The RNA-seq data for the same cells used in this study was obtained in a companion study that assesses the effect of chimpanzee RNA spike-in on the normalization of RNA-seq data of human samples (Yu, H. *et al.*, manuscript submitted). Read counts for the reference mRNAs corresponding to the amplicons in this study were normalized with their length to yield RPKM values. For this comparison, we selected items that fall into a set of specific criteria in order to include the qualified measurements only; those with an RA_H/P_ of more than 0.05 in all samples in SiNG-PCRseq and those with read counts more than 40 in RNA-seq. RA_H/P_ and RPKM values were standardized using the averages of those respective values of the selected items. The gene-by-gene correlations (*R*^*2*^) of log-transformed StdQt values between two approaches were 0.43 and 0.40 for iPSC and Fib, respectively (Fig. 4a), indicating considerable differences in measurements of inter-sequence quantitation between two methods. The fold difference of standardized quantities between two cells (I/F) are highly correlated between two measurements (*R*^*2*^ = 0.88) (Fig. 4b). Of the 281 items, only 38 (13%) exhibited more than two fold differences between the I/F values of two measurements. The kernel density plot also demonstrates good agreement between the two approaches in inter-sample quantitative comparison. To examine whether the differences in StdQts between the two methods were due to a systematic bias, we calculated the gene-by-gene biases between the StdQts for iPSC and applied them to correct the biases in Fib. As shown in Fig. 4c, the bias-corrected StdQts of RNA-seq-derived data were highly correlated with those of SiNG-PCRseq-derived data. These results suggest the equivalent performance of the two methods in acquiring inter-sample quantitative differences of the same cDNAs, and the systemic bias between the two methods that accounts for the discordant intermolecular quantitative landscapes.

## Discussion

In this work, we presented SiNG-PCRseq with ample experimental evidence that demonstrates its reliability and reproducibility in accurately quantitating DNA sequences with an emphasis on the inter-sequence relative quantitation. Our method takes advantage of a neighbor genome by using it as a transcriptome-wide repertoire of sequences homologous to the target. Currently more than one hundred eukaryotic annotated genome sequences are available, allowing the application of this method for samples from a variety of species, ranging from worms to humans (http://www.ncbi.nlm.nih.gov/genome/annotation_euk/all/). As the outcomes of SiNG-PCRseq are the quantities of target sequences relative to the quantitation standard, one can easily compare these results even between different species if the standards used in those species are biologically or analytically equivalent to one another^16^,^17^. The quantitation standard can be adapted with the RA_H/P_ of a single sequence or a combination of sequences in a biological relationship. For example, a gene set comprising transcriptional machinery can be used as a standard to express each measurement as the transcript level in relation to cellular transcriptional activity. Thus, the merit of this method is that the quantities measured from various biological samples are comparable by the common standard, no matter when or where they are obtained. In addition, standardization of the quantitative landscape is flexible and can be performed later by using shared sequences as quantitation standard in new samples that come into comparison, allowing every measurement for multi-time use availability.

One element that improves the reliability of our method is the algorithm that corrects the procedural biases. Multiple rounds of PCR amplifications might be a major cause leading to distortion of the original composition of competing sequences in the final end products. These biases did not occur randomly but appeared to be related to the variation structure of competing sequences. This type of bias can be corrected efficiently using one or multiple reference samples that the biases of a competing sequences and the fractions in those reference samples are known^18^,^19^. Here we took the average γHS of all amplicons in each gDNA sample as the human gDNA fraction of that sample for bias correction since we assumed that the extents of total biases toward human and orangutan sequences are equal. The validity of this assumption is supported by high correlations with regression slopes near 1 between the StdQt values obtained for cDNA samples with two independent measurements (Rep1 set and Rep2 set) where different reference values were applied for bias correction (Fig. 3b). This also adds convenience in the utility of our method as it eliminates the need of determining the fractions of ISV sequences in the reference samples for bias correction.

In the analysis of cDNAs, we were able to quantitate 106 genes for their cDNAs at multiple different loci. About half of them (44% of Fib and 48% of iPSC for Rep1 set) exhibited consistent quantitative representation at multiple loci (less than 20% difference between the loci), suggesting a uniformity of reverse transcription efficiency throughout the mRNAs of those genes. However, we have yet to validate whether the existence of multiple isoforms and/or the different reverse transcription efficiencies in different loci are responsible for larger differences in quantitation for rest of the genes. Our quantitation scheme could be applied to the delineation of quantitative relationships among isoforms of any single gene. Quantitative information on isoforms of a transcript could be obtained by comparison of a set of isoform-specific amplicons that have been properly calibrated with respect to inter-species nucleotide variations. However, this will require sufficient presence of isoform- or exon-specific nucleotide variations between the target and internal standard species. If a neighbor genome does not have sufficient occurrence of exon-specific sequence variations, synthetic standards with defined exon- or isoform-specific nucleotide variations could be alternatively used for this purpose^17^. Nonetheless, our method shows potential of addressing a critical concern in transcriptome analysis, i.e. the paucity of applicable means to analyze the quality of reversely transcribed cDNA in terms of uniformity in the representation throughout an entire transcript^4^.

Our study underwent a massive sequencing of a pool of 26 samples consisting of 495 amplicons encompassing 263 genes, yielding 75.4 million reads from single-end reading. Due to the stringent alignment and dropping out of sequences that do not distinguish species origin, sequences enrolled in the analysis were down to 46 million reads (61% of total reads). When we took into consideration of dropped amplicons due to lack of ISV, 71% of total reads are estimated to align to reference sequences with 100% identity. Based on data obtained with the Rep1 set, 6.6 million reads were sufficient for the analysis of 365 ISV loci in a sample with triplicate set up. This corresponds to 11 samples for equivalent loci or a single sample for 4,000 ISV loci when analyzed with a single NGS run that yields a sequencing throughput of 100 million single-end reads. In addition, our method would best meet the experimental need that focuses on small numbers of genes for comparison in a large group of samples. Thus, SiNG-PCRseq can flexibly accommodate the number of target sequences and samples in relatively wide ranges at one’s convenience.

An important parameter in this analysis is the quality of the multiplexed PCR with respect to the representation of the amplicons with sufficient number of reads. In the analysis of gDNA samples, amplicons in Rep1 set were read 5,187 times in average (SD = 5,643) with a maximum count (MAX) of 36,000 and 95% coverage (≥200 reads) in amplicon assessment. On the other hand, the Rep2 set performed worse than Rep1 set, with 4,626 read counts on average (SD = 12,811) with MAX of 194,000 and 77% coverage. In this regard, it is desirable to set the reaction condition or to use the enzyme that guarantees a better profile in acquiring the quantitative uniformity among the different amplicons.

The SiNG-PCRseq uses a neighbor genome as an array of competitor templates for competitive PCR and adapts NGS for quantitative analysis of multiple amplicon sequences. This analytical configuration was proven to be highly accurate and reproducible in the analysis of gDNA and cDNA sequences. Considering the capability to standardize the quantities of individual sequences using internal standard, the utility of SiNG-PCRseq could be substantiated for a range of biomedical applications by facilitating sharing and comparing the quantitation data generated different laboratories and times.

## Methods

### Cell culture and nucleic acid preparation

A lymphoblastoid cell line from a male orangutan and a human foreskin fibroblast cell line were purchased from Public Health England and System Biosciences, respectively. Cells were maintained in either RPMI1540 (lymphoblastoid cells) or DMEM (fibroblast cells) media containing 15% FBS. Human iPSC was derived from a human immortalized lymphoblastoid cell line by following the protocol reported previously^20^,^21^ under the approval of the Institutional Review Board committee of the Korea Institute of Oriental Medicine (I-1210/002/002-02) and maintained under mTesR1 conditional medium (Stemcell Technologies Inc.) with 0.5 mM sodium butylate (Sigma) and 25 μM SB431542 (Sigma). Genomic DNAs and total cellular RNAs were isolated using G-DEX^TM^ IIC (iNtRON Biotechnology, Korea) and TRIzol® Reagent (Life Technologies), respectively, following the manufacturer’s instructions. cDNA was prepared from 1μg of total RNA using iScript^TM^ cDNA Synthesis Kit (BioRad), as recommended by the manufacturer.

### PCR

Human reference mRNA sequences for 263 genes were retrieved from the NCBI Reference Sequence Database and individually aligned to their respective *Pongo abelli* (Sumatran orangutan) genome sequence (NCBI Pongo_pygmaeus_abelii-2.0.2 assembly) via BLAST to find homologous sequence stretches containing as few nucleotide ISVs as possible. The genes in this study were chosen based on our own research interests: the majority of the genes are related to epigenetic regulation mechanisms (Supplementary Table 3). Homologous sequence stretches were subjected to Primer3^22^ to design primer pairs that bind both human and orangutan sequences simultaneously in PCR amplicon while possessing the small numbers of ISV nucleotides in its amplicons to discriminate species origin. Primers were pooled into 24 groups of ~20 pairs in such a way that multiple primer pairs for a single transcript are separately assigned to different groups, to avoid the production of unintended amplicons. A multiplexed PCR reaction was carried out using each primer group and either of two Taq polymerases, Solg^TM^ h-Taq DNA polymerase (SolGent, Korea) or FastStart Taq polymerase (Roche) for the Rep1 set or Rep2 set, respectively, with the following conditions: 15 min of enzyme activation at 95 °C followed by 40 cycles of 95 °C for 20 s, 55 °C for 25 s, and 65 °C for 2 min.

### Sequencing

All amplicons generated with 24 rounds of multiplexed PCR were pooled together using equal volumes for each sample. Sequencing libraries were constructed by a set of reactions with intermittent purifications using Expin^TM^ PCR (GeneAll, Korea). The reactions include 5′-end phosphorylation, adaptor ligation and 2 further PCR amplifications to attach the sequence module enabling flow cell attachment, sequencing primer binding and barcoding. All PCR reactions were performed using the corresponding Taq polymerase for Rep1 set or Rep2 set. The 5′-ends of purified pooled amplicons (1 μg) were phosphorylated using T4 Polynucleotide Kinase (Promega) following the manufacturer’s recommendation. Ten microliter of Y-shaped adaptor molecule (15 μM, see Supplementary table 2 for oligonucleotides information) for the Illumina sequencing platform was added to the phosphorylated amplicon (6 ng) using T4 Ligase (Promega). A first PCR for sequence module attachment was carried out using the purified adapted amplicons (4 pg) and primers, MP1 and MP2, with a reaction condition, 15 min of enzyme activation at 95 °C followed by 20 cycles of 95 °C for 10 s, 65 °C for 30 s, and 72 °C for 2 min. A second PCR was performed using the purified first PCR amplicon (0.05 volume of the first PCR) and its primers (MP1 and one of IdxPs) to complete the sequence module attachment using the same reaction conditions of first PCR but with 10 thermal cycles. The barcoded sequencing libraries for all samples were pooled with equivalent amount and subjected to a multiple parallel sequence using Illumina HiSeq 2500 platform with a single-end 100 base pair reading.

### Sample-specific reference sequences

The initial human and orangutan reference sequences were obtained from the National Center for Biotechnology Information (NCBI) database. However, these sequences could not be readily exploited in this study due to possible sequence variations. To identify specific reference sequences for the human and orangutan cells used in this study, amplicon sequences derived from pure human (G10) or orangutan (G0) samples were analyzed. First, BLAST searches of amplicon reads of pure samples against the NCBI reference sequences were performed. The command line parameter was: “blastn -task blastn -db INIT_REF -outfmt 10 -evalue 1e-5,” where “INIT_REF” is a BLAST-searchable database containing the initial human and orangutan reference sequences obtained from the NCBI. BLAST outputs were parsed using a series of ad hoc Perl (version 5.18.2) scripts. Reads that matched a reference sequence with an aligned length of at least 75 bp or with a query coverage of at least 90% were collected. Then, the reads of each amplicon target were multiply aligned and a consensus sequence was constructed. At each aligned position, sequences exhibiting at least 25% of total reads were retained. Sequences that were monomorphic (sequences with heterozygous positions were discarded) and had high sequencing depth (greater than or equal to 100) were retained. Finally, human/orangutan sequence pairs that had sequence difference between the two species were collected as the sample-specific reference sequences: a final sample-specific reference sequence database containing 425 sequence pairs was constructed.

### Amplicon reads identification

Reads of amplicon-mix samples were identified by BLAST searches against the sample-specific reference database. The command line parameter was: “blastn -task blastn -db NEW_REF -outfmt 10 -evalue 1e-5 -perc_identity 100,” where “NEW_REF” is a BLAST-searchable sample-specific reference sequence database. BLAST outputs were parsed using a series of ad hoc Perl scripts. Reads that aligned a unique reference sequence with 100% sequence identity over a length of at least 75 bp or with a query coverage of at least 90% were assigned to the reference sequence. For each amplicon target, numbers of reads derived from the human mRNA and the orangutan genome were counted and their ratio was calculated (Supplementary table 4).

### Bias correction

Deviations in the fractional quantities of amplicons from original values in the sample were corrected as previously reported^18^ with a slight modification to utilize multi-point references. The above reference uses a single reference mixture with an equal contribution of two competing sequences, A and B. According to this reference, the bias corrected fraction of the sequence A, γA_c_, in a sample can be obtained using the following equation:
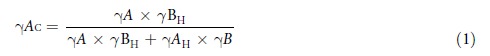
where γA and γB are the observed fractions of A and B in the sample and γA_H_, and γB_H_ are those in the equi-molar reference, respectively. We modified this equation for the reference sample with known but unequal fractions of A and B, pA and pB, and with observed fractions, γA_Ref_ and γB_Ref_, respectively, as below:
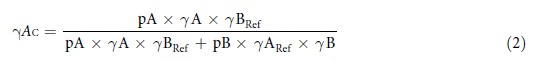
This equation was further extended to fit the situation where multiple reference samples are used for bias correction as below,
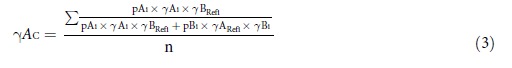


### Data analysis

Statistical analysis for pairwise Pearson correlation and linear regression was performed using Microsoft Excel 2010 (version 14.0.7140.5000). Non-Gaussian distributed variables such as StdQt values were log-transformed before subjecting the data to linear regression analysis. The non-parametric Mann-Whitney test was performed using the SPSS software package (ver. 22) for testing the difference of medians between the two γHS data sets, W (n = 161) and S (n = 165), representing the nucleotides for weak and strong hydrogen bonds, respectively.

## Additional Information

**How to cite this article**: Oh, S. A. *et al.* SiNG-PCRseq: Accurate inter-sequence quantification achieved by spiking-in a neighbor genome for competitive PCR amplicon sequencing. *Sci. Rep.* **5**, 11879; doi: 10.1038/srep11879 (2015).

## Supplementary Material

Supplementary Information
