# Supplementary material for: SiNG-PCRseq: Accurate inter-sequence quantification achieved by spiking-in a neighbor genome for competitive PCR amplicon sequencing
Source: Sci Rep. 2015 Jul 6;5:11879. doi: 10.1038/srep11879 (PMC4491706; doi:10.1038/srep11879)
Supplement: Supplementary Information [file srep11879-s1.pdf]

Supplementary Information

SiNG-PCRseq: Accurate inter-sequence quantification achieved by spiking-in a neighbor genome for competitive PCR amplicon sequencing

Soo A Oh, Inchul Yang, Yoonsoo Hahn, Yong-Kook Kang, Sun-Ku Chung and Sangkyun Jeong

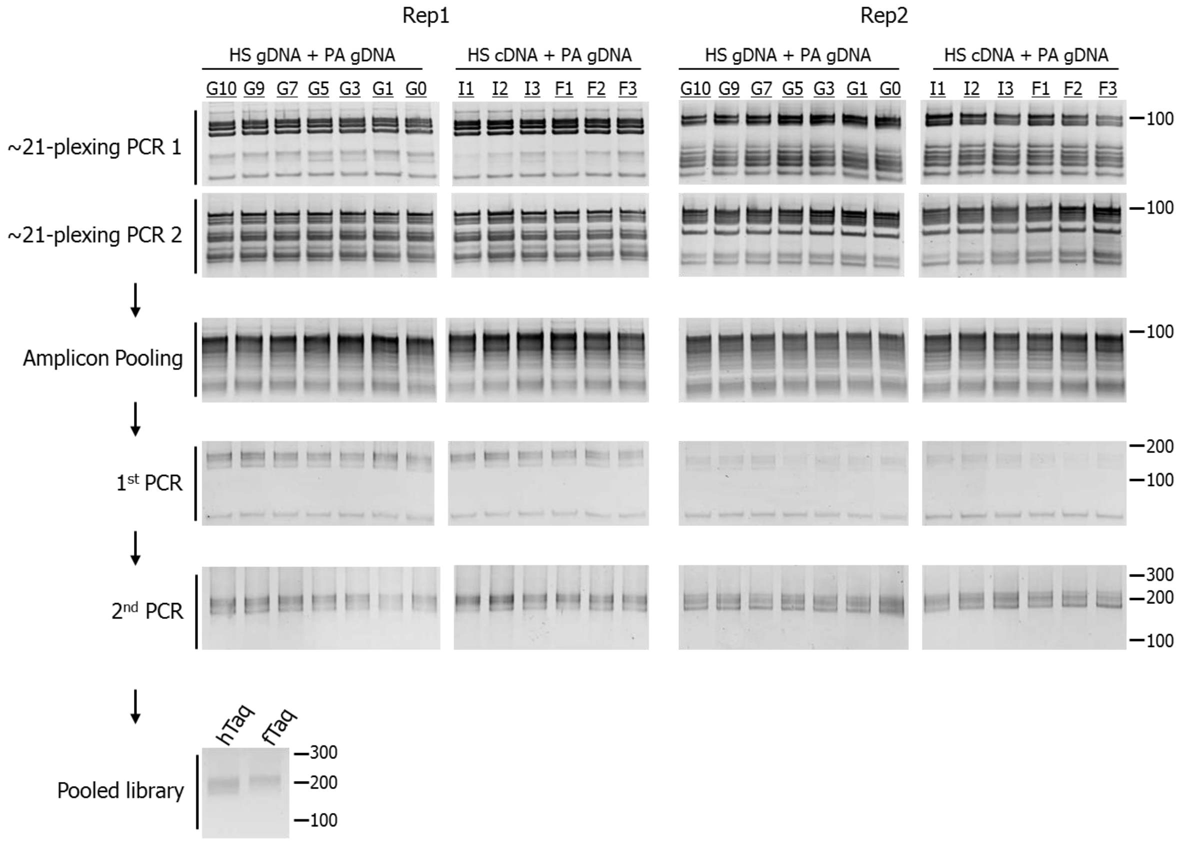

Supplementary Figure 1. Verification of the integrity of intermediary amplicon DNAs during sequencing library preparation

Amplicon DNA from two representative ~21-plexed PCR reactions, pools of all multiplexed PCR reactions, two consecutive PCR reactions for sequencing module attachments (first PCR and second PCR) and the library pools were all verified for their integrity and sample-to-sample consistency through electrophoresis on polyacrylamide gel (12%). Size markers are denoted on the right of gel images.

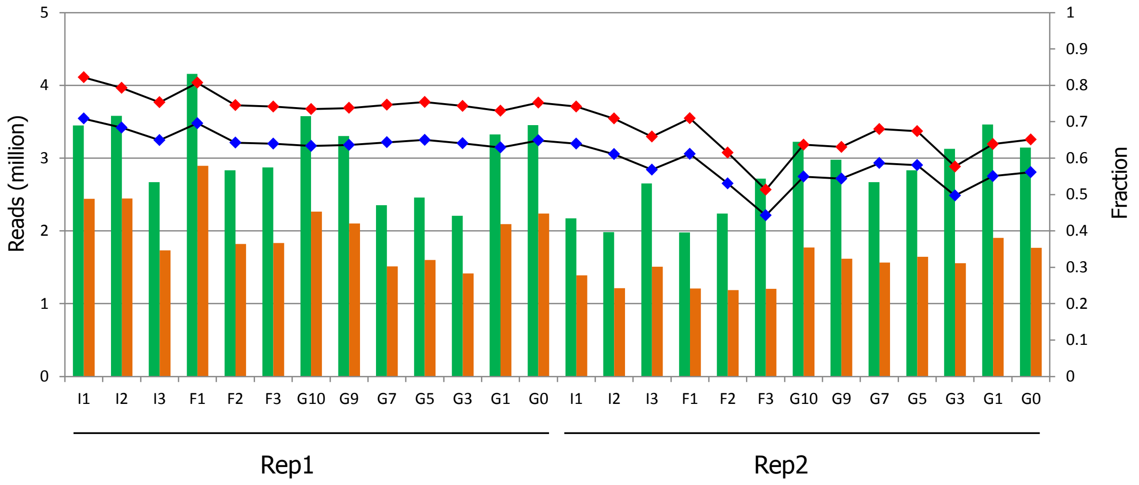

Supplementary Figure 2. Graphical representation of reads statistics

The sizes of the total reads and the informative reads for quantitation in each sample are represented with bars. The usage rates of sequenced reads in this study (blue diamond) and in an ideal case without abandoning sequences due to absent ISV (red diamond) were also represented.

		Rep1												
		G10	G9	G7	G5	G3	G1	G0	I1	I2	I3	F1	F2	F3
Rep1	G10	1.000	0.977	0.970	0.971	0.961	0.944	0.947	0.780	0.827	0.885	0.795	0.830	0.906
	G9		1.000	0.994	0.982	0.973	0.960	0.956	0.765	0.815	0.892	0.804	0.852	0.914
	G7			1.000	0.988	0.983	0.973	0.969	0.772	0.822	0.897	0.814	0.862	0.921
	G5				1.000	0.994	0.982	0.981	0.783	0.833	0.899	0.817	0.855	0.918
	G3					1.000	0.992	0.991	0.784	0.836	0.902	0.821	0.863	0.915
	G1						1.000	0.996	0.780	0.832	0.903	0.817	0.870	0.908
	G0							1.000	0.784	0.834	0.896	0.815	0.861	0.907
	I1								1.000	0.987	0.940	0.847	0.825	0.843
	I2									1.000	0.971	0.871	0.869	0.890
	I3										1.000	0.883	0.915	0.934
	F1											1.000	0.968	0.945
	F2												1.000	0.969
	F3													1.000

		Rep2												
		G10	G9	G7	G5	G3	G1	G0	I1	I2	I3	F1	F2	F3
Rep1	G10	0.623	0.601	0.604	0.586	0.543	0.551	0.558	0.504	0.533	0.560	0.514	0.520	0.549
	G9	0.649	0.636	0.645	0.623	0.572	0.581	0.590	0.518	0.557	0.595	0.545	0.555	0.582
	G7	0.661	0.652	0.661	0.640	0.589	0.598	0.607	0.538	0.579	0.617	0.569	0.578	0.604
	G5	0.632	0.622	0.635	0.616	0.569	0.578	0.587	0.522	0.559	0.594	0.543	0.552	0.578
	G3	0.631	0.622	0.636	0.617	0.571	0.582	0.592	0.537	0.573	0.608	0.559	0.567	0.586
	G1	0.622	0.612	0.626	0.608	0.562	0.576	0.588	0.541	0.577	0.613	0.564	0.571	0.583
	G0	0.609	0.598	0.613	0.596	0.552	0.567	0.578	0.528	0.560	0.593	0.552	0.557	0.573
	I1	0.476	0.456	0.462	0.447	0.411	0.423	0.435	0.614	0.618	0.599	0.543	0.522	0.510
	I2	0.526	0.507	0.513	0.497	0.457	0.468	0.481	0.638	0.649	0.640	0.587	0.571	0.561
	I3	0.577	0.564	0.572	0.552	0.505	0.516	0.529	0.636	0.664	0.676	0.610	0.605	0.600
	F1	0.540	0.531	0.540	0.521	0.479	0.489	0.504	0.581	0.595	0.597	0.682	0.656	0.629
	F2	0.592	0.584	0.592	0.566	0.515	0.524	0.543	0.608	0.635	0.653	0.714	0.706	0.677
	F3	0.616	0.607	0.614	0.594	0.546	0.554	0.566	0.586	0.611	0.629	0.669	0.662	0.665
Rep2	G10	1.000	0.994	0.983	0.975	0.956	0.954	0.955	0.825	0.853	0.883	0.823	0.856	0.910
	G9		1.000	0.994	0.987	0.968	0.965	0.963	0.817	0.850	0.886	0.827	0.861	0.918
	G7			1.000	0.995	0.976	0.973	0.970	0.800	0.833	0.873	0.817	0.847	0.906
	G5				1.000	0.992	0.990	0.986	0.810	0.832	0.862	0.817	0.840	0.899
	G3					1.000	0.998	0.993	0.812	0.820	0.838	0.806	0.820	0.878
	G1						1.000	0.996	0.821	0.825	0.840	0.811	0.822	0.877
	G0							1.000	0.833	0.838	0.850	0.823	0.836	0.879
	I1								1.000	0.986	0.946	0.908	0.899	0.878
	I2									1.000	0.984	0.909	0.916	0.903
	I3										1.000	0.893	0.917	0.919
	F1											1.000	0.989	0.957
	F2												1.000	0.977
	F3													1.000

1 0

Coefficient of correlation

Supplementary Figure 3. Pairwise comparison of Pearson correlation for the amplicon reads between samples

Pearson correlation coefficients between amplicon reads were obtained for every pair of samples and represented with numbers and colors.

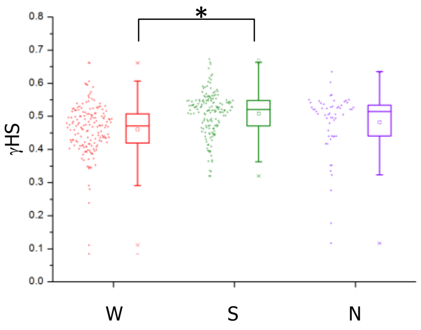

Supplementary Figure 4. Distribution of averaged γHSs according to the variation types

Human sequence variations were divided into three groups, W, S, and N, according to the strength of hydrogen bonds in relative to the orangutan variations to denote weaker, stronger, and neutral variations, respectively. A density plot and a quartile box plot of γHSs for each group were presented to visualize the relationship of variation type to the bias. *p = 6.8E-11 as determined by Mann-Whitney test.

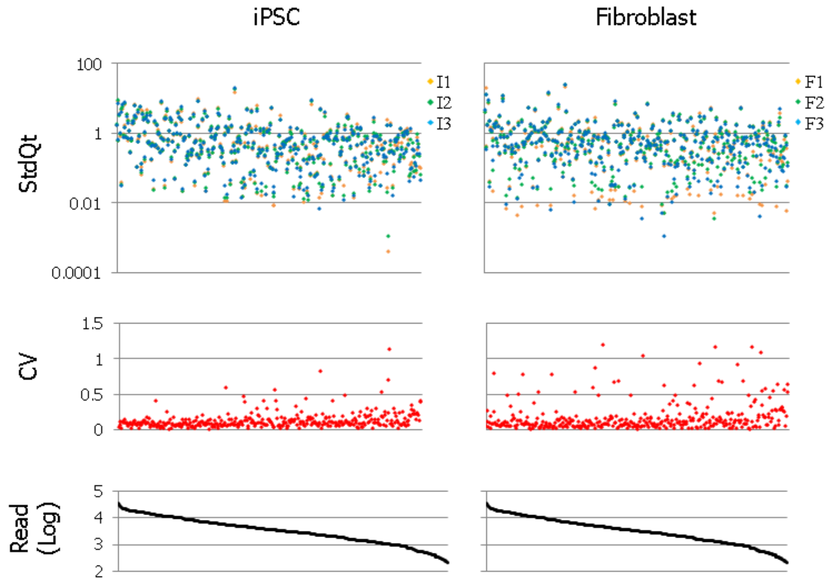

Supplementary Figure 5. Quantity data arrangement by amplicon read depth

Standardized quantities (Top, StdQt) and CVs (Middle, CV) for the amplicons were rearranged in a high-to-low read depth order (Bottom, Read).

Supplementary Table 1. Features of amplicon DNAs

Genes	Amplicons	Length		Variation			Amplicon per Gene			
		AVG	SD	Single	Double	Triple	1	2	3	4
263	425	67	10.3	382	36	7	32	228	1	1

Supplementary Table 2. Oligonucleotide information

Oligo Name	Sequence	Comment
Y-adaptor 1	5′-Pi-GATCGGAAGAGCACACGTCT-3′	5′-phospholyated (Pi)
Y-adaptor 2	5′-ACACTCTTTCCCTACACGACGCTCTTCCGATCT-3′	
MP1	5′-AATGATACGGCGACCACCGAGATCTACACTCTTTCCCTACACGACGCTCTTCCGATCT-3′	
MP2	5′-GTGACTGGAGTTCAGACGTGTGCTCTTCCGATCT-3′	
IdxPs	5′-CAAGCAGAAGACGGCATACGAGAT[******]GTGACTGGAGTTCAGACGTGTG-3′	[]: six-nucleotide barcode sequences

Supplementary Table 3

Gene	Amplicon	Human mRNA		Orangutan gDNA		Ori	Sequence
		Ref	Location	Ref	Location		
ACTL6A	ACTL6A-1	NM_178042.3	843—913	NC_012594.1	183222816-183222886	H	ATCCCCTCTTGCTGGAGACTTTATTACTATGCAGTGCAGAGAACTCTTCCAAGAAATGAATATTGAATTGG
						O	ATCCCCTCTTGCTGGAGACTTTATTACTATGCAATGCAGAGAACTCTTCCAAGAAATGAATATTGAATTGG
	ACTL6A-2	NM_178042.3	60—128	NW_002913225.1	4853-4921	H	CTCCGGGGTGTGTGGACGCCGCTTTGTTGCCTGAGGTGGGTGGCGGTGGAAGTTAAGGGAGTCAGGGGC
						O	CTCCGGGGTGTGTGGACGCCGCTTTGTTGCCTGAGGGGGGTGGCGGTGGAAGTTAAGGGAGTCAGGGGC
AEBP2	AEBP2-1	XM_006719031.1	3563—3629	NC_012603.1	20274389-20274455	H	TCCAGAATTTATGTATTGTTCAGCATCAAGCAAACTACAGCTCACAAGCATACCCATTTATATGTTG
						O	TCCAGAATTTATGTATTGTTCAGCATCAAGCAAACTGTAGCTCACAAGCATACCCATTTATATGTTG
ARF3	ARF3-3	XM_005268856.1	2372—2433	NC_012603.1	48595382-48595321	H	CAGGATATGCTAAAGGACGAAGATTTTCTATTGTTTCCAGGCCCTCAGACCATCCTTGCTGT
						O	CAGGATATGCTAAAGGACGAAGATTTTCTATTGTTTCCAGGTCCTCAGACCATCCTTGCTGT
ARF4	ARF4-4	NM_001660.3	1447—1498	NC_012594.1	88669573-88669624	H	TGGGGTTTGGATTTGATAGCCAATTAGTTCTGTGCTGGTTGCAAAGAATTGA
						O	TGGGGTTTGGATTTGATAGCTAATTAGTTCTCTGCTGGTTGCAAAGAATTGA
ARF5	ARF5-1	NM_001662.3	553—597	NC_012598.1	124647767-124647811	H	GTGAGCGAGCTGACTGACAAGCTGGGGCTACAGCACTTACGCAGC
						O	GTGAGCGAGCTGACTGACAAACTGGGGCTACAGCACTTACGCAGC
	ARF5-2	NM_001662.3	775—853	NC_012598.1	124648113-124648191	H	CCTTTCCTCCCACTTTTCCTCCCCCATAGCCACAGGCCTCTGCTCCTGCTCCTGCCTGCATGTTCTCTCTGTTGTTGGA
						O	CCTTTCCTCCCACTTTTCCTCCCCCACAGCCACAGGCCTCTGCTCCTGCTCCTGCCTGCATGTTCTCTCTGTTGTTGGA
ARID4B	ARID4B-1	XM_006711781.1	2366—2438	NC_012591.1	14374626-14374698	H	GCTTGTCCAAACCACCATTTCAGACAAATCCATCTCCTGAAATGGTATCCAAACTGGATCTCACTGATGCCAA
						O	GCTTGTCCAAACCACCATTTCAGACAAATCCATCTCCTGAAATGGTGTCCAAACTGGATCTCACTGATGCCAA
	ARID4B-2	XM_006711781.1	4527—4589	NC_012591.1	14400318-14400380	H	TCTGGGAATTGTGTGTCTGGTAACTGGAAGGCCTTCCACTGTGGCAAATGGAGGCTTTTCACT
						O	TCTGGGAATTGTGTGTCTGGTAACTTGAAGGCCTTCCACTGTGGCAAATGGAGGCTTTTCACT
ASF1A	ASF1A-1	NM_014034.2	1954—2024	NC_012597.1	121228965-121229035	H	CAGGCATATTTGTGGTTGACATACTCTAGATAGCTTTTCCAACAAATCTTTGAAAAGCAATCTTGGAAAGG
						O	CAGGCATATTTGTGGTTGACATACTCTAGACAGCTTTTCCAACAAGTCTTTGAAAAGCAATCTTGGAAAGG
	ASF1A-2	NM_014034.2	1631—1696	NC_012597.1	121228642-121228707	H	CAGAGCATTTCTTGAATCATACATCATTATTGTCCAGTGAATTCAAGACCAAATACAATATCGGGA
						O	CAGAGCATTTCTTGAATCATACATCATTATTGCCCAGTGAATTCAAGACCAAATACAATATCGGGA
ASH1L	ASH1L-4	XM_005245336.2	8138—8203	NC_012591.1	96175678-96175743	H	GGGAGAGACAGCAAGTGAGGCAGACAGCAGTGAGACCTCAGTCTCTGAAAAGGAGAATGGGCATGA
						O	GGGAGAGACAGCAAGTGAGGCGGACAGCAGTGAGACCTCAGTCTCTGAAAAGGAGAATGGGCATGA
	ASH1L-5	XM_005245336.2	6854—6926	NC_012591.1	96151629-96151701	H	CGAGCAATGCTGTAACCAGAGGATACAGAGGCATGAATGGGTGCAATGTCTAGAACGATTTCGAGCTGAGGAA
						O	CGAGCAATGCTGTAACCAGAGGATACAGAGGCATGAGTGGGTGCAATGTCTAGAACGATTTCGAGCTGAGGAA
ASXL1	ASXL1-2	XM_006723733.1	2883—2955	NC_012611.1	29634683-29634755	H	ATTGCCTGGGGATTTGACAGCTGAGGAGGGTCTAGATCCTCTTGACAGCCTTACTTCACTCTGGACTGTGCCA
						O	ATTGCCTGGGGATTTGACAGCCGAGGAGGGTCTAGATCCTCCTGACAGCCTTACTTCACTCTGGACTGTGCCA
	ASXL1-3	XM_006723733.1	3100—3163	NC_012611.1	29634900-29634963	H	GACAGCAGTGAGGCTGACACTAGAGAAGCTGCAGTGACAAAGGGATCTTCGGTGGACAAGGATG
						O	GACAGCAGTGAGGCTGACACTAGTGAAGCTGCAGTGACAAAGGGATCTTCGGTGGACAAGGATG
AXIN2	AXIN2-1	XM_005257719.2	1202—1263	NC_012608.1	55165865-55165804	H	GCGCCAACGACAGTGAGATATCCAGTGATGCGCTGACGGATGATTCCATGTCCATGACGGAC
						O	GCGCCAACGACAGTGAGATATCCAGCGATGCGCTGACGGATGATTCCATGTCCATGACGGAC
BAP1	BAP1-3	NM_004656.3	1685—1747	NC_012594.1	93939964-93940026	H	CACCCCCAGCAATGAGAGTACAGACACGGCCTCTGAGATCGGCAGTGCTTTCAACTCGCCACT
						O	CACCCCCAGCAATGAGAGTACGGACACGGCCTCTGAGATCGGCAGTGCTTTCAACTCGCCACT
BMI1	BMI1-1	NM_005180.8	1949—2017	NC_012601.1	23281102-23281170	H	GCATTCTATGTAGCCATGTCACTGTGAATAACGATTTCTTGCATATTTAGCCATTTTGATTCCTGTTTG
						O	GCATTCTATGTAGCCATGTCACTGTGAATAACAATTTCTTGCATATTTAGCCATTTTGATTCCTGTTTG
	BMI1-2	NM_005180.8	1759—1808	NC_012601.1	23280912-23280961	H	AACCATTGTTTGGATTTGGAAGTACTCTGCAGTGGACATAAGCATTGGGC
						O	AACCATTGTTTGGATTTGGAAATACTCTGCAGTGGACATAAGCATTGGGC
BRCA1	BRCA1-1	XM_006722036.1	1216—1277	NC_012608.1	46326580-46326641	H	GAGCGTCCCCTCACAAATAAATTAAAGCGTAAAAGGAGACCTACATCAGGCCTTCATCCTGA
						O	GAGCGTCCCCTCACAAATAAATTAAAGCGTAAAAGGAGAGCTACATCAGGCCTTCATCCTGA
	BRCA1-2	XM_006722036.1	1709—1775	NC_012608.1	46327073-46327139	H	CAGTCAGGCACAGCAGAAACCTACAACTCATGGAAGGTAAAGAACCTGCAACTGGAGCCAAGAAGAG
						O	CAGTCAGGCACAGCAGAAACCTACAACTCATGGAAGATAAAGAACCTGCAACTGGAGCCAAGAAGAG
BRCA2	BRCA2-2	NM_000059.3	2980—3053	NC_012604.1	31872640-31872713	H	ACGAACCCATTTTCAAGAACTCTACCATGGTTTTATATGGAGACACAGGTGATAAACAAGCAACCCAAGTGTCA
						O	ACGAACCCATTTTCAAGAACTCTACCATGGTTTTATATGGAGACATAGGTGATAAACAAGCAACCCAAGTGTCA
	BRCA2-3	NM_000059.3	3653—3715	NC_012604.1	31873310-31873372	H	TGAAGTGCCTGAAAACCAGATGACTATCTTAAAGACCACTTCTGAGGAATGCAGAGATGCTGA
						O	TGAAGTGCCTGAAAACCAGATGACTATCTTAAATACCACTTCTGAGGAATGCAGAGATGCTGA
BRMS1	BRMS1-2	NM_015399.3	1066—1137	NW_002948797.1	15169-15098	H	CTCCTCGAGGCCTGCACAGCTGTGGCCGTGGAGCTGACCTGACCAGGCAAGGCTGCTGTCTCCATCCCTGAG
						O	CTCCTCGAGGCCTGCACAGCTGTGGCCGTGGAGCTGACCTGGCCAGGCAAGGCTGCTGTCTCCATCCCTGAG
	BRMS1-3	NM_015399.3	1365—1437	NW_002948797.1	14870-14798	H	TTCTCAGGGCAGTTCTTGGTGTCTGCTTCTCAGATTCCAAGGACTGGAATTAAAACCTTTCCTGGGACTCTGG
						O	TTCTCAGGGCAGTTCTTGGTGTCTGCCTCTCAGATTCCAAGGACTGGAATTAAAACCTTTCCTGGGACTCTG
CARM1	CARM1-1	XM_005259708.2	1076—1138	NC_012610.1	11068224-11068286	H	TGATGGCCAAGTCTGTCAAGTACACGGTGAACTTCTTAGAAGCCAAAGAAGGAGATTTGCACA
						O	TGATGGCCAAGTCTGTCAAGTACACAGTGAACTTCTTAGAAGCCAAAGAAGGAGATTTGCACA
CBX2	CBX2-1	XM_006722140.1	113—167	NC_012608.1	69815572-69815627	H	AACCGGAAGAGAGGCAAGAGGCCGAGAGGCCGGCCAAGGAAGCTCACTGCCATGT
						O	AACCGGAAGAGAGGCAAGAGGCCAAGAGGCCGGCCAAGGAAGCTCACTGCCATGT
	CBX2-2	XM_006722140.1	2121—2175	NC_012608.1	69819591-69819645	H	GCTGGGGCCTTAATGTGAAAAGACAGTGGCAGGCAGCTGGAGTAGAGCGAGCCCA

						O	GCTGGGGCCTTAATGTGAAAGGACAGTGGCAGGCAGCTGGAGTAGAGCGAGCCCA
CBX4	CBX4-2	NM_003655.2	1543—1591	NC_012608.1	69866976-69866928	H	CGAGGTCATCCTGCTAGACTCAGACCTGGATGAACCCATAGACTTGCGC
						O	CGAGGTCATCCTGCTAGACTCGGACCTGGATGAACCCATAGACTTGCGC
	CBX4-3	NM_003655.2	426—468	NC_012608.1	69868093-69868051	H	GTCGTTCCAATGTCCTGACCGGCCTCCAGGACTCCTCCACTGA
						O	GTCGTTCCAATGTCCTGACCGGACTCCAGGACTCCTCCACTGA
CBX5	CBX5-1	NM_001127322.1	1318—1389	NC_012603.1	54021912-54021841	H	GCTGGCCTTTGTTCTGACATGTTGAGATGGAAAGGATGTTGCCCATCTGTTAAAAAGCCAATAGCAACTGCC
						O	GCTGGCCTTTGTTCTGACATGTTGAGATGGAAAGGATGTTGCTCATCTGTTAAAAAGCCAATAGCAACTGCC
	CBX5-2	NM_001127322.1	1374—1434	NC_012603.1	54021856-54021796	H	GCCAATAGCAACTGCCTACCTGTTGGGGCTTTCCCAACCTTGTTCAGCTCTACCCAGGAGA
						O	GCCAATAGCAACTGCCTACCTGTTGGGGCTTTCCCAGCCTTGTTCAGCTCTACCCAGGAGA
CBX6	CBX6-3	NM_014292.4	1647—1705	NC_012613.1	34066122-34066064	H	TAGGGTTCCTCTGCCCCTTCCACATCCCACCTGTCTCTCTAGCTTGCTTCCTGCTCTCC
						O	TAGGGTTCCTCTGCCCCTTCCACATCCCACCCGTCTCTCTAGCTTGCTTCCTGCTCTCC
	CBX6-4	NM_014292.4	1717—1793	NC_012613.1	34066052-34065976	H	TGATTTCTCGGTGCTAACCTGGCAGCTGTGGGGCCCTTAGGAGCCCCCACCGAGGGTGGACACAGTCCCTTTCCTT
						O	TGATTTCTCGGTGCTAACCTGGCAGCTGTGGGGCCCTTAGGAGACCCCCGCCGAGGGTGGACACAGTCCCTTTCCTT
CBX7	CBX7-3	XM_005261413.2	615—687	NC_012613.1	34331573-34331501	H	AACTCCATCACCGTCACCTTCCGCGAGGCCCAGGCAGCTGAGGGCTTCTTCCGAGACCGCAGTGGGAAGTTCT
						O	AACTCCATCACCGTCACCTTCCGCGAGGCCCAGGCGGCTGAGGGCTTCTTCCGAGACCGCAGTGGGAAGTTCT
CBX8	CBX8-1	NM_020649.2	747—810	NC_012608.1	69829168-69829105	H	CCTCACAGAGGCCCTTAGGCGAACCCAGCGCCGGCCTCGGAGAGTACCTCAAGGGCAGGAAGCT
						O	CCTCACAGAGGCCCTTAGGAGAACCCAGCGCCGGCCTCGGAGAGTACCTCAAGGGCAGGAAGCT
	CBX8-3	NM_020649.2	70—132	NC_012608.1	69831153-69831091	H	CTGCTTTTCTGCTCCTGGAAGCGGCCAAGGGGGGAAGCGGCGAGTCAACATGGAGCTTTCAGC
						O	CTGCTTTTCTGCTCCTGGAAGCGGCCAAGGGGGGAAGCTGCGAGTCAACATGGAGCTTTCAGC
CDK4	CDK4-2	NM_000075.3	1535—1590	NC_012603.1	57577411-57577356	H	CATTGTGCGATTTGGAAAAACCACTTGGAAGAAGGGACTTTCCTGCAAAACCTTAA
						O	CATTGTGCGATTTGGAAAAACCACTTGGAAGGAGGGACTTTCCTGCAAAACCTTAA
	CDK4-3	NM_000075.3	1568—1635	NC_012603.1	57577378-57577311	H	GGGACTTTCCTGCAAAACCTTAAAGACTGGTTAAATTACAGGGCCTAGGAAGTCAGTGGAGCCCCTTG
						O	GGGACTTTCCTGCAAAACCTTAAAGACTGGTTAAGTTACAGGGCCTAGGAAGTCAGTGGAGCCCCTTG
CDX2	CDX2-1	NM_001265.4	1890—1955	NC_012604.1	27404875-27404810	H	AAAGGCTTGGCTGGTGTATGCACAGGGTGTGGTATGAGGGGGTGGTTATTGGACTCCAGGCCTGAC
						O	AAAGGCTTGGCTGGTGTATGCACAGGGTGTGGTATGAGAGGGTGGTTATTGGACTCCAGGCCTGAC
	CDX2-4	NM_001265.4	1577—1637	NC_012604.1	27405188-27405128	H	GAAGCATTGCCTGGACTGACCACACCAACCAGGCCTTCATCCTCCTCCCCAGCTCTTCTCT
						O	GAAGCATTGCCTGGACTGACCACACCAACCAGGCCTTCAGCCTCCTCCCCAGCTCTTCTCT
CHAF1A	CHAF1A-1	NM_005483.2	1962—2041	NC_012610.1	4406403-4406482	H	ATGACGACATGGGAGAGGATGAAGATGAGGACGATGGTTTCTTTGTGCCCCATGGGTACCTGTCTGAGGACGAAGGTGTG
						O	ATGACGACATGGGAGAGGATGAAGATGAGGATGATGGTTTCTTTGTGCCCCACGGGTACCTGTCTGAGGACGAAGGTGTG
	CHAF1A-3	NM_005483.2	1499—1576	NC_012610.1	4404524-4404601	H	AAGTTTGCCCCCTTTGAAATTAAAGAGCACATGGTCCTGGCCCCTCGGCGTCGGACCGCTTTCCATCCAGACCTCTGC
						O	AAGTTTGCCCCCTTTGAAATTAAAGAGCATATGGTCCTGGCCCCTCGGCGTCGGACCGCTTTCCATCCAGACCTCTGC
CHD3	CHD3-1	XM_006721430.1	6442—6525	NC_012608.1	7919177-7919260	H	CTGGCAGGGTCTTCCAAGTACCTTCCTCCCACACTGCCAAGTATACACAACTTCCCAGTAAATGGTTGTGGGGAGGAAAGAGGT
						O	CTGGCAGGGTCTTCCAAGTACCTTCCTCCCCCACTGCCAAGTATACACAACTTCCCAGTAAATGGTTGTGGGGAGGAAAGAGGT
	CHD3-2	XM_006721430.1	6866—6918	NC_012608.1	7919602-7919654	H	CAAGGACCACCCCTACAAAAAGAGTAATGGTTGGGTGATACTCCCTCAAGCCA
						O	CAAGGACCACCCCTACAAAAAGAGTCATGGTTGGGTGATACTCCCTCAAGCCA
CHD4	CHD4-4	NM_001297553.1	4303—4387	NC_012603.1	6795256-6795172	H	TTTAATGCTCGTCAGCGAAAAGCCTTTCTTAATGCAATTATGCGATATGGTATGCCACCTCAGGATGCTTTTACTACCCAGTGGC
						O	TTTAATGCTCGTCAGCGAAAAGCCTTTCTTAATGCAATCATGCGATATGGTATGCCACCTCAGGATGCTTTTACTACCCAGTGGC
CIITA	CIITA-3	XM_006720883.1	2701—2774	NC_012607.1	11249887-11249960	H	CAGGCAGCAGAGGAGAAGTTCACCATCGAGCCTTTCAAAGCCAAGTCCCTGAAGGATGTGGAAGACCTGGGAAA
						O	CAGGCAGCAGAGGAGAAGTTCACCATTGAGCCTTTCAAAGCCAAGTCCCTGAAGGATGTGGAAGACCTGGGAAA
	CIITA-4	XM_006720883.1	3596—3669	NC_012607.1	11265331-11265404	H	AGTTGGGTGGATGCCTGGTGGCAGCTGCGGTCCACCCAGGAGCCCCGAGGCCTTCTCTGAAGGACATTGCGGAC
						O	AGTTGGGTGGATGCCTGGTGGCAGCTGCGGTCCACCCAGGAGCCCTGAGGCCTTCTCTGAAGGACATTGCGGAC
CREBBP	CREBBP-1	XM_005255125.2	732—788	NC_012607.1	3983351-3983295	H	GTACCCTACTCCAGCCATGCAGGGCGCCTCGAGCAGCGTGCTGGCTGAGACCCTAAC
						O	GTACCCTACTCCAGCCATGCAGGGCGCCACAAGCAGTGTGCTGGCTGAGACCCTAAC
	CREBBP-3	XM_005255125.2	4606—4673	NC_012607.1	3862126-3862059	H	CTCAGCTGTGACCTCATGGATGGGCGCGACGCCTTCCTCACCCTCGCCAGAGACAAGCACTGGGAGTT
						O	CTCAGCTGTGACCTCATGGATGGGCGCGACGCCTTCCTCACACTCGCCAGAGACAAGCACTGGGAGTT
CTCF	CTCF-5	XM_005255775.2	1543—1608	NC_012607.1	54989298-54989363	H	AAACGTCACATTCGCTCTCATACTGGAGAGCGTCCGTTTCAGTGCAGTTTGTGCAGTTATGCCAGC
						O	AAACGTCACATTCGCTCTCATACTGGAGAGCGTCCATTTCAGTGCAGTTTGTGCAGTTATGCCAGC
	CTCF-6	XM_005255775.2	353—430	NC_012607.1	54942236-54942313	H	CGCTAGTGGACAGATTGCTGACCAGGGGCTTGAGAGCTGGGTTCTATTTTCCCTCCTCAAACTGACTTTGCAGCCACG
						O	CGCTAGTGGACAGATTGCTGACCAGGGGCTTGAGAGCTGGGTTCTGTTTTCCCTCCTCAAACTGACTTTGCAGCCACG
DGCR8	DGCR8-1	XM_006724268.1	820—884	NC_012613.1	15993113-15993177	H	GTCTACGAGCTGACCAGCAAGGCTGGGCTGTTGTCTCCATATCAGATCCTCCACGAGTGCCTTAA
						O	GTCTACGAGCTGACCAGCAAGGCTGGGCTGTTGTCTCCGTATCAGATCCTCCACGAGTGCCTTAA
	DGCR8-2	XM_006724268.1	1064—1118	NC_012613.1	15994237-15994291	H	AGAACTGGGGGTCTTTACTGCGCATGTATGGCCGTGAGAGCAGCAAGATGGTCAA
						O	AGAACTGGGGGTCTTTACTGCGCATGTACGGCCGTGAGAGCAGCAAGATGGTCAA
DICER1	DICER1-1	NM_001291628.1	3117—3189	NC_012605.1	96515102-96515030	H	TTTTGACACCTCGACATTTGAATCAGAAGGGGAAAGCGCTTCCTTTAAGCAGTGCTGAGAAGAGGAAAGCCAA
						O	TTTTGACACCTCGACATTTGAATCAGAAGGGGAAAGCTCTTCCTTTAAGCAGTGCTGAGAAGAGGAAAGCCAA
	DICER1-5	NM_001291628.1	3884—3962	NC_012605.1	96512955-96512877	H	AGTCCTGTGATGGCCGTAATGCCTGGTACGACAGACACTATTCAAGTGCTCAAGGGCAGGATGGATTCTGAGCAGAGCC
						O	AGTCCTGTGATGGCCGTAATGCCTGGTACGACAGACGCTATTCAAGTGCTCAAGGGCAGGATGGATTCTGAGCAGAGCC
DMAP1	DMAP1-1	XM_006710772.1	971—1025	NC_012591.1	185738272-185738218	H	TGGTGCACATGTTCAATGAGCTGCGAAGCGACCTGGTGCTGCTCTACGAGCTCAA
						O	TGGTGCACATGTTCAATGAGCTGCGAAGTGACCTGGTGCTGCTCTACGAGCTCAA

	DMAP1-2	XM_006710772.1	335—391	NC_012591.1	185743543-185743487	H	GGATGCCATTCACCAACCCGGCCCGCAAGGACGGAGCAATGTTCTTCCACTGGCGAC
						O	GGATGCCATTCACCAACCCGGCCCGCAAGGATGGAGCAATGTTCTTCCACTGGCGAC
DNMT1	DNMT1-4	XM_006722681.1	2409—2479	NC_012610.1	10337242-10337172	H	GCTGGGACAGACACAGTCCTCGGGGCCACGTCGGACCCTCTGGAGCTGTTCTTGGTGGATGAATGTGAGGA
						O	GCTGGGACAGACACAGTCCTCGGGGCCACTTCGGACCCTCTGGAGCTGTTCTTGGTGGATGAATGTGAGGA
	DNMT1-6	XM_006722681.1	3113—3181	NC_012610.1	10329423-10329355	H	GAGCTACCACGCAGACATCAACCTGCTCTACTGGAGCGACGAGGAGGCCGTGGTGGACTTCAAGGCTGT
						O	GAGCTACCACGCAGACATCAACCTGCTGTACTGGAGCGACGAGGAGGCCGTGGTGGACTTCAAGGCTGT
DNMT3A	DNMT3A-2	NM_175630.1	900—978	NC_012592.1	86909282-86909360	H	GTCTGCTTGCCAAGGCTATGGCCTGGAGGCTACTGGCTGGATGCAGCCTGCGCATATGTTTTATTTGGCCCATAGAGTG
						O	GTCTGCTTGCCAAGGCTATGGCCTGGAGGCTACTGGCTGGATGCAGCCTGCACATATGTTTTATTTGGCCCATAGAGTG
	DNMT3A-3	NM_175630.1	1260—1323	NC_012592.1	86909644-86909707	H	ACTCAAGGGCAGCAGATACCCTGTTTGCCTCCCTGAGTGCAGAGGTCCTGAGCCCACCCTAGTT
						O	ACTCAAGGGCAGCAGATACCCTGTTTGCCTCCCTGAGTGCATTAGTCCTGAGCCCACCCTAGTT
	DNMT3A-4	NM_175630.1	1366—1432	NC_012592.1	86909750-86909816	H	GAGAGGGCTGGGTGATTGTATTCTGTGTACTGCCCAGCCCAGGCCTCTTCATCTGGGGACTTTTTGG
						O	GAGAGGGCTGGGTGATTGTATTCTGCGTACTGCCCAGCCCAGGCCTCTTCATCTGGGGACTTTTTG
DNMT3B	DNMT3B-3	NM_001207056.1	1802—1878	NC_012611.1	30007118-30007194	H	TCCTCAAAGAGTTGGGCATAAAGGTAGGAAAGTACGTCGCTTCTGAAGTGTGTGAGGAGTCCATTGCTGTTGGAACC
						O	TCCTCAAAGAGTTGGGCATAAAGGTAGGAAAGTACGTCGCCTCTGAAGTGTGTGAGGAGTCCATTGCTGTTGGAACC
DNMT3L	DNMT3L-1	NM_175867.2	895—1000	NC_012612.1	45760604-45760499	H	GAGCAACTGGGTGTGCTACCTGTGCCTGCCGTCCTCCCGAAGCGGGCTGCTGCAGCGTCGGAGGAAGTGGCGCAGCCAGCTCAAGGCCTTCTACGACCGAGAGTCG
						O	GAGCAACTGGGTGTGCTACCTGTGCCTGCCCTCCTCCCGAAGCGGGCTGCTGCAGCGTCGGAGGAAGTGGCGCAGCCAGCTCAAGGCCTTCTACGACCGAGAGTCG
	DNMT3L-3	XM_006723911.1	204—278	NC_012612.1	45751755-45751681	H	CACCATCCCAGATGTCCACGGCGGATCCTTGCAGAATGCTGTCCGCGTGTGGAGCAACATCCCAGCCATAAGGAG
						O	CACCATCCCAGATGTCCACGGCGGATCCCTGCAGAATGCTGTCCGTGTGTGGAGCAACATCCCAGCCATAAGGAG
DOT1L	DOT1L-3	XM_006722923.1	1141—1212	NC_012610.1	2185702-2185773	H	GCTAAACAAGAAGGGGAGGAAGATGGCTGGCCGCAAGCGCGGGCGCCCCAAGAAGATGAACACTGCGAACCC
						O	GCTAAACAAGAAGGGGAGGAAGATGGCCGGCCGCAAGCGCGGGCGCCCCAAGAAGATGAACACTGCGAACCC
	DOT1L-5	XM_006722923.1	183—233	NC_012610.1	2164129-2164179	H	AGCTTCGAGAGCATGCAGAGGCTCTGCGACAAGTACAACCGTGCCATCGAC
						O	AGCTTCGAGAGCATGCAGAGGCTCTGTGACAAGTACAACCGTGCCATCGAC
EHMT1	EHMT1-1	XM_006717290.1	145—219	NC_012600.1	134750557-134750631	H	AGCAGAGAAACAGGCAGGAGAGGCCCACATGGCTGCGGACGGTGAGACCAATGGGTCTTGTGAAAACAGCGATGC
						O	AGCAGAGAAACAGGCAGGAGAGGCCCACATGGCTGCGGACGGTGAGACCAACGGGTCTTGTGAAAACAGCGATGC
	EHMT1-2	XM_006717290.1	392—467	NC_012600.1	134750804-134750879	H	GGCAGCAACGGATACATCTTAAATAAGCCGGCCCTACAGGCACAGCCCTTGAGGACTACCAGCACTCTGGCCTCTT
						O	GGCAGCAACGGATACATCTTAAATAAGCCAGCCCTGCAGGCACAGCCCTTGAGGACTACCAGCACTCTGGCCTCTT
EHMT2	EHMT2-2	XM_006725982.1	913—988	NC_012597.1	32391985-32391910	H	AGGAGAAGTGACCCTGACGAAAGGGGACCCCGGGTCCCTGGAGGAGTGGGAGACGGTGGTGGGTGATGACTTCAGT
						O	AGGAGAAGTGACCCTGACGAAAGGGGACCCCGGGTCCCTGGAAGAGTGGGAGACGGTGGTGGGTGATGACTTCAGT
	EHMT2-3	XM_006725982.1	2249—2296	NC_012597.1	32385706-32385659	H	GTGGTGAACAACCACCTGGAGGTAGCCCGTTACATGGTGCAGCGTGGT
						O	GTGGTGAACAACCACCTGGAGGTGGCCCGTTACATGGTGCAGCGTGGT
EIF2C1	EIF2C1-1	XM_006710575.1	415—491	NC_012591.1	194299646-194299570	H	GGGCAGCGCTGTATTAAAAAGCTGACCGACAACCAGACCTCGACCATGATAAAGGCCACAGCTAGATCCGCTCCAGA
						O	GGGCAGCGCTGTATTAAAAAGCTGACCGACAACCAGACTTCGACCATGATAAAGGCCACAGCTAGATCCGCTCCAGA
	EIF2C1-4	XM_006710575.1	1235—1287	NC_012591.1	194284739-194284687	H	CCAGCCGATACTGTGCTACTGTGCGGGTACAGCGACCACGGCAAGAGATCATT
						O	CCAGCCGATACTGTGCTACTGTGCGGGTGCAGCGACCACGGCAAGAGATCATT
EIF2C2	EIF2C2-3	NM_001164623.1	1682—1746	NC_012599.1	148814580-148814516	H	GCAGATGAAGAACGTGCAGAGGACCACGCCACAGACCCTGTCCAACCTCTGCCTGAAGATCAACG
						O	GCAGATGAAGAACGTGCAGAGGACCACGCCGCAGACCCTGTCCAACCTCTGCCTGAAGATCAACG
EIF2C3	EIF2C3-1	XM_006710404.1	1111—1189	NC_012591.1	194164079-194164001	H	CCTTCTGTGTTCCAGCAACCAGTGATCTTTTTGGGAGCCGATGTCACTCATCCACCTGCTGGTGATGGAAAGAAGCCTT
						O	CCTTCTGTGTTCCAGCAACCAGTGATCTTTTTGGGAGCTGATGTCACTCATCCACCTGCTGGTGATGGAAAGAAGCCTT
	EIF2C3-2	XM_006710404.1	1549—1626	NC_012591.1	194156939-194156862	H	ATCCCAGCTGGAACAACAGTTGATACAGACATTACACACCCATATGAGTTCGATTTTTACCTCTGTAGCCATGCTGGA
						O	ATCCCAGCTGGAACAACAGTTGATACAGACATTACACACCCATATGAGTTTGATTTTTACCTCTGTAGCCATGCTGGA
EIF2C4	EIF2C4-1	XM_005270579.1	127—183	NC_012591.1	194384189-194384133	H	CCTGGGTATGATGGCAAAAGAAACATGTACACAGCACATCCACTACCAATTGGACGG
						O	CCTGGGTATGATGGCAAAAGAAACATGTACACGGCACATCCACTACCAATTGGACG
	EIF2C4-4	XM_005270579.1	264—326	NC_012591.1	194381659-194381597	H	TGTGAGCCTTCAGTTGCTTTTAGAAGCTTTGGCTGGGCACTTGAATGAAGTCCCAGATGACTC
						O	TGTGAGCCTTCAGTTGCTTTTAGAAGCTTTGGCTGGGCACCTGAATGAAGTCCCAGATGACTC
ELP3	ELP3-3	XM_006716354.1	1384—1447	NC_012599.1	28010662-28010725	H	GCCTCCTACGATTACGCAAGTGTTCAGAAGAAACTTTCCGTTTCGAATTGGGTGGAGGTGTCTC
						O	GCCTCCTACGATTACGCAAGTGTTCAGAGGAAACTTTCCGTTTCGAATTGGGTGGAGGTGTCTC
	ELP3-4	XM_006716354.1	48—116	NC_012599.1	27942791-27942859	H	TCTGAGTTTGTGGCTGCATTTTTATCTCTGGTGGCTCTGCTACGGCGGCGCAGAAATGAGGCAGAAGCG
						O	TCTGAGTTTGTGGCTGCATTTTTATCCCTGGTGGCTCTGCTACGGCGGCGCAGAAATGAGGCAGAAGCG
EP300	EP300-1	XM_006724165.1	5725—5784	NC_012613.1	36442706-36442765	H	CCATGACCACAAAATGGAGAAACTAGGCCTTGGCTTAGATGATGAGAGCAACAACCAGCA
						O	CCATGACCACAAAATGGAGAAACTAGGTCTTGGCTTAGATGATGAGAGCAACAACCAGCA
	EP300-2	XM_006724165.1	6583—6646	NC_012613.1	36443564-36443627	H	GACAGGGATGCAGCAACAGCCACCCTGGAGCCAAGGAGGATTGCCTCAGCCCCAGCAACTACAG
						O	GACAGGGATGCAGCAACAGCCACCCTGGGGCCAAGGAGGATTGCCTCAGCCCCAGCAACTACAG
EP400	EP400-2	NM_015409.4	3326—3390	NC_012603.1	135062402-135062466	H	TCAGAAGATTGGCCTGGACTGGCTGGCCAAACTTTACAGGAAGAATCTCAATGGCATATTGGCAG
						O	TCAGAAGATTGGCCTGGACTGGCTGGCCAAACTCTACAGGAAGAATCTCAATGGCATATTGGCAG
ERAS	ERAS-2	NM_181532.3	442—491	NC_012614.1	49636631-49636680	H	CTCTCGATGACCCCTCGTCTCTGATCCAGCTGCAGCAGATATGGGCCACC
						O	CTCTCGATGACCCCTCGTCTCTGATCCAGCTACAGCAGATATGGGCCACC
	ERAS-3	NM_181532.3	49—110	NC_012614.1	49636238-49636299	H	CACGTCTCTTCCCTGAGCTGCCTGCTGGGGTCATGGAGCTGCCAACAAAGCCTGGCACCTTC

						O	CACGTCTCTTCCCTGAGCTGTCTGCTGGGGTCATGGAGCTGCCAACAAAGCCTGGCACCTTC
ERCC6L	ERCC6L-1	NM_017669.2	236—314	NC_012614.1	69628120-69628042	H	GCAAAGGACATTTTTCCCAATGAAAAAGTGCTGAGCAGAATCCAAAAAATACAGGAAGCCTTGGAGGAGTTGGCAGAAC
						O	GCAAAGGACATTTTTCCCAATGAAAAGGTGCTGAGCAGAATCCAAAAAATACAGGAAGCCTTGGAGGAGTTGGCAGAAC
	ERCC6L-3	NM_017669.2	2035—2096	NC_012614.1	69626321-69626260	H	TGCTGCTCAGAGGAAATCTGATATAAAACTAGATGAACATATTGCCTATCTGCAGTCTTTGG
						O	TGCTGCTCAGAGGAAATCTGATACAAAACTAGATGAACATATTGCCTATCTGCAGTCTTTGG
EZH1	EZH1-2	XM_005257146.1	2308—2385	NC_012608.1	46734926-46735003	H	ACGGCAGCACTTATGGTAGCGGCACTGTCTTGGCTTTCGTGCTCACACCACTGCTGCTCGAGTCTCCTGCACTGTGTC
						O	ACGGCAGCACTTATGGTAGCGGCACTGTCTTGGCTTTCATGCTCACACCACTGCTGCTCGAGTCTCCTGCACTGTGTC
	EZH1-4	XM_005257146.1	1794—1855	NC_012608.1	46730880-46730941	H	ACCCTGACCTGTGTCTCACCTGTGGGGCCTCAGAGCACTGGGACTGCAAGGTGGTTTCCTGT
						O	ACCCTGACCTGTGTCTCACCTGTGGGGCTTCAGAGCACTGGGACTGCAAGGTGGTTTCCTGT
EZH2	EZH2-1	XM_005249964.2	1028—1101	NC_012598.1	146549383-146549310	H	GGATAAAGACCCCACCAAAACGTCCAGGAGGCCGCAGAAGAGGACGGCTTCCCAATAACAGTAGCAGGCCCAGC
						O	GGATAAAGACCCCACCAAAACGCCCAGGAGGCCGCAGAAGAGGACGGCTTCCCAATAACAGTAGCAGGCCCAGC
	EZH2-3	XM_005249964.2	276—355	NC_012598.1	146564034-146563955	H	TCCAACACAAGTCATCCCATTAAAGACTCTGAATGCAGTTGCTTCAGTACCCATAATGTATTCTTGGTCTCCCCTACAGC
						O	TCCAACACAAGTCATCCCATTAAAGACTCTGAATGCAGTTGCTTCAGTGCCCATAATGTATTCTTGGTCTCCCCTACAGC
GATAD2A	GATAD2A-1	NM_001300946.1	2628—2713	NC_012610.1	19932242-19932327	H	CTCCATTGCTGCTTGTTCTGGAGACCCCCGCCCCCGCACCTTCCAGACTTAGCAGAAGAACAAACTGAAGAACAGACCCAGCCAGA
						O	CTCCATTGCTGCTTGTTCTGGAGACCCCCGCCCCCGGACCTCCCAGACTTAGCAGAAGAACAAACTGAAGAACAGACCCAGCCAGA
	GATAD2A-3	NM_001300946.1	1642—1712	NC_012610.1	19928452-19928522	H	TTCAGTCCGTCACCCAAACTGCAGAACTCAGCCTCGGCCACAGCCCTGGTCAGCAGGACTGGCAGACATTC
						O	TTCAGTCCGTCACCCAAACTGCAGAACTCACCCTCGGCCACAGCCCTGGTCAGCAGGACTGGCAGACATTC
GSG2	GSG2-2	NM_031965.2	966—1033	NC_012608.1	3615651-3615718	H	AGAGCATCAGGAGGCCAGTGTTCCCAAGGGCCGCATTGTGCCAAGGGGAATAGACAGGCTGGAGAGAA
						O	AGAGCATCAGGAGGCCAGTGTTCCCAAGGGCCGCATTGTGCCAGGGGGAATAGACAGGCTGGAGAGAA
	GSG2-3	NM_031965.2	1825—1902	NC_012608.1	3616510-3616587	H	CAGCTCTTCATTGTGCTGGAATTTGAGTTTGGAGGGATTGACTTAGAGCAAATGCGAACCAAGTTGTCTTCCTTGGCT
						O	CAGCTCTTCATTGTGCTGGAATTTGAGTTTGGAGGGATTGACTTAGAGCAAATGAGAACCAAGTTGTCTTCCTTGGCT
GTPBP1	GTPBP1-1	XR_244397.1	1114—1175	NC_012613.1	33913072-33913133	H	GCACTGGCACTCAATGTACCTGTCTTTGTGGTAGTCACCAAGATTGACATGTGTCCTGCCAA
						O	GCACTGGCACTCAATGTACCCGTCTTTGTGGTAGTCACCAAGATTGACATGTGTCCTGCCAA
HAT1	HAT1	XM_006712808.1	1011—1070	NC_012593.1	62101923-62101982	H	AGCGGAAGATCCATCCAAAAGCTATGTGAAATTACGAGACTTTGTGCTTGTGAAGCTTTG
						O	AGCGGAAGATCCATCCAAAAGCTATGTAAAATTACGAGACTTTGTGCTTGTGAAGCTTTG
HCFC1	HCFC1-3	XM_006724816.1	861—920	NC_012614.1	154197635-154197576	H	GGAGTCGTCTACCGCTGTGTGAGAAACCGCTTCTCCGTGAGAGCTGCCTTAGACGAAAGG
						O	GGAGTCGTCTACCGCTGTGTGAGGAACCGCTTCTCCGTGAGAGCTGCCTTAGACGAAAGG
HDAC1	HDAC1-2	NM_004964.2	1153—1221	NC_012591.1	197900491-197900423	H	CAGCGACTGTTTGAGAACCTTAGAATGCTGCCGCACGCACCTGGGGTCCAAATGCAGGCGATTCCTGAG
						O	CAGCGACTGTTTGAGAACCTTAGAATGCTGCCGCACGCACCCGGGGTCCAAATGCAGGCGATTCCTGAG
HDAC10	HDAC10-1	NM_001159286.1	1982—2038	NC_012613.1	45914363-45914307	H	AGCTGCATCTTGGGCTTGGTGCTGCCCCTGGCCTATGGCTTCCAGCCTGACCTGGTG
						O	AGCTGCATCTTGGGCTTGGTGCTGCCCCTGGCCTACAGCTTCCAGCCTGACCTGGTG
HDAC11	HDAC11-1	XM_006713338.1	796—865	NC_012594.1	991880-991949	H	CTCAGGCGGGTACCAGAAGCGCACAGCCCGCATCATTGCTGACTCCATACTTAATCTGTTTGGCCTGGGG
						O	CTCAGGCGGGTACCAGAAGCGCACAGCCCGCATCATTGCTGACTCCATCCTTAATCTGTTTGGCCTGGGG
	HDAC11-2	XM_006713338.1	1552—1621	NC_012594.1	992635-992704	H	ATCTGGAGTTCCCCCTCAATAAAGCAAGGTCTGGACCTGCCTTCCCAGGCCCTTCTGTGGGGGTGAAGGT
						O	ATCTGGAGTTCCCCCTCAATAAAGCAAGGTCTGGAGCTGCCTTCCCAGGCCCTTCTGTGGGGGTGAAGGT
HDAC3	HDAC3-1	NM_003883.3	1351—1405	NC_012596.1	143142284-143142230	H	TAAGAGTGGCTTGGGATGCTGTGTCCCAAGGAATTTCTTTTCACCTCTTGGTTGG
						O	TAAGAGTGGCTTGGGATGCTGTGTCCCAGGGAATTTCTTTTCACCTCTTGGTTGG
	HDAC3-3	XM_006714802.1	676—750	NC_012596.1	143150169-143150095	H	GTATGAAGTCGGGGCAGAGAGTGGCCGCTACTACTGTCTGAACGTGCCCCTGCGGGATGGCATTGATGACCAGAG
						O	GTATGAAGTCGGGGCAGAGAGTGGCCGCTACTACTGTCTGAATGTGCCCCTGCGGGATGGCATTGATGACCAGAG
HDAC4	HDAC4-1	XM_006712880.1	3798—3871	NC_012593.1	131650464-131650391	H	AGCCTCACAGGGAACACGGGACAGACGCCGGCGACGCGCAGACACACGGACACGCGGAAGCCAAGCACACTCTG
						O	AGCCTCACAGGGAACACGGGACAGACGCCGGCGACGCGCAGACACACGGACATGCGGAAGCCAAGCACACTCTG
	HDAC4-2	XM_006712880.1	4434—4502	NC_012593.1	131649828-131649760	H	GGCACCAGCCGTCATCTCCGGGGCCAGGGGCTGCAGCCCGGCGGTCCCTGTTTTGCTTTATTGCTGTTT
						O	GGCACCAGCCGTCATCTCCGGGGCCAGGGGCTGCAGCCCGGTGGTCCCTGTTTTGCTTTATTGCTGTTT
HDAC5	HDAC5-1	XM_006721629.1	6723—6791	NC_012608.1	45383693-45383761	H	TCAGCTGGAGCTTCCATACCTTCTGGGCTCCCTTTGCTGACCACCAGCCCAAGGGAGCTAAGACCAGGA
						O	TCAGCTGGAGCTTCCATACCTCCTGGGCTCCCTTTGCTGACCACCAGCCCAAGGGAGCTAAGACCAGGA
	HDAC5-3	XM_006721629.1	139—200	NC_012608.1	45341050-45341111	H	CCGTTCTGAGTGCCTGACTGCCTCGCCCCGAAGGATGGCCTCGGATGGGCATTAGAGGCACG
						O	CCGTTCTGAGTGCCTGACTGCCTCGCCCCGAAGGATGGCCTCTGATGGGCATTAGAGGCACG
HDAC6	HDAC6-1	XM_006724525.1	2896—2974	NC_012614.1	49630173-49630251	H	CACTCCGGACCAGACCACCTCAGAGGAGACTGTGGGAGGAGCCATTCTGGACCAGACCACCTCAGAGGATGCTGTTGGG
						O	CACTCCGGACCAGACCACCTCAGAGGAGACTGTGGGAGGAGCCACTGTGGACCAGACCACCTCAGAGGATGCTGTTGGG
	HDAC6-2	XM_006724525.1	3162—3223	NC_012614.1	49630439-49630500	H	CACCTGTGCAGGGAACTACACCCCAGATATCTCCCAGTACACTGATTGGGAGTCTCAGGACC
						O	CACCTGTGCAGGGAACTACACCCCAGATATTTCCCAGTACACTGATTGGGAGTCTCAGGACC
HDAC7	HDAC7-2	XM_006719458.1	1376—1446	NC_012603.1	47429690-47429620	H	ACACTGTGCTGCTTCCTCTGGCCCAGGGTGGGCACCGGCCTCTGTCCCGGGCTCAGTCTTCCCCAGCCGCA
						O	ACACTGTGCTGCTTCCTCTGGCCCAGGGTGGGCACCGGCCTCTGTCCCGGGCACAGTCTTCCCCAGCCGCA
HDAC8	HDAC8-1	XM_006724674.1	94—163	NC_012614.1	70004756-70004687	H	TTTACATCTGCCAAACCCTTTTCCTTGAACTGTGGTCACTAAATGAGGGGAGTCTGGTCTGGGAAGTCCC
						O	TTTACATCTGCCAAACCCTTTTTCTTGAACTGTGGTCACTAAATGAGGGGAGTCTGGTCTGGGAAGTCCC

	HDAC8-3	XM_006724673.1	987—1052	NC_012614.1	69775925-69775860	H	GGTCCTGATTATGTGCTGGAAATCACGCCAAGCTGCCGGCCAGACCGCAATGAGCCCCACCGAATC
						O	GGTCCTGATTATGTGCTGGAAATCACACCAAGCTGCCGGCCAGACCGCAATGAGCCCCACCGAATC
HDAC9	HDAC9-1	XM_006715803.1	1792—1870	NC_012598.1	65959103-65959025	H	AACCAGGCAGTCACCTTGAGGAAGCAGAGGAAGAGCTTCAGGGGGACCAGGCGATGCAGGAAGACAGAGCGCCCTCTAG
						O	AACCAGGCAGTCACCTTGAGGAAGCGGAGGAAGAGCTTCAGGGGGACCAGGCGATGCAGGAAGACAGAGCGCCCTCTAG
	HDAC9-3	NM_001204146.1	165—209	NC_012598.1	66131508-66131464	H	ACAAAGAAATAACCCCGAAGCACGTTCCTATTTCCCACCTGCTTG
						O	ACAAAGAAATAACCCCGAAGCACATTCCTATTTCCCACCTGCTT
HESX1	HESX1-1	XM_006713379.1	315—384	NC_012594.1	89051660-89051729	H	ATGTCCCAAATCCTCCCAGTGGGATTTCATTCCCTAGCGTGGTGGATCACCCAATGCCAGAAGAAAGAGC
						O	ATGTCCCAAATCCTCCCAGTGGGATTTCATTCCCTAGCATGGTGGATCACCCAATGCCAGAAGAAAGAGC
	HESX1-2	XM_006713379.1	122—194	NC_012594.1	89050670-89050742	H	GACCACGAGAGGATGTCTCCCAGCCTTCAGGAAGGCGCTCAGCTCGGGGAAAACAAACCCTCAACTTGCTCCT
						O	GACCACGAGAGGATGTCTCCCAGCCTTCAGGAAGGCGCTCAGCTTGGGGAAAGCAAACCCTCAACTTGCTCCT
HIF1A	HIF1A-1	NM_001243084.1	2159—2238	NC_012605.1	62497801-62497880	H	ACCACTGATGAATTAAAAACAGTGACAAAAGACCGTATGGAAGACATTAAAATATTGATTGCATCTCCATCTCCTACCCA
						O	ACCACTGATGAATTAAAAACAGTGACAAAAGACCGTATGGAAGACATTAAAATACTGATTGCATCTCCATCTCCTACCCA
	HIF1A-2	NM_001243084.1	1655—1717	NC_012605.1	62495035-62495097	H	CCATTACCCACCGCTGAAACGCCAAAGCCACTTCGAAGTAGTGCTGACCCTGCACTCAATCAA
						O	CCATTACCCACCGCTGAAACACCAAAGCCACTTCGAAGTAGTGCTGACCCTGCACTCAATCAA
HOXA1	HOXA1-2	NM_005522.4	1800—1851	NC_012598.1	57359155-57359206	H	CCTGCAGAGCACAAACTTCAGAAACCCGGCTTCCTGTGCTAAGTCTTTCCCA
						O	CCTGCAGAGCACAAACTTCAGAAACCTGGCTTCCTGTGCTAAGTCTTTCCCA
	HOXA1-5	NM_005522.4	963—1025	NC_012598.1	57358318-57358380	H	AGGGTCTCTTGCCCATCTCTCCGGCCACCCCGCCAGGAAACGACGAGAAGGCCGAGGAATCCT
						O	AGGGTCTCTTGCCCATCTCTCCCGCCACCCCGCCAGGAAACGACGAGAAGGCCGAGGAATCCT
HOXB1	HOXB1-3	NM_002144.3	858—907	NW_002963091.1	1480846-1480797	H	AGACCAGTCGACATGCACCTCCCCGGAAGCCTCACCCAGCTCTGTCACCT
						O	AGACCAGTCGACATGCACCTCCCCAGAAGCCTCACCCAGCTCTGTCACCT
ING1	ING1-4	NM_005537.5	2691—2765	NC_012604.1	113430211-113430285	H	TGCCAGCAAATAAATACGTGTCAAAAAAGAATCTGTATTCAGACCCTGGGTCAGGAAATTACTGCCCACTTGTCA
						O	TGCCAGCAAATAAATACGTGTCAAAAGAGAATCTGTATTCAGACCCTGGGTCAGGAAATTACTGCCCACTTGTCA
INO80	INO80-1	NR_104038.1	4988—5050	NC_012606.1	37392780-37392718	H	GAAGGACAGGCAGGTGGATGGTGTGAGCACTTTTATCATCTGTGTCTCATCAGGAGCATCTGG
						O	GAAGGACAGGCAGGTGGATGATGTGAGCACTTTTATCATCTGTGTCTCATCAGGAGCATCTGG
	INO80-2	NR_104038.1	3535—3579	NC_012606.1	37436559-37436515	H	CTGGAGGTCTGTGGAGCATCAGACCTCAGAATGGCTGGTCTTTCA
						O	CTGGAGGTCTGTGGAGCATCAGGCCTCAGAATGGCTGGTCTTTCA
JARID2	JARID2-3	XM_005249089.1	838—905	NC_012597.1	16084107-16084174	H	TGTCATCTCTGGGTGCAGGTGTAACCAGTGCCAAAAAGATGCGCGAGGTCAGACCTTCACCATCCAAA
						O	TGTCATCTCTGGGTGCAGGTGCAACCAGTGCCAAAAAGATGCGCGAGGTCAGACCTTCACCATCCAAA
	JARID2-5	XM_005249089.1	240—292	NC_012597.1	16039586-16039638	H	GCACAAAGGAAGTTTGCTCAGTCTCAGCCGAATAGTCCCAGCACAACTCCAGT
						O	GCACAAAGGAAGTTTGCTCAATCTCAGCCGAATAGTCCCAGCACAACTCCAGT
JHDM1D	JHDM1D-1	NM_030647.1	2494—2551	NC_012598.1	137318308-137318251	H	GGAAGGTTCATCAGAAATTAGTCAGAGGGTACAAAGTAGGAATTATGTGGACAGCAGC
						O	GGAAGGTTCATCAGAAATTAGTCAGAGGGTACCAAGTAGGAATTATGTGGACAGCAGC
	JHDM1D-2	NM_030647.1	2225—2285	NC_012598.1	137322971-137322911	H	ATGGCAGGGTTGCACTATTCCACGTGTTTACAAAGGCAAATACAAAGCACAGACTGCAGTG
						O	ATGGCAGGGTTGCACTATTCCACATGTTTACAAAGGCAAATACAAAGCACAGACTGCAGTG
JMJD4	JMJD4-1	NM_001161465.1	1421—1487	NC_012591.1	21859000-21859066	H	GCCCTCCCTGTGACCTGGGGCCCACCCCTGTCGAGGCTTGTGGCCTGGCTGTTCATGGCCACTGCCT
						O	GCCCTCCCTGTGACCTGGGGCCCACCCCTGTCGAGGCTCGTGGCCTGGCTGTTCATGGCCACTGCCT
	JMJD4-2	NM_001161465.1	1654—1703	NC_012591.1	21859233-21859282	H	TGTCTGGTGTGCCTTCTCTGGTGGCTCCTCTTAATAGGCCAGCCCTGTCC
						O	TGTCTGGTGTGCCTTCTCTGGAGGCTCCTCTTAATAGGCCAGCCCTGTCC
JMJD6	JMJD6-2	NM_001081461.1	674—743	NC_012608.1	66806560-66806491	H	ACATCGAGTACATGGAGAGCACTCGAGATGATAGTCCCCTTTACATCTTTGACAGCAGCTATGGTGAACA
						O	ACATCGAGTACATGGAGAGCACTCGAGATGACAGTCCCCTTTACATCTTTGACAGCAGCTATGGTGAACA
JMJD7	JMJD7-2	NM_001114632.1	1122—1194	NC_012606.1	38302089-38302161	H	TCAGCTTTGGAGGATCTTGGAATGTGGTCATAAGGACTCAAGGTGCCAGGCAGGTCTGGGTGAGGGTTCTCAG
						O	TCAGCTTTGGAGGATCTTGGAATGTGGTCATAAAGACTCAAGGTGCCAGGCAGGTCTGGGTGAGGGTTCTCAG
	JMJD7-3	NM_001198588.1	406—483	NC_012606.1	38299903-38299980	H	CCCTGGAGTCCTCTATGTGCAGAAGCAGTGCTCCAACCTGCCCAGCGAGCTGCCCCAGCTGCTGCCTGATCTGGAATC
						O	CCCTGGAGTCCTCTATGTGCAGAAGCAGTGCTCCAACCTGCCCACCGAGCTGCCCCAGCTGCTGCCTGATCTGGAATC
JMJD8	JMJD8-1	XM_005255297.2	525—589	NC_012607.1	708014-707950	H	TCTTCGGGGACAACAACTTCACCGAGTGGGCCTCTCTCTTTCGGCACTACTCCCCACCCCCATTT
						O	TCTTCGGGGACAACAACTTCACCGAGTGGGCCTCTCTTTTTCGGCACTACTCCCCACCCCCATTT
	JMJD8-2	XM_005255297.2	442—504	NC_012607.1	708185-708123	H	AGTGGACTTGCCCTTCCAGGAGTATGTGGAGCAGCTGCTGCACCCCCAGGACCCCACCTCCCT
						O	AGTGGACTTGCCCTTCCAGGAGTACGTGGAGCAGCTGCTGCACCCCCAGGACCCCACCTCCCT
KANSL1	KANSL1-5	XM_005257235.2	767—812	NC_012608.1	43738034-43737989	H	TGACAATTCCACTTCCCTCAATGGGGGAAAACGGGCTCTCACTTCA
						O	TGACAATTCCACTTCCCTCAATGAGGGAAAACGGGCTCTCACTTCA
KAT2A	KAT2A-1	XM_006721818.1	1185—1242	NC_012608.1	47331919-47331976	H	GGGTTTCTCCAAGGACATCAAGGTGCCCAAGAGCCGCTACCTGGGCTACATCAAGGAC
						O	GGGTTTCTCCAAGGACATCAAGGTGCCTAAGAGCCGCTACCTGGGCTACATCAAGGAC
	KAT2A-3	XM_006721818.1	1367—1436	NC_012608.1	47332397-47332466	H	AGATCCGCAAGGTCTACCCGGGGCTCAGCTGCTTCAAGGAGGGCGTGAGGCAGATCCCTGTGGAGAGCGT
						O	AGATCCGCAAGGTCTACCCGGGGCTCAGCTGCTTCAAGGAGGGTGTGAGGCAGATCCCTGTGGAGAGCGT
KAT2B	KAT2B-2	XR_245162.2	974—1034	NC_012594.1	127357710-127357650	H	CCACAGTTCTGCGACAGTCTACCTCGGTACGAAACCACACAGGTGTTTGGGAGAACATTGC
						O	CCACAGTTCTGCGACAGTCTACCTCGGTATGAAACCACACAGGTGTTTGGGAGAACATTGC
KAT5	KAT5-1	XM_006718421.1	935—1010	NC_012602.1	10183997-10183922	H	GACTGGCAGCCTGGTGTCTGATCGAAGCCACGACGACATCGTCACCCGGATGAAGAACATTGAGTGCATTGAGCTG

						O	GACTGGCAGCCTGGTGTCTGACCGAAGCCACGACGACATCGTCACCCGGATGAAGAACATTGAGTGCATTGAGCTG
KAT6A	KAT6A-1	XM_005273649.1	4672—4726	NC_012599.1	42444829-42444775	H	CCGTGGTGTCAGAGCAGATGGCTGGGTCTGAGGACGACCACGAAGAAGACTCCCA
						O	CCGTGGTGTCAGAGCAGATGCCTGGGTCTGAGGACGACCACGAAGAAGACTCCCA
	KAT6A-2	XM_005273649.1	4920—4998	NC_012599.1	42444581-42444503	H	CCTCAGATGTCCATGGTTGAAGACTGTCATGCGTCAGAACATAATAGCCCTATCTCCTCCGTTCAGTCTCACCCCAGCC
						O	CCTCAGATGTCCATGGTTGAAGACTGTCATGCGTCAGAACATAATAGCCCTATCTCCTCTGTTCAGTCTCACCCCAGCC
KAT6B	KAT6B-2	XM_005269664.1	5164—5233	NC_012601.1	60130479-60130410	H	CACTCGAACCCAGAGGTCTTAATGGACTGTGGCGTCGACCTGACAGCTTCTTGTAACAGTGAGCCCAAGG
						O	CACTCGAACCCAGAGGTCTTAATGGACTGTGGTGTCGACCTGACAGCTTCTTGTAACAGTGAGCCCAAGG
	KAT6B-3	XM_005269664.1	6096—6161	NC_012601.1	60129547-60129482	H	CATGCAGCTGGCTGAAATCCCCGAGACGAGCAACGCCAACATTGGCTTATACGAGCGAATGGGTCA
						O	CATGCAGCTGGCTGAAATCCCCGAGACGGGCAACGCCAACATTGGCTTATACGAGCGAATGGGTCA
KAT7	KAT7-1	NM_007067.4	592—653	NW_002963091.1	2820658-2820719	H	CCTCGAACTCCAACTGGAAATGCGCCTTCTTCTGAGTCTGACATAGACATCTCCAGCCCCAA
						O	CCTCGAACTCCAACTGGAAACGCACCTTCTTCTGAGTCTGACATAGACATCTCCAGCCCCAA
	KAT7-2	NM_007067.4	635—686	NW_002963091.1	2820701-2820752	H	TAGACATCTCCAGCCCCAATGTATCTCACGATGAGAGCATTGCCAAGGACAT
						O	TAGACATCTCCAGCCCCAATGTGTCTCACGATGAGAGCATTGCCAAGGACAT
KAT8	KAT8-1	NM_182958.2	351—408	NC_012607.1	29656352-29656409	H	GACCAAGACAGTGAAGGATGCTGTACAGAAGAACTCAGAGAAGTACCTGAGCGAGCTC
						O	GACCAAGACAGTGAAGGATGCCGTGCAGAAGAACTCAGAGAAGTACCTGAGCGAGCTC
KCTD3	KCTD3-1	XM_005273158.1	2323—2392	NC_012591.1	34270703-34270634	H	GTAGGTGGTCCAACCGAAGAAGAGCTACTCAAATTACTCGATCAATGTGATTTGAGCACATCTCGCTGTG
						O	GTAGGTGGTCCAACCGAAGAAGAGCTACTCAAATTACTGGATCAATGTGATTTGAGCACATCTCGCTGTG
	KCTD3-2	XM_005273158.1	1219—1294	NC_012591.1	34303378-34303303	H	TGGCAGCAAGTGTTTACGAGCCCATATTTGGATTGGACTATCGAACGAGTAGCTTTAAATGCAAAGGTGGTTGGAG
						O	TGGCAGCAAGTGTTTACGAGCCCATATTTGGATTGGACCATCGAACGAGTAGCTTTAAATGCAAAGGTGGTTGGAG
KDM1A	KDM1A-1	XM_006710474.1	2100—2164	NC_012591.1	207353874-207353810	H	GTACCCTTCCCCTGGGTGTGCTGAAGCAGCAGCCACCAGCCGTTCAGTTTGTGCCACCTCTCCCT
						O	GTACCCTTCCCCTGGGTGTGCTGAAGCAGCAGCCACCAGCTGTTCAGTTTGTGCCACCTCTCCCT
	KDM1A-3	XM_006710474.1	1361—1440	NC_012591.1	207364819-207364740	H	AACCGGTTGCTAGAAGCTACATCTTACCTTAGTCATCAACTAGACTTCAATGTCCTCAATAATAAGCCTGTGTCCCTTGG
						O	AACCGGTTGCTAGAAGCTACATCTTACCTTAGTCATCAGCTAGACTTCAATGTCCTCAATAATAAGCCTGTGTCCCTTGG
KDM1B	KDM1B-2	XM_006715020.1	2374—2443	NC_012597.1	18954466-18954535	H	TGTGAAGACAGGTGGAAGTGGGGAGGCCTACGATATCATTGCTGAAGACATTCAAGGAACCGTCTTTTTC
						O	TGTGAAGACAGGTGGAAGTGGGGAGGCCTATGATATCATTGCTGAAGACATTCAAGGAACCGTCTTTTTC
	KDM1B-3	XM_006715020.1	1970—2032	NC_012597.1	18949172-18949234	H	GGTGCCATTCAGTTTAATCCACCGTTGTCAGAGAAGAAGATGAAGGCTATCAACAGCTTAGGC
						O	GGTGCCATTCAGTTTAATCCACCATTATCAGAGAAGAAGATGAAGGCTATCAACAGCTTAGGC
KDM2A	KDM2A-1	XM_006718481.1	5119—5189	NC_012602.1	8654302-8654232	H	CCCTGGGTATTGAGCAAAAACCTTATTATCGTTAATGACCTATAATTGGAAGCTTCCTGCCTTTTTCTTTG
						O	CCCTGGGTATTGAGCAAAAACCTTATTATTGTTAATGACCTATAATTGGAAGCTTCCTGCCTTTTTCTTTG
	KDM2A-2	XM_006718481.1	3150—3226	NC_012602.1	8656211-8656135	H	CCTGTCTGACGAGAAGCTGATACAGAAGATCAGCTAAGACACACCCAGCCCAGATTCAACAGGAAACCGATCTTCCC
						O	CCTGTCTGACGAGAAGCTGATACAGAAGATCAGCTAAGACACACCCAGCCCAGACTCAACAGGAAACCGATCTTCCC
KDM2B	KDM2B-1	XM_005253961.2	2573—2646	NC_012603.1	123717251-123717178	H	GGATGAGTGAGCCGACACTTTCCTTTGGTCTTTCTGAATCGTAACTGCACTGCTTTCTGGACCATTTCTAAGGC
						O	GGATGAGTGAGCCGACACTTTCCTTTGGTCTTTCTAAATCGTAACTGCACTGCTTTCTGGACCATTTCTAAGGC
KDM3A	KDM3A-1	NM_001146688.1	1157—1233	NC_012592.1	23627719-23627643	H	GGGTTGCTCTCAAAGTCCTCTCAGATTGGAACTGGAGACTTGAAAATTCTGACTGAGCCAAAAGGCAGCTGTACTCA
						O	GGGTTGCTCTCAAAGTCCTCTCAGATTGGAACTGGAGACTTGAAAATTCTGAGTGAGCCAAAAGGCAGCTGTACTCA
	KDM3A-3	NM_001146688.1	1665—1733	NC_012592.1	23623896-23623828	H	GTGTGAACATCGTGGCACAGTTGCCTAAATGCCGAGAGTGTCGCTTGGACAGTCTCCGCAAGGATAAGG
						O	GTGTGAACATCGTGGCACAGTTGCCTAAGTGCCGAGAGTGTCGCTTGGACAGTCTCCGCAAGGATAAGG
KDM3B	KDM3B-2	XM_005272018.2	4847—4899	NC_012596.1	139984561-139984613	H	CAGCCACTCTCTTCTACGCTGCCTCAACACTGAAGGTTGACACAGGAAAGTCG
						O	CAGCCACTCTCTTCTACGCTGCCTCAACACCGAAGGTTGACACAGGAAAGTCG
	KDM3B-3	XM_005272018.2	3536—3591	NC_012596.1	139972415-139972470	H	GGTGATGGGGTTAAATGTGCTAGATCCCCATACTTCTCACTCCTGGCTTTGTGATG
						O	GGTGATGGGGTTAAATGTGCTAGATCCGCATACTTCTCACTCCTGGCTTTGTGATG
KDM4A	KDM4A-1	XM_005271356.1	3722—3797	NC_012591.1	186209869-186209794	H	AACTATGCTGGCTGGACTGGCTGCCTTGTTCCTGGCCTAGGACTTAGCTTCATAACTATCACCTGCACCGACTAGG
						O	AACTATGCTGGCTGGACTGGCTGCCTTGTTCCTGGCCTAGGACTTAGCTTCGTAACTATCACCTGCACCGACTAGG
	KDM4A-3	XM_005271356.1	2758—2825	NC_012591.1	186220009-186219942	H	GAAAAGAACTGCTGGCTGCTGTGTGCAGTGTTCTCACGGCCGCTGCCCAACTGCCTTCCATGTGAGCT
						O	GAAAAGAACTGCTGGCTGCTGTGTGCAGTGTTCTCATGGCCGCTGCCCAACTGCCTTCCATGTGAGCT
KDM4B	KDM4B-1	XM_005259522.2	583—648	NC_012610.1	5132954-5133019	H	CCATCATCCCCATGCTGTACGTGGTGCCGCGGCCGGGCAAGGCAGCCTTCAACCAGGAGCACGTGT
						O	CCATCATCCCCATGCTGTACGTGGTTCCTCGGCCAGGCAAGGCAGCCTTCAACCAGGAGCACGTGT
	KDM4B-2	XM_005259522.2	1015—1089	NC_012610.1	5137248-5137322	H	AGGCCGAGAGGAAGTTCAACGCAGCGGCTGCGCGCACGGAGCCCTACTGCGCCATCTGCACGCTCTTCTACCCCT
						O	AGGCCGAGAGGAAGTTCAACGCGGCGGCTGCGCGCACGGAGCCCTACTGCGCCATCTGCACGCTCTTCTACCCCT
KDM4C	KDM4C-4	NR_130707.1	1576—1650	NC_012600.1	55830316-55830242	H	GCTTTGGAAACAAGGAAAGGATATATACACCATTGATCACACGAAGCCTACTCCAGCATCCACCCCTGAAGTAAA
						O	GCTTTGGAAACAAGGAAAGGATATATACACCATTGATCACACAAAGCCTACTCCAGCATCCACCCCTGAAGTAAA
KDM4D	KDM4D-1	NM_018039.2	861—916	NC_012602.1	90986900-90986955	H	GTGCCCAGAATCCAAATTGTAACATAATGATATTTCATCCAACCAAAGAAGAGTTT
						O	GTGCCCAGAATCCAAATTGTAACGTAATGATATTTCATCCAACCAAAGAAGAGTTT
	KDM4D-4	NM_018039.2	1704—1754	NC_012602.1	90987743-90987793	H	AGGCCATCAATTTTGCCACTCCGCGATGGATTGATTATGGCAAAATGGCCT
						O	AGGCCATCAATTTTGCCACTCCACGATGGATTGATTATGGCAAAATGGCCT
KDM5A	KDM5A-1	NM_001042603.2	5470—5536	NC_012603.1	266805-266739	H	CATGGACCACATTGAGACCTTAGTCATCAAGTAGAGTGGTTTATATCCACTTGGAATGTTGCTTCCA
						O	CATGGACCACATTGAGACCTTAGTCATCAAGTAGAGTGGTTGATATCCACTTGGAATGTTGCTTCCA

	KDM5A-3	NM_001042603.2	4118—4183	NC_012603.1	291974-291909	H	CGTGCTATGAGTTGGCAAGATAGAGCGCGGCAGGCTCTAGCCACAGATGAACTATCCTCTGCCCTG
						O	CGTGCTATGAGTTGGCAAGATAGAGCACGGCAGGCTCTAGCCACAGATGAACTATCCTCTGCCCTG
KDM5B	KDM5B-1	NM_006618.3	2013—2085	NC_012591.1	47555436-47555508	H	ATCTGCAAGATGGCTTCCAAGGCTGATGTATTAGATGTTGTAGTGGCTTCAACTGTTCAGAAAGACATGGCCA
						O	ATCTGCAAGATGGCTTCCAAGGCTGATGTACTAGATGTTGTAGTGGCTTCAACTGTTCAGAAAGACATGGCCA
	KDM5B-3	NM_006618.3	1320—1385	NC_012591.1	47546897-47546962	H	CCCACAGAGCTTGTTGAGAAAGAATTTTGGAGACTAGTAAGCACTATTGAGGAGGATGTCACAGTG
						O	CCCACAGAGCTTGTTGAGAAAGAATTTTGGAGACTGGTAAGCACTATTGAGGAGGATGTCACAGTG
KDM6A	KDM6A-1	NR_111960.1	2445—2498	NC_012614.1	45723032-45723085	H	GAGAGCAAGCCTTCAGGAAACATATTGACGGTGCCTGAAACAAGCAGGCACACT
						O	GAGAGCAAGCCTTCAGGAAACATATTGACAGTGCCTGAAACAAGCAGGCACACT
KDM6B	KDM6B-4	XM_006721484.1	4302—4382	NC_012608.1	7852921-7853001	H	ACTGGGATCTGACAGGCACTCGGCAGATCTGGCCTTGTGAGAGCTCCCGTTCCCACACCACCATTGCCAAGTACGCACAGT
						O	ACTGGGATCTGACAGGCACTCGGCAGATCTGGCCCTGTGAGAGCTCCCGTTCCCACACCACCATTGCCAAGTACGCACAGT
KDM8	KDM8-1	XM_006721093.1	444—494	NC_012607.1	26463381-26463431	H	CAGGTTGACGTGGAGAATCCCGACCTGGAAAAGTTCCCCAAGTTTGCCAAG
						O	CAGGTTGACGTGGAGAATCCCGACCTGGAGAAGTTCCCCAAGTTTGCCAAG
	KDM8-4	XM_006721092.1	602—688	NC_012607.1	26456418-26456504	H	GGACCATGGTTTGATTCCAGATGTGAAGTTAGAAAAAACAGTCCCCCGGCTGCACCGTCCGTCCCTCCAGCATTTCAGGGAGCAGTT
						O	GGACCATGGTTTGATTCCAGATGTGAAGTTAGAAACAACAGTCCCCCGGCTGCACCGTCCGTCCCTCCAGCATTTCAGGGAGCAGTT
LBR	LBR-1	XM_005273125.1	1814—1893	NC_012591.1	24306913-24306992	H	CGTGTGCCCTACCGTATATTTCCATACATCTACTAATGCTCTTCTGGCTTTTCTACAAAATACTCCTGCAATTCCAGCTG
						O	CGTGTGCCCTACCGTATATTTCCATACATCTACTAATGCTCTTCTGGCTTATTTACAAAATACTCCTGCAATTCCAGCTG
	LBR-3	XM_005273125.1	389—467	NC_012591.1	24288182-24288260	H	GGTCGACCACCTAAAAGTGCCCGCCGATCTGCTTCTGCTTCCCACCAGGCCGACATTAAGGAAGCAAGGAGGGAAGTGG
						O	GGTCGACCACCTAAAAGTGCCCGTCGATCCGCTTCTGCTTCCCACCAGGCCGACATTAAGGAAGCAAGGAGGGAAGTGG
LEFTY2	LEFTY2-3	NM_003240.3	1614—1690	NC_012591.1	23731184-23731260	H	CAGCTGGGAGTTTCTGTTCTCTGGCAAATTCTTCACTGAGTCTGGAACAATAATACCCTATGATTAGAACTGGGGAA
						O	CAGCTGGGAGTTTCTGTTCTCTGGCAAATTCCTCACTGAGTCTGGAACAATAATACCCTATGATTAGAACTGGGGAA
	LEFTY2-4	NM_003240.3	1684—1749	NC_012591.1	23731254-23731319	H	TGGGGAAACAGAACTGAATTGCTGTGTTATATGAGGAATTAAAACCTTCAAATCTCTATTTCCCCC
						O	TGGGGAAACAGAACTGAATTGCTCTATTACATGAGGAATTAAAACCTTCAAATCTCTATTTCCCCC
LMNA	LMNA-7	NM_001282626.1	1072—1132	NC_012591.1	95365795-95365735	H	AGGCAGTCTGCTGAGAGGAACAGCAACCTGGTGGGGGCTGCCCACGAGGAGCTGCAGCAGT
						O	AGGCAGTCTGCTGAGAGGAACAGCAACCTGGTGGGGGCCGCCCACGAGGAGCTGCAGCAGT
	LMNA-8	NM_001282626.1	1667—1722	NC_012591.1	95364047-95363992	H	ATGGAGATGATCCCTTGCTGACTTACCGGTTCCCACCAAAGTTCACCCTGAAGGCT
						O	ATGGAGATGATCCCTTGCTGACTTACCGCTTCCCACCAAAGTTCACCCTGAAGGCT
LMNB1	LMNB1-2	NM_001198557.1	925—999	NC_012596.1	128186227-128186301	H	GTGACAGTATCCCGAGCATCCTCAAGTCGTAGTGTACGTACAACTAGAGGAAAGCGGAAGAGGGTTGATGTGGAA
						O	GTGACAGTATCCCGAGCATCCTCAAGTCGTAGTGTACGTACAACTAGAGGAAAACGGAAGAGGGTTGATGTGGAA
	LMNB1-3	NM_001198557.1	487—543	NC_012596.1	128175449-128175505	H	CAACATGATGCCCAAGTGAGGCTGTATAAGGAGGAGCTGGAGCAGACTTACCATGCC
						O	CAACATGATGCCCAAGTGAGGCTGTATAAGGAAGAGCTGGAGCAGACTTACCATGCC
LMNB2	LMNB2-1	NM_032737.3	1879—1940	NC_012610.1	2415773-2415712	H	GAGGCTGCTACGTGATGTGAACCCACACTCCTCATCCACACACCTTTCTTTACCCAGAGCCA
						O	GAGGCTGCTACGTGATGTGAACCCACACTCCTCATCCACTCACCTTTCTTTACCCAGAGCCA
	LMNB2-2	NM_032737.3	3639—3688	NC_012610.1	2414086-2414037	H	GGTGGGTCTTGGTGATCTTGTCCTATGGGGAGTCTGCAGTGGTCTCAGCC
						O	GGTGGGTCTTGGTGATCTTGTCCTGTGGGGAGTCTGCAGTGGTCTCAGCC
LMX1B	LMX1B-4	NM_001174147.1	1375—1450	NC_012600.1	123578602-123578677	H	GGTACAGCCAGACCGGTAGATGGGCACAGCCTGGGCAGGGGCTGTGTCCTGCCCACAGAGACCTTGTCATCCCCAG
						O	GGTACAGCCAGACCGGTAGATGGGCACAGCCTGGACAGGGGCTGTGTCCTGCCCACAGAGACCTTGTCATCCCCAG
MAD1L1	MAD1L1-2	NM_001304525.1	732—811	NC_012598.1	1833796-1833717	H	TCCAGACCAAGATCCAGGAGTTCCGCAAGGCCTGCTACACGCTCACCGGCTACCAGATCGACATCACCACGGAGAACCAG
						O	TCCAGACCAAGATCCAGGAGTTCCGCAAGGCCTGCTACACGCTCACCGGCTACCAGATTGACATCACCACGGAGAACCAG
	MAD1L1-3	NM_001304524.1	177—227	NC_012598.1	2144042-2143992	H	AACAGGCAGTGTCAGCAGAACTTGGATGCTGCCAGCAAGAGGCTGCGTGAG
						O	AACAGGCAGTGTCAGCAGAACCTGGATGCTGCCAGCAAGAGGCTGCGTGAG
MAK	MAK-1	XR_427831.1	1930—1993	NC_012597.1	11135574-11135511	H	GCAACCAGTTATTCCCCAAGTCACTGGGACCCGTTGGGGCAGAACTTGCTTTCAAAAGGAGCAA
						O	GCAACCAGTTATTCCCCAAGTCACTGGGACCCGTCGGGGCAGAACTTGCTTTCAAAAGGAGCAA
	MAK-2	XM_006715098.1	1580—1652	NC_012597.1	11144389-11144317	H	TCGACAGGGGAAAACAAGAGCTTACCTGCTGTTACTTCCCTAAAATCTGATTCCGAATTGTCAACTGCTCCAA
						O	TCGACAGGGGAAAACAAGAGCTTACCTGCTGTTATTTCCCTAAAATCTGATTCCGAATTGTCAACTGCTCCAA
MBD1	MBD1-3	XM_006722474.1	2394—2444	NC_012609.1	62654865-62654815	H	TTTCATGGTGATTCCAGCAAGTACAGAGATTCTATGAAGCCCACCCAGAAA
						O	TTTCATGGTGATTCCAGCAAGTGCAGAGATTCTATGAAGCCCACCCAGAAA
	MBD1-4	XM_006722475.1	1410—1468	NC_012609.1	62659392-62659334	H	CATCTTGGCCCTACCTTGAAGCCCACCTTGGCTACACGCACAGCCCAACCAGACCATAC
						O	CATCTTGGCCCTACCTTGAAGCCCACCTTGGTTACACGCACAGCCCAACCAGACCATAC
MBD2	MBD2-1	NM_003927.4	2377—2461	NC_012609.1	66798039-66797955	H	GGCTACAGTGAGAGGCAGCTTCTAGACAGAGTTGCTTAATGAAAGGGTTTGTAATACTTTACAAACCATTACCTGTACCTGGCCT
						O	GGCTACAGTGAGAGGCAGCTTCTAGGCAGAGTTGCTTAATGAAAGGGTTTGTAATACTTTACAAACCATTACCTGTACCTGGCCT
	MBD2-3	NM_003927.4	3570—3622	NC_012609.1	66796828-66796776	H	TCCCTTGCTTCTGACTTCTCATCAATCCTATTAGAACTACTGACTGCACCCCA
						O	TCCCTTGCTTCTGACTTCTCATGAATCCTATTAGAACTACTGACTGCACCCCA
MBD3	MBD3-1	NM_001281453.1	806—873	NC_012610.1	1524611-1524544	H	AGGAAGCAGGAAGAGCTGGTGCAGCAGGTGCGGAAGCGGCTGGAGGAGGCGCTGATGGCCGACATGCT
						O	AGGAAGCAGGAAGAGCTGGTGCAGCAGGTGCGGAAGCGCCTGGAGGAGGCGCTGATGGCCGACATGCT
	MBD3-3	NM_001281453.1	294—354	NC_012610.1	1531268-1531208	H	GCTCCATGGACCTGAGCACCTTCGACTTCCGCACGGGCAAGATGCTGATGAGCAAGATGAA
						O	GCTCCATGGACCTGAGCACTTTCGACTTCCGCACGGGCAAGATGCTGATGAGCAAGATGAA
MBD4	MBD4-2	NM_001276273.1	1094—1156	NC_012594.1	3216275-3216337	H	GACTGGCTTTGGGAAAATCATGAAAAATTAAGTCTATCTTAAACTCTGCAGCTTTCAAGCTCA
						O	GACTGGCTTTGGGAAAATCATGAAAAATTAAGTCTGTCTTAAACTCTGCAGCTTTCAAGCTCA

	MBD4-3	NM_001276273.1	707—773	NC_012594.1	3213877-3213943	H	ACACCTCCTCGGTCACCTTTTAATCTCGTTCAAGAAACACTTTTTCATGATCCATGGAAGCTTCTCA
						O	ACACCTCCTCGGTCACCTTTTAATCTCATTCAGGAAACACTTTTTCATGATCCATGGAAGCTTCTCA
MECP2	MECP2-1	XM_005274683.2	2763—2824	NC_012614.1	154262021-154261960	H	GGGAAAAGCCTTTCGCTCTAAAGTGGAGTTGATTGCGTACTTCGAAAAGGTAGGCGACACAT
						O	GGGAAAAGCCTTTCGCTCTAAAGTGGAGCTGATTGCGTACTTCGAAAAGGTAGGCGACACAT
	MECP2-3	XM_006724819.1	406—478	NC_012614.1	154261513-154261441	H	GAGACCGTACTCCCCATCAAGAAGCGCAAGACCCGGGAGACGGTCAGCATCGAGGTCAAGGAAGTGGTGAAGC
						O	GAGACCGTACTCCCCATCAAGAAGCGCAAGACCCGGGAGACAGTCAGCATCGAGGTCAAGGAAGTGGTGAAGC
MEIS1	MEIS1-2	XM_006712020.1	527—600	NC_012592.1	44317358-44317285	H	ACGGCATCTACTCGTTCAGGAGGAACCCCAGGCCCTTCCAGCGGTGGCCACACGTCACACAGTGGGGACAACAG
						O	ACGGCATCTACTCGTTCAGGAGGAACCCCAGGCCCTTCCAGCGGTGGCCACACATCACACAGTGGGGACAACAG
	MEIS1-4	XM_006712020.1	1180—1239	NC_012592.1	44209424-44209365	H	AATGCCAATGTCAGCATCAAGCCCCACAGTTCTTAATACAGGAGACCCAACAATGAGTGG
						O	AATGCCAATGTCAGCATCAAGCCCCACGGTTCTTAATACAGGAGACCCAACAATGAGTGG
MEOX2	MEOX2-2	NM_005924.4	388—462	NC_012598.1	69048933-69049007	H	TGTCTTACCCCGAGCTCTCTACTTCTTCCTCATCTTGCATAATCGCGGGATACCCCAACGAAGAGGGCATGTTTG
						O	TGTCTTACCCCGAGCTCTCTACTTCTTCCTCATCTTGCATAATCGCGGGATACTCCAACGAAGAGGGCATGTTTG
	MEOX2-4	NM_005924.4	1122—1183	NC_012598.1	69124272-69124333	H	CAGCAAACAGGGGACTCTATAGCAAATGAAGACAGTCACGACAGTGACCACAGCTCAGAGCA
						O	CAGCAAACAGGGGACTCTCTAGCAAATGAAGACAGTCACGACAGTGACCACAGCTCAGAGCA
MLL	MLL-5	XM_006718840.1	1662—1726	NC_012602.1	115439076-115439140	H	AAATCATTCCAGCTCCCAAACCCAAAGGTCCTGGAGAACCAGACTCACCAACTCCTCTGCATCCT
						O	AAATCATTCCAGCTCCCAAACCCAAAGGGCCTGGAGAACCAGACTCACCAACTCCTCTGCATCCT
MTA1	MTA1-1	NM_001203258.1	1056—1113	NC_012605.1	107241534-107241591	H	CATCAGAGGCCAACCTTTTCGAGGAAGCCCTGGAAAAATATGGGAAGGATTTCACGGA
						O	CATCAGAGGCCAACCTTTTCGAGGAAGCCCTAGAAAAATATGGGAAGGATTTCACGGA
	MTA1-2	NM_001203258.1	744—832	NC_012605.1	107239839-107239927	H	AGGATGGCCGAGACCAGTCCAGGTTGGAGACCCAGGTGTGGGAGGCGCACAACCCACTCACAGACAAGCAGATCGACCAGTTCCTGGTG
						O	AGGATGGCCGAGACCAGTCCAAGCTGGAGACCAAGGTGTGGGAGGCGCACAACCCACTCACAGACAAGCAGATCGACCAGTTCCTGGTG
MTA2	MTA2-1	NM_004739.3	2547—2619	NC_012602.1	13581676-13581748	H	CTGGTGTTACCACCTCGAGACTTGTCTCATGCCTCCATGCTTGCCGATGGAGGACAGACTGCAGGAACTTGGC
						O	CTGGTGTTACCACCTCGAGACTTGTCTCTTGCCTCCATGCTTGCCGATGGAGGACAGACTGCAGGAACTTGGC
	MTA2-2	NM_004739.3	1534—1603	NC_012602.1	13579534-13579603	H	GGCCCACCTAACATGCAGTGCCGCCTCTGTGCTTCCTGTTGGATCTACTGGAAGAAGTATGGGGGACTGA
						O	GGCCCACCTAACATGCAGTGCCGCCTTTGTGCTTCCTGTTGGATCTACTGGAAGAAGTATGGGGGACTGA
MTA3	MTA3-2	XM_005264459.2	1600—1649	NC_012592.1	68763582-68763533	H	CACGTTCCAGCCTCAGAATCCTCTCTTAGGGAGAGCCTGTGAGAGCTGCT
						O	CACGTTCCAGCCTCAGAATCCTCTTTTAGGGAGAGCCTGTGAGAGCTGCT
MTF2	MTF2-2	NM_001164393.1	2866—2919	NC_012591.1	135654309-135654256	H	TTGCACAGGGTTAACAAACAGTATGTTGCCAGCTGAGGCTACTGCTGTTTTATT
						O	TTGCACAGGGTTAACAAACAGTATGTTGCCGGCTGAGGCTACTGCTGTTTTATT
	MTF2-3	NM_001164393.1	869—930	NC_012591.1	135671743-135671682	H	TGATTCTGAACTTGAGCTTATGACATACATTAATGAAAACTGGGATAGATTGCACCCTGGAG
						O	TGATTCTGAACTTGAGCTTATGACATACATTAACGAAAACTGGGATAGATTGCACCCTGGAG
MYL4	MYL4-2	XM_005257391.2	780—850	NW_002963147.1	208474-208544	H	GCTGAGATGGAGTCCTCGACTTATCACCACACCACTGCCCCAAGGACCTTACAGGCCCTCCCTGTTAATAA
						O	GCTGAGATGGAGTCCTCGACTTATCACCACACCACTGCCCCAAGGAGCTTACAGGCCCTCCCTGTTAATAA
NCOR1	NCOR1-1	XM_006721605.1	2579—2652	NC_012608.1	16438909-16438836	H	CACCCAGTACATCTCCCTCCTTAGCAGTTCCAAGTACAAAACCAGCTGAAGATGAAAGTGTGGAGACCCAGGTG
						O	CACCCAGTACATCTCCCTCCTCAGCAGTTCCAAGTACAAAACCAGCTGAAGATGAAAGTGTGGAGACCCAGGTG
	NCOR1-2	XM_006721605.1	2889—2938	NC_012608.1	16438599-16438550	H	TCAGCAAATAAATGCCCAAAGGCCCGAGCCCCAGTCAGACAATGATTCCA
						O	TCAGCAAATAAATGCCCAAAGGCCCGAGACCCAGTCAGACAATGATTCCA
NCOR2	NCOR2-3	NM_001206654.1	1984—2043	NC_012603.1	126754803-126754744	H	GAGGACAACGACGAGAAGGAGGCTGTGGCCTCCAAAGGCCGCAAAACTGCCAACAGCCAG
						O	GAGGACAACGACGAGAAGGAGGCCGTGGCCTCCAAAGGCCGCAAAACTGCCAACAGCCAG
	NCOR2-6	NM_001206654.1	7060—7134	NC_012603.1	126686015-126685941	H	AAGTCTCCAGGCAACACCAGCCAGCCGCCAGCCTTCTTCAGCAAGCTGACCGAGAGCAACTCCGCCATGGTCAAG
						O	AAGTCTCCAGGCAACACCAGCCAGCCGCCAGCCTTCTTCAGCAAGCTGACTGAGAGCAACTCCGCCATGGTCAAG
NEUROG1	NEUROG1-2	NM_006161.2	280—355	NC_012596.1	137039405-137039330	H	CTGCATCTCCGACCTCGACTGCGCCAGCAGCAGCGGCAGTGACCTATCCGGCTTCCTCACCGACGAGGAAGACTGT
						O	CTGCATCTCCGACCTCGACTGCGCCAGCAGCAGCGGCAGTGACCTGTCCGGCTTCCTCACCGACGAGGAAGACTGT
	NEUROG1-3	NM_006161.2	227—292	NC_012596.1	137039458-137039393	H	TCTATCTGTCCGTCGGTCCTGCACAGCGCAACGATGCCAGCCCGCCTTGAGACCTGCATCTCCGAC
						O	TCTATCTGTCCGTCGGTCCTGCACAGCGCAACGATGCCAGCCCCCCTTGAGACCTGCATCTCCGAC
NKX2-2	NKX2-2-1	NM_002509.3	360—416	NC_012611.1	16611462-16611406	H	GTCGCTGACCAACACAAAGACGGGGTTTTCGGTCAAGGACATCTTAGACCTGCCGGA
						O	GTCGCTGACCAACACAAAGACGGGGTTTTCCGTCAAGGACATCTTAGACCTGCCGGA
	NKX2-2-2	XM_006723566.1	837—905	NC_012611.1	16609513-16609445	H	CATGCCTCTCCTTCTGAACCTTGGGAGAGGGCTGAACTCTACGCCGTGTTTACAGAATGTTTGCGCAGC
						O	CATGCCTCTCCTTCTGAACCTTTGGAGAGGGCTGAACTCTACGCCGTGTTTACAGAATGTTTGCGCAGC
NODAL	NODAL-1	NM_018055.4	607—686	NC_012601.1	64846973-64847052	H	GCCACCAATGTGCTCCTTATGCTCTACTCCAACCTCTCGCAGGAGCAGAGGCAGCTGGGTGGGTCCACCTTGCTGTGGGA
						O	GCCACCAATGTGCTCCTTATGCTCTACTCCAACCTCTCACAAGAGCAGAGGCGGCTGGGTGGGTCCACCTTGCTGTGGGA
	NODAL-3	NM_018055.4	1943—2000	NC_012601.1	64850506-64850563	H	ATGGTCACAGCTCAGCCACTGGAAGCTGTGCGACCTCAGGTGAGCAATTCACTGTCCA
						O	ATGGTCACAGCTCAGCCACTGGAAGCTGTGTGACCCCAGGTGAGCAATTCACTGTCCA
NSD1	NSD1-2	XM_005265962.2	2556—2630	NC_012596.1	179981286-179981360	H	AATCCCAATCCTTGGTTTCCAGCCAGAGGCCACTGGACAGGCCACCAGCAGTGGCAGGACCAAGACCCCAGCTAA
						O	AATCCCAATCCTTGGTTTCCAGCCAGAGGCCACTGGACAGGCCACCAGCAGTGACAGGACCAAGACCCCAGCTAA
OGT	OGT-1	XM_006724714.1	509—568	NC_012614.1	69065652-69065711	H	GCTTATTCGAATTTGGGGAATGTGTACAAGGAAAGAGGGCAGTTGCAGGAGGCAATTGAG
						O	GCTTATTCGAATTTGGGGAATGTATACAAGGAAAGAGGGCAGTTGCAGGAGGCAATTGAG

	OGT-2	XM_006724714.1	2670—2749	NC_012614.1	69093878-69093957	H	AGTACGGGTTACCAGAAGATGCCATCGTATACTGTAACTTTAATCAGTTGTATAAAATTGACCCTTCTACTTTGCAGATG
						O	AGTACGGGTTACCAGAAGATGCCATTGTATACTGTAACTTTAATCAGTTGTATAAAATTGACCCTTCTACTTTGCAGATG
OLIG2	OLIG2-1	XM_005260908.1	2489—2557	NC_012612.1	33989367-33989435	H	CACGTGCTTCCTGCGTTCCGTGCAAGCCGCCTCGGCGCTGCCTGCGTTGCAAACTGGGCTTTGTAGCGT
						O	CACGTGCTTCCTGCGTTCCGTGCAAGCCACCTCGGCGCTGCCTGCGTTGCAAACTGGGCTTTGTAGCGT
	OLIG2-2	XM_005260908.1	1981—2033	NC_012612.1	33988858-33988910	H	GGGTCAATCCACACCCTCTTAGAACTGTGGCCGTTCCTCCCTGTCTCTCGTTG
						O	GGGTCAATCCACACCCTCTTAGAGCTGTGGCCGTTCCTCCCTGTCTCTCGTTG
ONECUT1	ONECUT1-2	NR_073510.1	782—850	NC_012606.1	49426491-49426423	H	CGACTTGGCAAGACAAATGATGAGCAGGAAAACACCACTGGATCTCACACCTTCAATCCATGACCATCC
						O	CGACTTGGCAAGACAAATGATGAGCAAGAAAACACCACTGGATCTCACACCTTCAATCCATGACCATCC
	ONECUT1-3	NR_073510.1	485—552	NC_012606.1	49426788-49426721	H	TGAACGCAAGAAGGAGGAGTCTGGACAAGTGGCAGGACGAGGGCAGCTCCAATTCAGGCAACTCATCT
						O	TGAACGCAAGAAGGAGGAGTCTGGACAAATGGCAGGATGAGGGCAGCTCCAATTCAGGCAACTCATCT
OTX1	OTX1-2	NR_130153.1	2766—2829	NC_012592.1	47797608-47797545	H	GCACCTTCGTTCTCCGAAATCTGCGGAGAGCCCGCGCGCCTGTGTATCAATTTTGGCTTTGGCC
						O	GCACCTTCGTTCTCCGAAATATGCGGAGAGCCCGCGCGCCTGTGTATCAATTTTGGCTTTGGCC
	OTX2-5	NR_073036.1	568—647	NC_012605.1	57359578-57359499	H	GCAGAGGTCCTATCCCATGACCTATACTCAGGCTTCAGGTTATAGTCAAGGATATGCTGGCTCAACTTCCTACTTTGGGG
						O	GCAGAGGTCCTATCCCATGACCTACACTCAGGCTTCAGGTTATAGTCAAGGATATGCTGGCTCAACTTCCTACTTTGGGG
PADI4	PADI4-3	NM_012387.2	873—936	NC_012591.1	213093198-213093135	H	CTGTGGTGTTCCAAGACAGCGTGGTCTTCCGCGTGGCGCCCTGGATCATGACCCCCAACACCCA
						O	CTGTGGTGTTCCAAGACAGCGTGGTCTTCCGCGTGGCACCCTGGATCATGACCCCCAACACCCA
PARP1	PARP1-1	NM_001618.3	3205—3268	NC_012591.1	23283934-23283997	H	TCCCTGTGGTAATTGGGAGAGGTAGCCGAGTCACACCCGGTGGCTCTGGTATGAATTCACCCGA
						O	TCCCTGTGGTAATTGGGAGAGGTGGCCGAGTCACACCCGGTGGCTCTGGTATGAATTCACCCGA
	PARP1-3	NM_001618.3	1993—2063	NC_012591.1	23268090-23268160	H	GAGGATGCCATTGAGCACTTCATGAAATTATATGAAGAAAAAACCGGGAACGCTTGGCACTCCAAAAATTT
						O	GAGGATGCCATTGAGCACTTCATGAAATTATATGAAGAAAAAACCGGGAATGCTTGGCACTCCAAAAATTT
PARP11	PARP11-1	XM_005253714.2	401—470	NC_012603.1	3900047-3899978	H	AAAAAGAGGTGTGCCTCAGATTAATGAACAAATGCTGTTTCATGGTACCAGCAGTGAATTTGTGGAAGCA
						O	AAAAAGAGGTGTGCCTCAGATTAATGAACAAATGCTATTTCATGGTACCAGCAGTGAATTTGTGGAAGCA
	PARP11-3	XM_005253714.2	2969—3043	NC_012603.1	3895668-3895594	H	TGTAATTGTGCAGTGGGAGGTAAGTAGTACCCAGGACATTGCATGTACAATACTTTGAAACAAAGTGGCAACAAA
						O	TGTAATTGTGCAGTGGGAGGTAAGTAGTACCCAGGACATTGCACGTACAATACTTTGAAACAAAGTGGCAACAAA
PARP12	PARP12-3	NR_130117.1	1866—1940	NC_012598.1	137280010-137279936	H	TGTGCTCTGAGTCAGCCAGTACCTTTCACTCTCATTGTCTGAACTTTAACGCCATGACTTACGGTGCTACCCAGG
						O	TGTGCTCTGAGTCAGCCAGTACCTTTCACTCTGATTGTCTGAACTTTAACGCCATGACTTACGGTGCTACCCAGG
	PARP12-5	NR_130117.1	2514—2588	NC_012598.1	137262857-137262783	H	GAGCTACTTTGCCCGAGATGCTGCATATTCCCACCACTACAGCAAATCCGACACGCAGACCCACACGATGTTCCT
						O	GAGCTACTTTGCCCGAGATGCTGCATATTCCCACCACTACAGCAAATCTGACACGCAGACCCACACGATGTTCCT
PARP14	PARP14-4	XR_427368.1	6355—6431	NC_012594.1	9955466-9955390	H	TGGCCAGACAATGAATGAGAAGCAACTCTTCCATGGGACAGATGCCGGCTCCGTGCCACACGTCAATCGAAATGGCT
						O	TGGCCAGACAATGAATGAGAAGCAACTCTTCCATGGGACAGATGCCAGCTCCGTGCCACACGTCAATCGAAATGGCT
PARP15	PARP15-1	XM_006713518.1	1216—1288	NC_012594.1	10064239-10064167	H	CATCAGCTGTTTTGCATGGTCCAGCTAGAGCCAGGACAATCAGAATATAATACCATAAAGGACAAGTTCACCC
						O	CATCAGCTGTTTTGCATGGTCCAGCTAGACCCAGGACAATCAGAATATAATACCATAAAGGACAAGTTCACCC
PARP16	PARP16-1	XM_006720592.1	1155—1222	NC_012606.1	62371096-62371029	H	TCGTGGTCACCAATAACCAGCTGCTGCGAGTGAAGTACCTCCTGGTGTATTCACAGAAGCCACCCAAG
						O	TCGTGGTCACCAATAACCAGCTCCTGCGAGTGAAGTACCTCCTGGTGTATTCACAGAAGCCACCCAAG
	PARP16-3	XM_006720592.1	789—863	NC_012606.1	62376876-62376802	H	CAGCCAACGCCAAATTTTATGAGACCAAAGGAGAACGAGACCTAATCTATGCATTTCATGGTAGCCGCCTAGAAA
						O	CAGCCAACGCCAAATTTTATGAGACCAAAGGAGAACGAGACCTAATCTATGCCTTTCATGGTAGCCGCCTAGAAA
PARP2	PARP2-1	XM_005267247.1	106—182	NC_012605.1	19654752-19654828	H	CAACACGGCTCCAGAAGACTCTTCCCCTGCCAAGAAAACTCGTAGATGCCAGAGACAGGAGTCGAAAAAGATGCCTG
						O	CAACACGGCTCCAGAAGACTCTTCCCCTGCCAAGAAAACTCGCAGATGCCAGAGACAGGAGTCGAAAAAGATGCCTG
	PARP2-3	NM_001042618.1	1495—1565	NC_012605.1	19666982-19667052	H	TCCTAAGGCCGAAGGATTGCTTCAAGGTAAACATAGCACCAAGGGGCTGGGCAAGATGGCTCCCAGTTCTG
						O	TCCTAAGGCCGAAGGATTGCTTCAAGGTAAACACAGCACCAAGGGGCTGGGCAAGATGGCTCCCAGTTCTG
PARP3	PARP3-3	NM_001003931.3	1769—1820	NC_012594.1	94412324-94412273	H	GACAACCCCAGCTTGAAGAGCCCACCTCCTGGCTTCGACAGTGTCATTGCCC
						O	GACAACCCCAGCTTGAAGAGCCCACCTCCCGGCTTCGACAGTGTCATTGCCC
PARP4	PARP4-1	NM_006437.3	1086—1158	NW_002952070.1	4845-4773	H	TGATACCTCACAAAGGCACAATGCCCAAAGAAGTGAACCTGGGACTATTGGCTAAGAAAGCAGACCTCTGCCA
						O	TGATACCTCACAAAGGCACAACGCCCAAAGAAGTGAACCTGGGACTATTGGCTAAGAAAGCAGACCTCTGCCA
PARP6	PARP6-1	XR_429467.1	64—143	NC_012606.1	69633573-69633494	H	AGTTGGCACTTCTGGCCTAACGCTGCCGTCATCCTACCCCTCACCCCAGGGCAACCCAGGCTGGACATTTAGTGCCTCCC
						O	AGTTGGCACTTCTGGCCTAACGCTGCCGTCATCCTACCCCTCGCCCCAGGGCAACCCAGGCTGGACATTTAGTGCCTCCC
	PARP6-4	XR_429467.1	333—408	NC_012606.1	69629964-69629889	H	TGTCTCCCTTTGTGGTCTCCTAAATGCCCATCTCGTTGGCCTTGGTTCGGCTAGTGGTATGGAGGGGTGCTGCCTA
						O	TGTCTCCCTTTGTGGTCTCCTAAATGCCCATCTCGTTGGCCTTGGTTCGGCTGGTGGTATGGAGGGGTGCTGCCTA
PARP8	PARP8-1	XM_005248597.2	1558—1607	NC_012596.1	48972663-48972712	H	GCGGATCCCTGTATTAAATGAATATTGTGTGGTTTGTGATGAGCCACATG
						O	GCGGATCCCTGTATTAAATGAATACTGTGTGGTTTGTGATGAGCCACATG
	PARP8-2	XM_005248597.2	944—1021	NC_012596.1	48970383-48970460	H	GGTTGTGGCAAAAGCAAATCCAAACTGAAATCTGAGCAGGACGGAATCTCCAAAACGCATAAGCTGCTGCGGAGGACT
						O	GGTTGTGGCAAAAGCAAATCCAAACTGAAATCTGAGCAGGACGGAATCTCCAAAACTCATAAGCTGCTGCGGAGGACT
PARP9	PARP9-1	XM_005247820.1	1269—1340	NC_012594.1	10141859-10141930	H	ACAGTTGGACCTGTGGCAAAGTCAATTCTACAACAAGCAGGAGTTGAAATGAAATCGGAATTTCTTGCCACA
						O	ACAGTTGGACCTGTGGCAAAATCAATTCTACAACAAGCAGGAGTTGAAATGAAATCGGAATTTCTTGCCACA
	PARP9-4	XM_005247820.1	877—956	NC_012594.1	10138465-10138544	H	TGCAGAGGGCCATTGTAAGTATTCTGAATTATGTCATCTATAAAAATACTCACATTAAGACAGTAGCAATTCCAGCCTTG

						O	TGCAGAGGGCCATTGTAAGTATTCTGAATTATGTCATCTATGAAAATACTCACATTAAGACAGTAGCAATTCCAGCCTTG
PAX7	PAX7-4	NM_001135254.1	3457—3531	NC_012591.1	211613041-211612967	H	TGAGGCAGATGTGACCAGTTTTCAAGCTACCAGCCCTGGGCAGAGGAAGATGTCAACAATTCCAGAGCAGAGGGA
						O	TGAGGCAGATGTGACCAGTTTTCAAGCTACCACCCCTGGGCAGAGGAAGATGTCAACAATTCCAGAGCAGAGGGA
PCGF1	PCGF1-1	XM_005264618.2	202—277	NC_012592.1	36193807-36193882	H	TTGTGAAGTACCTCCAAACTAGCAAGTACTGCCCCATGTGCAACATTAAGATCCACGAGACACAGCCACTGCTCAA
						O	TTGTGAAGTACCTCCAAACTAGCAAGTATTGCCCCATGTGCAACATTAAGATCCACGAGACACAGCCACTGCTCAA
PCGF2	PCGF2-1	NM_007144.2	564—624	NC_012608.1	50708466-50708526	H	CAACGGCTCCAATGAGGACCGCGGCGAGGTCTTGGAGCAGGAGAAGGGGGCTCTGAGTGAT
						O	CAACGGCTCCAATGAGGACCGAGGCGAGGTCTTGGAGCAGGAGAAGGGGGCTCTGAGTGAT
PCGF3	PCGF3-1	XM_006713852.1	2480—2538	NC_012595.1	752385-752443	H	AGGCAACACGGTTCTGAGTCACCTTCTGACACGAGCTCCCTCTGCTTGCTTTCCAGGTC
						O	AGGCAACACGGTTCTGAGTCATCTTCTGACACGAGCTCCCTCTGCTTGCTTTCCAGGTC
	PCGF3-2	XM_006713852.1	3497—3563	NC_012595.1	753394-753460	H	AGGAGCAAGGGCTGTTTTCTGGAGTGGTTGAGGTGTTGTCCTGCAGTTGTCATTGTCTTCTCCACCG
						O	AGGAGCAAGGGCTGTTTTCTGGAGCGGTTGAGGTGTTGTCCTGCAGTTGTCATTGTCTTCTCCACCG
PCGF5	PCGF5-1	XM_006718031.1	1380—1428	NC_012601.1	90009274-90009322	H	CTGTGCCGCCATGATTCACCCTTCTGAATATTTTGCTTGCCTGTTCCAA
						O	CTGTGCCGCCATGATTCAGCCTTCTGAATATTTTGCTTGCCTGTTCCAA
	PCGF5-2	XM_006718031.1	390—453	NC_012601.1	89952868-89952931	H	ATCTACTTAGGACCCCTCTTTGCCCAGACTACTAAAGCCAGTCTTCACTAGCCACGAATGGCTA
						O	ATCTACTTAGGACCCCTCTTTGCCCAAACTACTAAAGCCAGTCTTCACTAGCCACGAATGGCTA
PCYOX1	PCYOX1-2	NM_016297.3	1292—1365	NC_012592.1	40520496-40520423	H	CCCAAGAAACTCTTACTAAAGCACAAATTTTAAAGCTCTTTCTGTCCTATGATTATGCTGTGAAGAAGCCATGG
						O	CCCAAGAAACTCTTACTAAAGCACAAATTTTAAAGCTCTTTTTGTCCTATGATTATGCTGTGAAGAAGCCATGG
	PCYOX1-3	NM_016297.3	303—347	NC_012592.1	40535701-40535657	H	GGTGCAGGGGCAAGAATACGAGGCAGGAGGTTCTGTCATCCATCC
						O	GGTGCAGGGGCAAGAATATGAGGCAGGAGGTTCTGTCATCCATCC
PHC1	PHC1-2	NM_004426.2	4811—4870	NC_012603.1	8992002-8992061	H	GCATTTTGGCTGTATGTGGACAGATAAGACCACAGTCCTTAGCCCAATCCCAGCTATACA
						O	GCATTTTGGCTGTATGTGGACAGATAAGACCACAATCCTTAGCCCAATCCCAGCTATACA
	PHC1-4	XM_005253336.1	1782—1828	NC_012603.1	8984224-8984270	H	GTGGTAAAGGGTGGGGCTACCACCTCCTCACCTGTTGTAGCCCAGGT
						O	GTGGTAAAGGGTGGGGCTACCACGTCCTCACCTGTTGTAGCCCAGGT
PHC2	PHC2-1	XM_006710403.1	1126—1202	NC_012591.1	196880664-196880740	H	CATCAGCATGCTCAAGGACTCCTAGGGCTGGTGGCAGCCAGGATTCTGGCCCAGGGCGCCTCCTCCCGACTGAGCAG
						O	CATCAGCATGCTCAAGGACTCCTAGGGCTGGCGGCAGCCAGGATTCTGGCCCAGGGCGCCTCCTCCCGACTGAGCAG
	PHC2-2	XM_006710403.1	1749—1803	NC_012591.1	196881287-196881341	H	GGGAGCCTGGACTGACAGATAGGCCAAGGGCTACTCTCTGGCATCTCCAGGTGTT
						O	GGGAGCCTGGACTGACAGATAGGCCAACGGCTACTCTCTGGCATCTCCAGGTGTT
PHC3	PHC3-5	XM_006713757.1	1116—1178	NC_012594.1	173710597-173710535	H	ACTCCAGAACCATGGCCTTCCTCCAGCTCCCAGTAATGCCCAGTCACAGCATTGTTCACCGAT
						O	ACTCCAGAACCATGGCCTTCCTCCAGCTCCCAGTAGTGCCCAGTCACAGCATTGTTCACCGAT
	PHC3-6	XM_006713757.1	824—901	NC_012594.1	173717763-173717686	H	TCAGCCAACGAGATCCTTCTCCAGAAAGTAATAAGAAAGGAGAGAGCCCAAGCCTGGAATCACGAAGCACAGCTGTCA
						O	TCAGCCAACGAGATCCTTCTCCAGAAAGTAATAAGAAAGGAGAAAGCCCAAGCCTGGAATCACGAAGCACAGCTGTCA
PHF19	PHF19-2	XM_006717048.1	259—329	NC_012600.1	117705878-117705808	H	AACTGACGGAGGGCCAGTATGTGCTGTGCCGGTGGACAGATGGCCTGTACTACCTCGGGAAGATCAAGAGG
						O	AACTGACGGAGGGCCAGTATGTGCTGTGCCGGTGGACAGACGGCCTGTACTACCTCGGGAAGATCAAGAGG
	PHF19-3	XM_006717048.1	430—498	NC_012600.1	117701795-117701727	H	AGCCCAAGTGCAACATCTGCCTAGGGAAGACATCAGGGCCGCTGAATGAGATCCTCATCTGCGGGAAGT
						O	AGCCCAAGTGCAACATCTGCCTGGGGAAGACATCAGGGCCGCTGAATGAGATCCTCATCTGCGGGAAGT
PHF8	PHF8-2	XM_006724585.1	2255—2335	NC_012614.1	54384101-54384021	H	AGACAAGGCTAGGCTGATGGCAGAACAGGTGATGGAAGACGAATTTGACTTGGATTCAGATGATGAGCTGCAGATTGACGA
						O	AGACAAGGCTAGGCTGATGGCAGAACAGGTGATGGAAGACGAGTTTGACTTGGATTCAGATGATGAGCTGCAGATTGACGA
	PHF8-3	XM_006724585.1	773—858	NC_012614.1	54412998-54412913	H	GATTCTGAAGCCCACTGGAAATCAACTGACCGTGGAATTCCTGGAAGAAAATAGCTTCAGTGTGCCCATCCTGGTCCTGAAGAAGG
						O	GATTCTGAAGCCCACTGGAAATCAACTGACTGTGGAATTCCTGGAAGAAAATAGCTTCAGTGTGCCCATCCTGGTCCTGAAGAAGG
POLR2A	POLR2A-6	NM_000937.4	5732—5811	NC_012608.1	7530064-7530143	H	CTACAGCCCAACCTCTCCAAGCTACTCTCCAACATCACCCAGCTATTCCCCGACCTCACCAAGTTACTCCCCTTCCAGCC
						O	CTACAGCCCAACCTCTCCAAGCTACTCTCCAACATCACCCAGCTATTCCCCAACCTCACCAAGTTACTCCCCTTCCAGCC
POLR2B	POLR2B-1	NM_001303268.1	2975—3032	NC_012595.1	62963192-62963135	H	GGATTCCACAGATTGGAGACAAATTTGCTAGTCGACATGGTCAAAAGGGTACTTGTGG
						O	GGATTCCACAGATTGGAGACAAATTTGCCAGTCGACATGGTCAAAAGGGTACTTGTGG
	POLR2B-2	NM_001303268.1	1934—1989	NC_012595.1	62976910-62976855	H	CCGAACAACTTATGAACACCCTAAGGAAATTGAGACGTCAGATGGACATCATTGTG
						O	CCGAACAACTTATGAACACCCTAAGGAAATTGCGACGTCAGATGGACATCATTGTG
PRC1	PRC1-2	XM_006720760.1	2080—2140	NC_012606.1	87684798-87684738	H	GCCACATTGGGAGTCTGTTTGTTCCAATGGGTTGAGCTGTCTTTGTCGTGGAGATCTGGAA
						O	GCCACATTGGGAGTCTGTTTGTTCCAATGGGTTGGGCTGTCTTTGTCGTGGAGATCTGGAA
	PRC1-3	XM_006720760.1	162—223	NC_012606.1	87709069-87709008	H	TGGCGGAGGAGTCCATAGTATGTCTGCAGAAAGCCCTAAATCACCTTCGGGAAATATGGGAG
						O	TGGCGGAGGAGTCCATAGTATGTCTGCAGAAAGCGCTAAATCACCTTCGGGAAATATGGGAG
PRDM2	PRDM2-4	XM_006710880.1	5772—5831	NC_012591.1	216649995-216649936	H	GACTCCTGCTCTCTGGAGGACGGTCAGTCCATGTCTCGGAGAAACGGGTGAGCTGAGCTT
						O	GACTCCTGCTCTCTGGAGGACAGTCAGTCCATGTCTCGGAGAAACGGGTGAGCTGAGCTT
PRDM9	PRDM9-1	XM_005256274.2	1393—1448	NC_012596.1	24348459-24348514	H	GAACCAAAGCCAGAGATCCATCCATGTCCCTCATGCTGTCTGGCCTTTTCAAGTCA
						O	GAACCAAAGCCAGAGATCCATCCATGTCCCTCATGTTGTCTGGCCTTTTCAAGTCA
PRMT1	PRMT1-3	XM_005258842.1	1263—1338	NC_012610.1	51279594-51279669	H	AGGGGCTGAGCGTTCCTAGGCGGTTTCGGGGCTCCCCCTTCCTCTCCCTCCCTCCCGCAGAAGGGGGTTTTAGGGG
						O	AGGGGCTGAGCGTTCCTAGGTGGTTTCGGGGCTCCCCCTTCCTCTCCCTCCCTCCCGCAGAAGGGGGTTTTAGGGG
PRMT2	PRMT2-1	XM_006724000.1	1120—1180	NC_012612.1	48338950-48339010	H	TCGGTGAACATTCACTCCACATTGACCCCTCCCTAGCCTGGCAGGTGATGTCAGGGTCCTT
						O	TCGGTGAACATTCACTCCACACTGACCCCTCCCTAGCCTGGCAGGTGATGTCAGGGTCCTT

PRMT5	PRMT5-1	XR_429287.1	822—899	NC_012605.1	22391750-22391673	H	CAACCACCACTCAGAGAAGGAGTTCTGCTCCTACCTCCAATACCTGGAATACTTAAGCCAGAACCGTCCTCCACCTAA
						O	CAACCACCACTCAGAGAAGGAGTTCTGCTCCTACCTCCAGTACTTGGAATACTTAAGCCAGAACCGTCCTCCACCTAA
	PRMT5-2	XR_429287.1	1125—1202	NC_012605.1	22391099-22391022	H	CCTGGTGAACGCTTCCCTGCGGGCAGCCAAGCAGGCCGACCGGCGGATAAAGCTGTATGCTGTGGAGAAAAACCCAAA
						O	CCTGGTGAACGCTTCCCTGCGGGCAGCCAAGCAGGCTGACCGGCGGATAAAGCTGTATGCTGTGGAGAAAAACCCAAA
PRMT6	PRMT6-1	NM_018137.2	975—1045	NW_002901188.1	977-1047	H	GAGAAACCCCTGGTGCTGTCCACCTCGCCTTTTCACCCGGCCACTCACTGGAAACAGGCGCTCCTCTACCT
						O	GAGAAACCCCTGGTGCTGTCCACCTCGCCTTTTCACCCGGCCACGCACTGGAAACAGGCGCTCCTCTACCT
PRMT7	PRMT7-2	NM_001290018.1	1669—1732	NC_012607.1	55732194-55732257	H	CCGAGCTCAGGTGGTTCTCTCGTGGTGGGACATTGAAATGGACCCTGAGGGGAAGATCAAGTGC
						O	CCGAGCTCAGGTGGTTCTCTCATGGTGGGACATTGAAATGGACCCTGAGGGGAAGATCAAGTGC
	PRMT7-3	NM_001290018.1	1806—1881	NC_012607.1	55738111-55738186	H	ACTTCCTGCCACAAGAGGAGCCTGTGGTGCAGGGCTCAGCGCTCTATCTGGTAGCCCACCACGATGACTACTGCGT
						O	ACTTCCTGCCACAAGAGGAGCCTGTGGTGCAGGGCTCAGCGCTCTTTCTGGTAGCCCACCACGATGACTACTGCGT
PTEN	PTEN-1	NM_001304718.1	4944—5023	NC_012601.1	86471338-86471259	H	TTTGGATGTGCAGCAGCTTACATGTCTGAAGTTACTTGAAGGCATCACTTTTAAGAAAGCTTACAGTTGGGCCCTGTACC
						O	TTTGGATGTGCAGCAGCTTACATGTCTGAAGTTACTTGAAGGCGTCACTTTTAAGAAAGCTTACAGTTGGGCCCTGTACC
	PTEN-2	NM_001304718.1	5047—5153	NC_012601.1	86471235-86471129	H	CTTGAACATGTTTGCCATACTTTTAAAAGGGTAGTTGAATAAATAGCATCACCATTCTTTGCTGTGGCACAGGTTATAAACTTAAGTGGAGTTTACCGGCAGCATCA
						O	CTTGAACATGTTTGCCATACTTTTAAAAGGGTAGTTGAATAAATAGCGTCACCATTCTTTGCTGTGGCACAGGTTATAAACTTAAGTGGAGTTTACCGGCAGCATCA
RAD54L	RAD54L-1	XM_006710975.1	586—665	NC_012591.1	183631513-183631434	H	CATCATGGCTGATGAGATGGGCCTAGGAAAGACGCTGCAGTGCATCACATTGATGTGGACACTTTTACGCCAGAGTCCAG
						O	CATCATGGCTGATGAGATGGGCCTAGGAAAGACACTGCAGTGCATCACATTGATGTGGACACTTTTACGCCAGAGTCCAG
	RAD54L-2	XM_006710975.1	1985—2032	NC_012591.1	183614471-183614424	H	GTGGATGAGGAGCAGGATGTAGAGCGCCACTTCTCTCTGGGCGAGTTG
						O	GTGGATGAGGAGCAGGATGTAGAGCGTCACTTCTCTCTGGGCGAGTTG
RARB	RARB-2	NM_001290300.1	135—200	NC_012594.1	121822621-121822556	H	CCCCCATCTCCACTTCCTCCCCCTCGAGTGTACAAACCCTGCTTCGTCTGCCAGGACAAATCATCA
						O	CCCCCATCTCCACTTCCTCCCCCTCGAGTATACAAACCCTGCTTCGTCTGCCAGGACAAATCATCA
RB1	RB1-1	NM_000321.2	3247—3306	NC_012604.1	48655167-48655226	H	AATGGCCCTAGAGTGGGAGTCCTGATAACCCAGGCCTGTCTGACTACTTTGCCTTCTTTT
RB1						O	AATGGCCCTAGAGTGGGAGTCCTGATAACCCATGCCTGTCTGACTACTTTGCCTTCTTTT
RB1	RB1-2	NM_000321.2	2161—2221	NC_012604.1	48639601-48639661	H	TTGTGAACGCCTTCTGTCTGAGCACCCAGAATTAGAACATATCATCTGGACCCTTTTCCAG
RB1						O	TTGTGAACGCCTTCTGTCTGAGCACCCAGAACTAGAACACATCATCTGGACCCTTTTCCAG
RBBP4	RBBP4-1	NM_001135256.1	2555—2627	NC_012591.1	197540771-197540699	H	TCCAAGAGTGTCAAAGGCAGTGTGGTAGAGAGAATTTAAGGCAAGATTTAAATTTGGAAAAGGTGCTTGAACC
						O	TCCAAGAGTGTCAAAGGCAGTGTGGTAGAGAGAATTTAAGGCAAAATTTAAATTTGGAAAAGGTGCTTGAACC
	RBBP4-3	NM_001135256.1	5057—5103	NC_012591.1	197538254-197538208	H	GAGAATAGCAGGTGGGTAGGGTAGGATGAGGAAACAAGATGCCCAAA
						O	GAGAATAGCAGGTGGGTAGGGTAGGGTGAGGAAACAAGATGCCCAAA
RBBP7	RBBP7-1	NM_001198719.1	1014—1073	NC_012614.1	16805487-16805428	H	CTGGCCACTCAGCTGTTGTAGAGGATGTGGCCTGGCACCTGCTGCACGAGTCATTGTTTG
						O	CTGGCCACTCAGCTGTTGTAGAGGACGTGGCCTGGCACCTGCTGCACGAGTCATTGTTTG
	RBBP7-2	NM_001198719.1	1105—1176	NC_012614.1	16804564-16804493	H	ATGGGACACCAGGTCCAATACCACCTCCAAGCCGAGTCACTTGGTGGATGCGCACACTGCCGAAGTCAACTG
						O	ATGGGACACCAGGTCCAATACCACCTCCAAGCCGAGCCACTTGGTGGATGCGCACACTGCCGAAGTCAACTG
RBL2	RBL2-4	XM_005256083.2	957—1036	NC_012607.1	40553051-40553130	H	GATGCTGAGGAGGAAATTGGGACTCTCTCAAGGTGTCTGAACGCTGGTTCAGGAACAGAGACTGCTGAAAGGGTGCAGAT
						O	GATGCTGAGGAGGAAATTGGGACTCTCTCAAGGTGTCTGAATGCTGGTTCAGGAACAGAGACTGCTGAAAGGGTGCAGAT
REST	REST-2	XM_005265760.1	2249—2305	NC_012595.1	63058701-63058645	H	TTCCCCCTTCAGCAGTAGAAGAACGTGAAGCAGTGTCCAAAACTGCACTGGCATCAC
						O	TTCCCCCTTCAGCAGTAGAAGAACATGAAGCAGTGTCCAAAACTGCACTGGCATCAC
RING1	RING1-1	NM_002931.3	1043—1102	NW_002874576.1	1048821-1048880	H	TGTGAAGACAACTGGGAATGCCACAGTGGACCACCTCTCCAAGTACTTGGCCCTGCGCAT
						O	TGTGAAGACAACTGGGAATGCCACAGTGGACCATCTCTCCAAGTACTTGGCCCTGCGCAT
	RING1-2	NM_002931.3	467—541	NW_002874576.1	1047048-1047122	H	GGTGTCCAAGCGATCCCTACGGCCAGACCCCAACTTTGATGCCCTGATCTCTAAGATCTATCCTAGCCGGGAGGA
						O	GGTGTCCAAGCGATCCCTACGGCCAGACCCCAACTTCGATGCCCTGATCTCTAAGATCTATCCTAGCCGGGAGGA
RNF168	RNF168-1	NM_152617.3	1599—1660	NC_012594.1	200463723-200463662	H	CCAGAGTTCCCTACTCGAAAGAAACTGCAGTTATGCCTTGTGGCAGAACAGAAAGTGGGTGC
						O	CCAGAGTTCCCTACTCGAAAGAAACTGCAGTTATGCCTTGTGCCAGAACAGAAAGTGGGTGC
	RNF168-4	NM_152617.3	897—949	NC_012594.1	200483099-200483047	H	CTGATGACTATCAGCCAGTTCGTCTGCTCAGTAAACCTGGGGAACTGAGAAGA
						O	CTGATGACTATCAGCCAGTTCGTCTGTTAAGTAAACCTGGGGAACTGAGAAGA
RNF24	RNF24-1	XM_006723536.1	738—817	NC_012611.1	24363467-24363546	H	CAGTAAGCAGGACCGTGGACCCCCTCAGGGGCCCCTTCCTGGGGCAGAGAACATTGTATAGCTTACCGCAAGGATCAGAC
						O	CAGTAAGCAGGACCGTGGACCCCCTCAGGGGCCCCTTCCCGGGGCAGAGAACATTGTATAGCTTACCGCAAGGATCAGAC
	RNF24-2	XM_006723536.1	1080—1128	NC_012611.1	24363810-24363858	H	CTCTCTCCTGGAGCCTCATCTCCATGTCAAGCAGGAGGGTAAAGAAGGG
						O	CTCTCTCCTGGAGCCTCACCTCCATGTCAAGCAGGAGGGTAAAGAAGGG
RNF25	RNF25-3	NM_022453.2	1226—1284	NC_012593.1	110518345-110518287	H	AAGGAGCCCATGGACCTAAAGCCAGAACCCCATAGCCAAGGAGTTGAAGGTCCTCCACA
						O	AAGGAGCCCATGGACCTAAAACCAGAACCCCATAGCCAAGGAGTTGAAGGTCCTCCACA
RNF26	RNF26-1	NM_032015.4	627—694	NC_012602.1	116285170-116285237	H	GAGGCAGTGTACCTGGTAGTGAATGGGTTGGGCCTGGTGCTGGACGTGCTGACCTTGGTGTTGGACCT
						O	GAGGCAGTGTACCTGGTAGTGAATGGCGTGGGCCTGGTGCTGGACGTGCTGACCTTGGTGTTGGACCT
	RNF26-2	NM_032015.4	974—1040	NC_012602.1	116285517-116285583	H	GGGCGTCCTCAATGTGGTCTCCAGTGGCCATGCTTTGCTGCGCCAGGCCTGTGACATCTGTGCCATT
						O	GGGCGTCCTCAATGTGGTCTTCAATGGCCATGCTTTGCTGCGCCAGGCCTGTGACATCTGTGCCATT
RNF31	RNF31-2	NM_017999.4	1547—1608	NC_012605.1	23631282-23631343	H	ACCGGACTAGTAGCCCCATTCCAGCACAACATGCCCCCCGGCCCTATGCCAGCTCTTTGGAA
						O	ACCGGACTAGTAGCCCCATTCCATTACAACATGCCCCCCGGCCCTATGCCAGCTCTTTGGAA

RNF32	RNF32-1	XM_006715852.1	525—602	NC_012598.1	154802918-154802995	H	GCATGTCTTCAGGCTTTTGAAAAGTTCACAAATAAGAAAACCTGTCCTCTCTGTAGAAAGAACCAGTATCAAACCCGA
						O	GCATGTCTTCAGGCTTTTGAAAAGTTCACAAATAAGAAAACCTGCCCTCTCTGTAGAAAGAACCAGTATCAAACCCGA
	RNF32-2	XR_428168.1	10—74	NC_012598.1	154786908-154786972	H	AGGAAGGTGATAGGATGTGATGATAGAATTTGTGATAGCCAAGCAACAACTTTTCCTAATTCGGC
						O	AGGAAGGTGATAGGATGTGATGATAGAATTTGTGATGGCCAAGCAACAACTTTTCCTAATTCGGC
RNF34	RNF34-4	NM_001256858.1	330—391	NC_012603.1	123707829-123707890	H	CAAGCCTTGATGATGTGGAAGGAATGAGCGTGCGCCAGCTGAAGGAAATTCTGGCTCGGAAT
						O	CAAGCCTTGATGATGTGGAAGGGATGAGCGTGCGCCAGCTGAAGGAAATTCTGGCTCGGAAT
RNF38	RNF38-3	XM_006716721.1	539—609	NC_012600.1	25172174-25172244	H	CACCCTCCGAATGTATCTCCCCGTCTGCTACATCCTGCTGCTCATCCACCCCAGCAGAATGCAGTCATGGT
						O	CACCCTCCGAATGTATCTCCCCGTCTGTTACATCCTGCTGCTCATCCACCCCAGCAGAATGCAGTCATGGT
RNF40	RNF40-1	NM_001286572.2	3327—3403	NC_012607.1	29290435-29290511	H	ACCCTGGTGCATGCTAGTGGGCATGGGATCAGCCAAGCTTCGTTCCATCTTTTCCTAAAGGTCAGAGCTGCAGCCTA
						O	ACCCTGGTGCATGCTAGTGGGCGTGGGATCAGCCAAGCTTCGTTCCATCTTTTCCTAAAGGTCAGAGCTGCAGCCTA
	RNF40-6	NM_001286572.2	2657—2730	NC_012607.1	29285581-29285654	H	CTGACTGTGCAGAAGCTGGAGGAGAAGGAGCGAGCCTTGCAGGGCAGCCTCGGGGGTGTGGAGAAGGAGCTGAC
						O	CTGACTGTGCAGAAGCTGGAGGAGAAGGAACGGGCCTTGCAGGGCAGCCTCGGGGGTGTGGAGAAGGAGCTGAC
RNF8	RNF8-1	NR_046399.1	3420—3472	NC_012597.1	37467609-37467661	H	TGAAATGCCTCTCACCCTTTCCCCACAGCGACTACTTCCCACTCAGAGGCAAC
						O	TGAAATGCCTCTCACCCTTTCCCCACGGTGACTACTTCCCACTCAGAGGCAAC
	RNF8-4	XR_427857.1	1471—1540	NC_012597.1	37464982-37465051	H	CGCTGCTCTGAAGGTCAACTGAGAAGTCTTGTGGGACAGAGACTTGAGTTAGGAAGCCCTCAGTCACTTG
						O	GCTGCTCTGAAGGTCAACTGAGAGGTCTTGTGGGACAGAGACTTGAGTTAGGAAGCCCTCAGTCACTTG
RPIA	RPIA-1	NM_144563.2	1550—1608	NC_012592.1	22406052-22405994	H	GGAATGTAAGCTGTCAGGGAGAAAATGTTGTTACACTTTTGCTAAGATCTGGGGGTTTC
						O	GGAATGTAAGCTGTCAGGGAGAAAATGTGGTTACACTTTTGCTAAGATCTGGGGGTTTC
	RPIA-2	NM_144563.2	1737—1785	NC_012592.1	22405865-22405817	H	TCTGTTCCTTTGTTATTACTTGCATGGTTTGGCATCAGAAGTCCTTACC
						O	TCTGTTCCTTTGTTATTACTTGCATGATTTGGCATCAGAAGTCCTTACC
RPRD1A	RPRD1A-1	NM_001303411.1	1662—1712	NC_012609.1	48109423-48109373	H	TACCCTTCTCCCCAAACAAAATATCAATTCTGTGTCTCTACATGCCTTTGA
						O	TACCCTTCTCCCCAAACAAAATATCGATTCTGTGTCTCTACATGCCTTTGA
	RPRD1A-2	NM_001303413.1	385—452	NC_012609.1	48152075-48152008	H	TGTCATACAGAACAGCAAGAGGAAGGGGCCAGAGTTTACAAAAGATTTTGCACCAGTTATAGTGGAGG
						O	TGTCATACAGAACAGCAAGAGGAAGGGTCCAGAGTTTACAAAAGATTTTGCACCAGTTATAGTGGAGG
RYBP	RYBP-2	NM_012234.6	2518—2576	NC_012594.1	73268261-73268319	H	TTTGACCTTTAGGGACTCACGGGAGGGCAGCCTGATTTGTAATGAAGCACCACATTTTG
						O	TTTGACCTTTAGGGACTCACGGGAGGGCAGCCCAATTTGTAATGAAGCACCACATTTTG
	RYBP-3	NM_012234.6	544—603	NC_012594.1	73265893-73265952	H	CCTCCTAGTGAAGCAAACAGCATACAGTCTGCAAATGCTACAACAAAGACCAGCGAAACA
						O	CCTCCTAGTGAAGCAAACAGCATACAGTCTGCAAATGCCACAACAAAGACCAGCGAAACA
SAP130	SAP130-2	XM_006712749.1	3694—3746	NC_012593.1	6122520-6122572	H	TTCTTCTGCCTGACCTTCAAATGCCCATGTTGGCCTTTTACAGCAGTGCCACG
						O	TTCTTCTGCCTGACCTTCAAATGCCCACGTTGGCCTTTTACAGCAGTGCCACG
	SAP130-3	XM_006712749.1	1887—1949	NC_012593.1	6079864-6079926	H	CAACCCCAATCAACACACAAGGGCTTCAGCCTGCACCTATGGGTACTCAGCAGCCTCAGCCTG
						O	CAACCCCAATCAACACACAAGGGCTTCAGCCTGCACCAATGGGTACTCAGCAGCCTCAGCCTG
SAP18	SAP18-2	NM_005870.4	958—1002	NC_012604.1	20288350-20288394	H	GCTGTATGCAGTTGGAAAGGACACGTGAGAGTGAGCTCAAGTGGC
						O	GCTGTATGCAGTTGGAAAGGACATGTGAGAGTGAGCTCAAGTGGC
	SAP18-3	NM_005870.4	1242—1294	NC_012604.1	20288632-20288684	H	AGGGGTGAAGGAGGAATCTTAAGACGGAGGGAGAAGAGCCAAGGACAAGGTCA
						O	AGGGGTGAAGGAGGAATCTTAAGACTGAGGGAGAAGAGCCAAGGACAAGGTCA
SCMH1	SCMH1-3	XM_006710468.1	2815—2887	NC_012591.1	189052359-189052431	H	GGATCCCTGTCACCTTCTCCTCTCTTCACGGGGCCCTTTTGCAGCCCTAGGCCTCATCTGTGGGAAGGGAGTC
						O	GGATCCCTGTCACCTTCTCCTCTCTTCACGGGGCCGTTTTGCAGCCCTAGGCCTCATCTGTGGGAAGGGAGTC
	SCMH1-4	XM_006710470.1	425—490	NC_012591.1	188905872-188905937	H	ATGGGAGCGACAACAAAAATGACTTCTGGCGGCTGGTTGACTCAGCTGAAATCCAGCCTATTGGGA
						O	ATGGGAGCGACAACAAAAATGACTTCTGGCGGCTAGTTGACTCAGCTGAAATCCAGCCTATTGGGA
SENP2	SENP2-3	XM_005247691.1	70—148	NC_012594.1	189474446-189474524	H	GTTTGCGTCTCGGGGTGTGTCGGCCGCCGCTGCTGCTTGGGCCTGGTATGTACAGATGGCTGGTTAGGATTCTCGGCAC
						O	GTTTGCGTCTCGGGGTGTGTCGGCCGCCGCTGCTGCTCGGGCCTGGTATGTACAGATGGCTGGTTAGGATTCTCGGCAC
SETD1A	SETD1A-2	NM_014712.2	6089—6159	NC_012607.1	29521258-29521328	H	GTGCCGGCCTGTACAGATTCTGTCCTGGGGGGCTACACAGTCCTCTCGCTTTGTGTTAATGGGGACTTCCC
						O	GTGCCGGCCTGTACAGATTCTGTCCTGGGGGGCTGCACAGTCCTCTCGCTTTGTGTTAATGGGGACTTCCC
	SETD1A-3	NM_014712.2	1364—1439	NC_012607.1	29499386-29499461	H	CTACCCAGCAGGCACCACTGCGGTGGGCACTCCTGGCAACGGCACCCCCTGCTCCCAGGACACAAGCTTCTCCAGC
						O	CTACCCAGCAGGCACCACTGCAGTGGGCACTCCTGGCAACGGCACCCCCTGCTCCCAGGACACAAGCTTCTCCAGC
SETD1B	SETD1B-1	XM_006719296.1	6580—6635	NC_012603.1	124130736-124130791	H	GAGGAGACCCCACACCTTTCCCCACCCGAGCTGCAGCCTGTTCTTTCCCCAGAGGC
						O	GAGGAGACCCCACACCTTTCCCCACCCGAGCCCCAGCCTGTTCTTTCCCCAGAGGC
SETD2	SETD2-2	XM_006713120.1	504—581	NC_012594.1	99461228-99461305	H	GTAACATCCAGGCCACTGCTGGCTACTACCACAGCAGTAGCATCTCCACCTACTCATGCAGCACCATTACCAGCAGTG
						O	GTAACATCCAGGCCACTGCTGGCTACTACCACAGCAGTAGCATCTCTGCCTACTCATGCAGCACCATTACCAGCAGTG
	SETD2-4	XM_006713120.1	5628—5700	NC_012594.1	99528807-99528879	H	GCTGGTTACCCACCAGGTTATCCCATGCAGGCCTATGTGGATCCCAGCAACCCTAATGCTGGAAAGGTGCTCC
						O	GCTGGTTACCCACCAGGTTATCCCATGCAGGCTTATGTGGATCCCAGCAACCCTAATGCTGGAAAGGTGCTCC
SETD7	SETD7-1	NM_030648.2	1460—1515	NC_012595.1	144927527-144927472	H	GACAACCAGGGACTGCTCATGCTGTGACGTCACATCCTCTCACCATGCGTTAGCAA
						O	GACAACCAGGGACTGCTCATGCTGTGATGTCACATCCTCTCACCATGCGTTAGCAA
	SETD7-2	NM_030648.2	4204—4248	NC_012595.1	144924781-144924737	H	AGGACGGAAGGGTTTGTTTCCCGTTTGAACCTAAACGCAAGTGGA
						O	AGGACGGAAGGGTTTGTTTCCTGTTTGAACCTAAACGCAAGTGGA
SETD8	SETD8-1	XM_006719394.1	845—921	NC_012603.1	125780540-125780616	H	GCGTACCTCACCTCATCCTCATCGCCTCCCGAGACATCGCGGCTGGGGAGGAGCTCCTGTATGACTATGGGGACCGC
						O	GCGTACCTCACCTCATCCTCATTGCCTCCCGAGACATCGCGGCTGGGGAGGAGCTCCTGTATGACTATGGGGACCGC

	SETD8-2	XM_006719394.1	1243—1315	NC_012603.1	125780938-125781010	H	CATGCAGTCAAAGACTCAGCACAGGTTTTAGAGGAAATAGTCAAACATGAACTAGGAAGCCAGGTGAGTCTCC
						O	CATGCAGTCAAAGACTCAGCACAGGTTTTAGAGGAAAGAGTCAAACATGAACTAGGAAGCCAGGTGAGTCTCC
SETDB1	SETDB1-1	NM_001243491.1	1363—1442	NC_012591.1	100659863-100659784	H	CCATAGCCTTCCTCTTTCTTATCCTCGTATGTGTTCTCACTGTTTTTGGTCAAATTTTACTTTGCCCTTTTTGTTGTTCC
						O	CCATAGCCTTCCTCTTTCTTATCCTTGTATGTGTTCTCACTGTTTTTGGTCAAATTTTACTTTGCCCTTTTTGTTGTTCC
	SETDB1-2	XM_006711672.1	797—863	NC_012591.1	100662444-100662378	H	CGAATTCTGGGCAAGAAGAGAACTAAGACTTGGCACAAAGGCACCCTTATTGCCATCCAGACAGTTG
						O	CGAATTCTGGGCAAGAAGAGAACTAAGACTTGGCATAAAGGCACCCTTATTGCCATCCAGACAGTTGG
SETDB2	SETDB2-1	XM_005266572.2	2659—2728	NC_012604.1	49707448-49707517	H	AAAAAGGCAATTGAGGTTCAAATTCAGAAACCCCAAGAGGGACGATCTACAGCATGTCAAAGACAGCAGG
						O	AAAAAGGCAATTGAGGTTCAAATACAGAAACCCCAAGAGGGACGATCTACAGCATGTCAAAGACAGCAGG
	SETDB2-3	XM_006719880.1	2429—2508	NC_012604.1	49704633-49704712	H	CGGAGTCTGTCACTCCAGAAGATAATGATGGATTTAAACCACCCCGAGAGCATCTGAACTCTAAAACCAAGGGAGCACAA
						O	CGGAGTCTGTCACTCCAGAAGATAATGATGGATTTAAACCACTCCGAGAGCATCTGAACTCTAAAACCAAGGGAGCACAA
SETMAR	SETMAR-2	XM_006713297.1	507—557	NC_012594.1	65813968-65813918	H	TTGAATGCAATGTCCTGTGCCGATGCAGTGACCACTGCAGAAACAGAGTGG
						O	TTGAATGCAATGTCCTGTGCCAATGCAGTGACCACTGCAGAAACAGAGTGG
SFMBT1	SFMBT1-3	XM_006713204.1	2943—3015	NC_012594.1	93422142-93422214	H	TGAGCTGGATCTTTGAAGCACAAATGCAGCAAATCCTTCACCCTGCTTTATAAGTGGAGCTGGAATAGTCCTG
						O	TGAGCTGGATCTTTGAAGCACAAATGCAGCAAATCCTCCACCCTGCTTTATAAGTGGAGCTGGAATAGTCCTG
	SFMBT1-4	XM_006713204.1	2475—2550	NC_012594.1	93420012-93420087	H	AGTACATCCGAGCAGCAGGATGAGCTACAGGAAGAATCAGAAATGTCAGAAAAAAAGTCATGCTCCTCTTCTCCCA
						O	AGTACATCCGAGCAGCAGGATGAGCTGCAGGAAGAATCAGAAATGTCAGAAAAAAAGTCATGCTCCTCTTCTCCCA
SIN3A	SIN3A-3	XM_006720467.1	2136—2201	NC_012606.1	72871209-72871144	H	CGGGTTCTGGAAGCAATACAGAAGAAGCTTTCCCGCTTGTCTGCTGAAGAACAAGCCAAATTTCGC
						O	GGGTTCTGGAAGCAATACAGAAGAAGCTTTCCCGCTTATCTGCTGAAGAACAAGCCAAATTTCGC
SIN3B	SIN3B-1	NM_015260.3	3357—3435	NC_012610.1	17216713-17216791	H	AGGACAATGTGACGGTGGAGGCGGCTAGCCTGGTGCAGGACTGGCTGATGGGTGAGGAGGACGAGGACATGGTACCCTG
						O	AGGACAATGTGACGGTGGAGGCGGCCAGCCTGGTGCAGGACTGGCTGATGGGTGAGGAGGACGAGGACATGGTACCCTG
	SIN3B-4	NM_015260.3	1982—2047	NC_012610.1	17208358-17208423	H	CCGCACCTCATCTTTGTGTACGAGGACCGGCAGATCCTGGAGGACGCAGCAGCGCTCATCAGCTAC
						O	CCGCACCTCATCTTTGTGTATGAGGACCGGCAGATCCTGGAGGACGCAGCAGCGCTCATCAGCTAC
SIRT1	SIRT1-3	XM_006717737.1	630—709	NC_012601.1	67724977-67724898	H	GGCACAGATCCTCGAACAATTCTTAAAGATTTATTGCCGGAAACAATACCTCCACCTGAGTTGGATGATATGACACTGTG
						O	GGCACAGATCCTCGAACAATTCTTAAAGATTTATTGCCGGAAACAATTCCTCCACCTGAGTTGGATGATATGACACTGTG
SIRT2	SIRT2-2	XM_006723111.1	1399—1469	NC_012610.1	39782806-39782736	H	AGAGAGGGAGAAACCCCAGTGACAGCTGCATCTCCCAGGCGGGATGCCGAGCTCCTCAGGGACAGCTGAGC
						O	AGAGAGGGAGAAACCCCAGTGACAGCTGTATCTCCCAGGCGGGATGCCGAGCTCCTCAGGGACAGCTGAGC
	SIRT2-3	NM_001193286.1	80—134	NC_012610.1	39806208-39806154	H	ACGCCTTCTGGGACTCGTAGTCCGGTCCTCGCGCGCTTTCTTACCTAACTGGGGC
						O	ACGCCTTCTGGGACTCGTAGTCCCGTCCTCGCGCGCTTTCTTACCTAACTGGGGC
SIRT3	SIRT3-2	XM_005252835.1	1862—1941	NW_002981630.1	51492-51413	H	CATGAGTTGGAAAGGGAACCCTGGGATACGTGGCGTCCCCTCTATTGGAACAGTCTGAGGACTGAAGGCATTTGTCCCTG
						O	CATGAGTTGGAAAGGGAACCCTGGGATACATGGCGTCCCCTCTATTGGAATTGTCTGAGGACTGAAGGCATTTGTCCCTG
	SIRT3-4	XM_005252835.1	895—960	NW_002981630.1	59986-59921	H	GAAGCCCGACATTGTGTTCTTTGGGGAGCCGCTGCCCCAGAGGTTCTTGCTGCATGTGGTTGATTT
						O	GAAGCCCGACATTGTGTTCTTTGGTGAGCCGCTGCCCCAGAGGTTCTTGCTGCATGTGGTTGATTT
SIRT4	SIRT4-1	XM_006719312.1	275—350	NC_012603.1	122482453-122482528	H	CTTTATGCCCGCACTGACCGCAGGCCCATCCAGCATGGTGATTTTGTCCGGAGTGCCCCAATCCGCCAGCGGTACT
						O	CTTTATGCCCGCACTGACCGCAGGCCCATCCAGCATGGCGATTTTGTCCGGAGTGCCCCAATCCGCCAGCGGTACT
	SIRT4-3	XM_005253865.2	783—855	NC_012603.1	122492612-122492684	H	GGAGAGTTGCTGCCTTTGATAGACCCATGCTGACCACAGCCTGATATTCCAGAACCTGGAACAGGGACTTTCA
						O	GGAGAGTTGCTGCCTTTGATAGACCCACGCTGACCACAGCCTGATATTCCAGAACCTGGAACAGGGACTTTCA
SIRT5	SIRT5-1	XM_005248969.2	1076—1152	NC_012597.1	14117203-14117279	H	ACACGCAGAGGAGAAATGGTCTTATGGGTGGTGAGCTGAGTACTGAACAATCTAAAAATAGCCTCTGATTCCCTCGC
						O	ACACGCAGAGGAGAAATGGTCTTATGGGTGGTGAGCTGAATATTGAACAATCTAAAAATAGCCTCTGATTCCCTCGC
	SIRT5-2	XM_005248969.2	539—604	NC_012597.1	14097354-14097419	H	GAGGTCATGGGGAGCAAGGAGCCCAACGCCGGGCACCGCGCCATAGCCGAGTGTGAGACCCGGCTG
						O	GAGGTCATGGGGAGCAAGGAGCCCAACGCCGGGCACCGCGCCATAGCTGAGTGTGAGACCCGGCTG
SIRT6	SIRT6-1	XM_005259577.2	311—387	NC_012610.1	4149245-4149169	H	CCACGCAGACCCACATGGCGCTGGTGCAGCTGGAGCGCGTGGGCCTCCTCCGCTTCCTGGTCAGCCAGAACGTGGAC
						O	CCACGCAGACCCACATGGCGCTGGTGCAGCTGGAGCGCGTGGGCCTCCTCCACTTCCTGGTCAGCCAGAACGTGGAC
	SIRT6-2	XM_005259577.2	146—219	NC_012610.1	4150908-4150835	H	AACTGGCGAGGCTGGTCTGGCAGTCTTCCAATGTGGTGTTCCACACGGGTGCCGGCATCAGCACTGCCTCTGGC
						O	AACTGGCGAGGCTGGTCTGGCAGTCCTCCAATGTGGTGTTCCACACGGGTGCCGGCATCAGCACTGCCTCTGGC
SIRT7	SIRT7-2	XR_430032.1	307—345	NC_012608.1	72011756-72011718	H	CGTCCGGAACGCCAAATACTTGGTCGTCTACACAGGCGC
						O	GTCCGGAACGCCAAATACCTGGTCGTCTACACAGGCGC
SMARCAD1	SMARCAD1-1	XM_006714276.1	2386—2455	NC_012595.1	98335281-98335350	H	AAAAACATCAATGGGCCTGTGAAATAAGAACTGTGAACTCTCAATTGATGAGGAAATATCAACTTGGTGC
						O	AAAAACATCAATGGGCCTGTGAAATAAGAACTGTGAATTCTCAATTGATGAGGAAATATCAACTTGGTGC
	SMARCAD1-3	XM_006714277.1	211—283	NC_012595.1	98249974-98250046	H	TTCATTTAAAGCCCCCATCCCTGCAAGGTGGTGCTTTCTACCAATATGAATCTTTTCAACCTGGACCGTTTTC
						O	TTCATTTAAAGCCCCCATCCCTGCAAGGTGGTGGTTTCTACCAATATGAATCTTTTCAACCTGGACCGTTTTC
SMARCAL1	SMARCAL1-1	XM_006712557.1	508—575	NC_012593.1	108192715-108192782	H	GAGCCATGGTGTCATTTTCAAGCAACAGAATCTCAGTAGCTCATCTAATGCTGACCAAAGACCTCATG
						O	GAGCCATGGTGTCATTTTCAAACAACAGAATCTCAGTAGCTCATCTAATGCTGACCAAAGACCTCATG
	SMARCAL1-3	XM_006712557.1	1524—1605	NC_012593.1	108206532-108206613	H	CGTTTGCTTCTCAGCTCAAGAAGACATCTCTCAGTCTCACGCCAGATGTCCCAGAGGCAGACCTTTCTGAAGTGGACCCCAA
						O	CGTTTGCTTCTCAGCTCAAGAAGACATCTCTCAGTCTCACTCCAGATGTCCCAGAGGCAGACCTTTCTGAAGTGGACCCCAA
SMYD2	SMYD2-1	NM_020197.2	1062—1108	NC_012591.1	35575611-35575565	H	GTACCAGGCCATGGGTGTCTGCTTGTACATGCAGGACTGGGAAGGAG
						O	GTACCAGGCCATGGGTGTCTGTTTGTACATGCAGGACTGGGAAGGAG

	SMYD2-3	XR_426790.1	266—345	NC_012591.1	35592877-35592798	H	TGGAATGTTCTCCCATGGTTGTTTTTGGGGAAAACTGGAATCCCTCGGAGACTGTAAGACTAACAGCAAGGATTCTGGCC
						O	TGGAATGTTCTCCCATGGTTGTTTTTGGGGAAAACTGGAATCCCTCAGAGACTGTAAGACTAACAGCAAGGATTCTGGCC
SMYD3	SMYD3-6	XM_005273229.1	945—1003	NC_012591.1	3350623-3350681	H	TTTTTCCCAGGAAGCCATCCCGTCAGAGGGGTTCAAGTGATGAAAGTTGGCAAACTGCA
						O	TTTTTCCCAGGAAGCCATCCCGTCAGAGGCGTTCAAGTGATGAAAGTTGGCAAACTGCA
	SMYD3-7	XM_005273228.2	720—780	NC_012591.1	3179394-3179454	H	AGCTGTGACCCCAACTGTTCGATTGTGTTCAATGGGCCCCACCTCTTACTGCGAGCAGTCC
						O	AGCTGTGACCCCAACTGTTCGATTGTGTTTAATGGGCCCCACCTCTTACTGCGAGCAGTCC
SOX6	SOX6-1	NM_001145819.1	1208—1262	NC_012602.1	54048477-54048531	H	TGTCACCTGGAGCAAAGATGCCATCAACTCCACAGCCACCAAACACAGCAGGGAC
						O	TGTCACCTGGAGCAAAGATGCCATCAACTCCGCAGCCACCAAACACAGCAGGGAC
	SOX6-2	NM_001145819.1	693—752	NC_012602.1	53914987-53915046	H	AGCGCATGATGAACAGAAAAAACTGGCAGCGTCACAAATTGAGAAACAACGGCAGCAAAT
						O	AGCGCATGATGAACAGAAAAAACTGGCAGCCTCACAAATTGAGAAACAACGGCAGCAAAT
SSRP1	SSRP1-3	XM_005274194.1	1572—1650	NC_012602.1	19563217-19563295	H	GCATGAACCCAAGCTACGATGAATATGCTGACTCTGATGAGGACCAGCATGATGCCTACTTGGAGAGGATGAAGGAGGA
						O	GCATGAACCCAAGCTACGATGAATATGCCGACTCTGATGAGGACCAGCATGATGCCTACTTGGAGAGGATGAAGGAGGA
	SSRP1-4	XM_005274194.1	395—454	NC_012602.1	19559069-19559128	H	CAGGCTGGGGAGTTAACAGAAGGTATCTGGCGCCGTGTTGCTCTGGGCCATGGACTTAAA
						O	CAGGCTGGGGAGTTAACAGAAGGCATCTGGCGCCGTGTTGCTCTGGGCCATGGACTTAAA
STAG1	STAG1-1	XM_006713471.1	823—902	NC_012594.1	138964154-138964075	H	TTGGAGTCCTGATTCGACAGTGTCAGTATAGCATAATTTATGATGAGTATATGATGGACACAGTAATCTCCCTTTTGACG
						O	TTGGAGTCCTGATTCGACAGTGTCAGTATAGTATAATTTATGATGAGTATATGATGGACACAGTAATCTCCCTTTTGACG
	STAG1-3	XM_006713471.1	3462—3528	NC_012594.1	138791894-138791828	H	TGGCTAAACAGGACTGACACCATGATTCAGACTCCTGGCCCCCTGCCAGCACCACAACTCACATCCA
						O	TGGCTAAACAGGACTGACACCATGATTCAGACTCCTGGCCCCCTGGCAGCACCACAACTCACATCCA
STAT3	STAT3-2	XM_005257618.1	2099—2170	NC_012608.1	47127656-47127727	H	CAGGCACCTTCCTGCTAAGATTCAGTGAAAGCAGCAAAGAAGGAGGCGTCACTTTCACTTGGGTGGAGAAGG
						O	CAGGCACCTTCCTGCTAAGATTCAGTGAAAGCAGCAAAGAAGGCGGCGTCACTTTCACTTGGGTGGAGAAGG
	STAT3-4	XM_005257618.1	2289—2348	NC_012608.1	47128355-47128414	H	GGTGTCTCCGCTGGTCTATCTCTATCCTGACATTCCCAAGGAGGAGGCATTCGGAAAGTA
						O	GGTGTCTCCGCTGGTCTATCTCTACCCTGACATTCCCAAGGAGGAGGCATTCGGAAAGTA
SUDS3	SUDS3-1	NM_022491.2	2934—2995	NC_012603.1	120540530-120540591	H	TTCCATTGAAGGTGGAGTTTTTCAATGATCATGTGTTTTGTCCTCCTAAACAGTATACCAAA
						O	TTCCATTGAAGGTGGAGTTTTTCAATGATCATGTTTTTTGTCCTCCTAAACAGTATACCAAA
	SUDS3-3	NM_022491.2	3520—3567	NC_012603.1	120541122-120541169	H	TGGCCATTATTCCAAAAGGTCATTGTTTCTCACTAGACCCCAGGGCAC
						O	TGGCCATTATTCCAAAAGGTCTTTGTCTCTCACTAGACCCCAGGGCAC
SUV39H1	SUV39H1-2	NM_003173.3	1929—1980	NC_012614.1	49511042-49511093	H	ACTGGAAGGGAAGTGGGTGTCCATGGGCCACTGAGCAGTGAGAGGAAGGCAG
						O	ACTGGAAGGGAAGTGGGTGTCCATGGCCCACTGAGCAGTGAGAGGAAGGCAG
SUV39H2	SUV39H2-2	XM_006717503.1	1181—1256	NC_012601.1	15630805-15630880	H	TGACAACAAGGGAATCACGTATCTCTTTGATCTGGACTATGAGTCTGATGAATTCACAGTGGATGCGGCTCGATAC
						O	TGACAACAAGGGAATCACGTATCTCTTTGATCTGGACTACGAATCTGATGAATTCACAGTGGATGCGGCTCGATAC
SUV420H1	SUV420H1-2	NM_001300909.1	544—604	NC_012602.1	7941016-7941076	H	TCGGGATTTGAAGGACAGAGTCGCTATGTACCATCCTCTGGAATGTCCGCCAAGGAACTCT
						O	TCGGGATTTGAAGGACAGAGTCGCTATGTACCATCTTCTGGAATGTCCGCCAAGGAACTCT
SUZ12	SUZ12-2	XM_006721794.1	2824—2900	NC_012608.1	25464358-25464282	H	TGCAAGGATGTCTTTGTAATGTGTTTCATGAATAGAATATCCAATAGAGATAAGCTGACTTGAATCATTTTGAGCAA
						O	TGCAAGGATGTCTTTGTAATGTGTTTCATGAATAGAATATCCAATACAGATAAGCTGACTTGAATCATTTTGAGCAA
	SUZ12-3	XM_006721794.1	2961—3028	NC_012608.1	25464221-25464154	H	AAGTGGATTCACTACTGGCACATTAACAAGCACCAATAGGTTTTTATTCCAACTCCGAGCACTGTGGT
						O	AAGTGGATTCACTACTGGCACATTAACAAGCATCAATAGGTTTTTATTCCAACTCCGAGCACTGTGGT
TBX3	TBX3-2	NM_016569.3	975—1029	NC_012603.1	116655431-116655377	H	CCATGAGAGATCCGGTCATTCCTGGGACAAGCATGGCCTACCATCCGTTCCTACC
						O	CCATGAGAGATCCGGTCATTCCTGGGACAACCATGGCCTACCATCCGTTCCTACC
	TBX3-3	NM_016569.3	640—699	NC_012603.1	116655764-116655705	H	CTCCTCTCGCTTCTGAAACCGACGTTCAGGAGCGGCTTTTTAAAAACGCAAGGCACAAGG
						O	CTCCTCTCGCTTCTGAAACCGACTTTCAGGAGCGGCTTTTTAAAAACGCAAGGCACAAGG
TCF3	TCF3-1	XM_006722863.1	3419—3478	NC_012610.1	1556444-1556385	H	CATCCTCTCCCCTCAGGAAGGGGACTGACCTGGGCTTGGGGGAAGGGACGTCAGCAAGGT
						O	CATCCTCTCCCCTCAGGAAGGGGGCTGACCTGGGCTTGGGGGAAGGGACGTCAGCAAGGT
TET1	TET1-1	NM_030625.2	6023—6081	NC_012601.1	66893271-66893213	H	CCAGTGAAAGAGGCATCTCCAGGCTTCTCCTGGTCCCCGAAGACTGCTTCAGCCACACC
						O	CAGTGAAAGAGGCATCTCCAGGCTTCTCTTGGTCCCCGAAGACTGCTTCAGCCACACC
TET2	TET2-1	XM_006714243.1	342—419	NC_012595.1	109892315-109892392	H	GAAGGGAAGCCAGAATAGTCGTGTGAGTCCTGACTTTACACAAGAAAGTAGAGGGTATTCCAAGTGTTTGCAAAATGG
						O	GAAGGGAAGCCAGAATAGTCGTGTGAGTCCTGACTTTATACAAGAAAGTAGAGGGTATTCCAAGTGTTTGCAAAATGG
	TET2-3	XM_006714243.1	985—1057	NC_012595.1	109892958-109893030	H	AACTCTGAGCTGCCTCCAAAGCCAGCTGCAGTGGTGAGTGAGGCCTGTGATGCTGATGATGCTGATAATGCCA
						O	AACTCTGAGCTGCCTCCAAAGCCAGCTGCAGTGGTGACTGAGGCCTGTGATGCTGATGATGCTGATAATGCCA
TET3	TET3-1	XM_006711963.1	3810—3882	NC_012592.1	36602349-36602277	H	GATCTGGAGTGCGAGGTTCTTAGGGGCCGTGCCCACCATGTTGCCAAGCCAATGCATGCTGAGCTGAAGGAATTTG
						O	GATCTGGAGTGCGAGGTTCTTAGGGGCCGCGCCCACCATGTTGCCAAGCCAATGCATGCTGAGCTGAAGGAATTTG
	TET3-6	XM_006711963.1	36—112	NC_012592.1	36632435-36632359	H	GTGGAGAAAGATGAAGGTCCATATTATACTCACTTGGGATCTGGCCCCACGGTCGCCTCTATCCGGGAACTCATGGA
						O	GTGGAGAAAGATGAAGGTCCATATTATACTCACCTGGGATCTGGCCCCACGGTCGCCTCTATCCGGGAACTCATGGA
TK1	TK1-1	NM_003258.4	335—404	NC_012608.1	68228705-68228636	H	GCTTCCAGATTGCTCAGTACAAGTGCCTGGTGATCAAGTATGCCAAAGACACTCGCTACAGCAGCAGCTT
						O	GCTTCCAGATTGCTCAGTACAAGTGCCTGGTGATCAAGTACGCCAAAGACACTCGCTACAGCAGCAGCTT
	TK1-2	XM_005257631.1	1511—1580	NC_012608.1	68217802-68217733	H	TGATGGTTTCCACAGGAACAACAGCATCTTTCACCAAGATGGGTGGCACCAACCTTGCTGGGACTTGGAT
						O	TGATGGTTTCCACAGGAACAACAGCATCTTTCACCAACATGGGTGGCACCAACCTTGCTGGGACTTGGAT

TP53	TP53-2	NM_001126118.1	1594–1666	NC_012608.1	7677157-7677085	H	GCAATAGGTGTGCGTCAGAAGCACCCAGGACTTCCATTTGCTTTGTCCCGGGGCTCCACTGAACAAGTTGGCC
						O	GCAATAGGTGTGCGTCAGAAGCACCCAGGACTTCCGTTGGCTTTGTCCCGGGGCTCCACTGAACAAGTTGGCC
TP53BP1	TP53BP1-4	XM_005254634.2	1599–1677	NC_012606.1	39915168-39915090	H	TCCCCAAAGATGGAGAGCTTGAGTTCTCACAGAATTGATGAAGATGGAGAAAACACACAGATTGAGGATACGGAACCCA
						O	TCCCCAAAGATGGAGAGCTTGAGTTCTCACACAATTGATGAAGATGGAGAAAACACACAGATTGAGGATACGGAACCCA
	TP53BP1-7	XM_006720666.1	2680–2751	NC_012606.1	39867200-39867129	H	GGGGTATTTGATGTGGTGGTGACGGACCCCTCATGCCCAGCCTCGGTGCTGAAGTGTGCTGAAGCATTGCAG
						O	GGGGTATTTGATGTGGTGGTGACGGACCCCTCGTGCCCAGCCTCGGTGCTGAAGTGTGCTGAAGCATTGCAG
TRDMT1	TRDMT1-2	NM_004412.5	4541–4604	NC_012601.1	17916401-17916338	H	GAGGCTTTTAAGTTGCCCTGAAAGGCTGTATTGCTACCATGTGGGATGTAAAATTGGATGGGGT
						O	GAGGCTTTTAAGTTGCCCTGAGAGGCTGTATTGCTACCATGTGGGATGTAAAATTGGATGGGGT
	TRDMT1-4	NM_004412.5	6821–6900	NC_012601.1	17914175-17914096	H	CCACATCTTCCAAGTTCAAGCTTCATAACCAAAGATCATCTTCACCTTCCTTCTCACTCTGCATTCCATTGGTCAGCTTC
						O	CCACATCTTCCAAGTTCAAGCCTCATAACCAAAGATCATCTTCACCTTCCTTCTCACTCTGCATTCCATTGGTCAGCTTC
UHRF1	UHRF1-1	NM_001290052.1	3278–3322	NC_012610.1	4958189-4958233	H	GTCACGTGCAAGTGCCTTTGATTCGTTCCTTCTTTCTAAAGACGA
						O	GTCACGTGCAAGTGCCTTTGGTTCGTTCCTTCTTTCTAAAGACGA
	UHRF1-2	NM_001290052.1	774–823	NC_012610.1	4929031-4929080	H	CAAGTGGCAGGACCTGGAGGTGGGCCAGGTGGTCATGCTCAACTACAACC
						O	CAAGTGGCAGGACCTGGAGGTGGGCCAGGTAGTCATGCTCAACTACAACC
VPS72	VPS72-1	NM_005997.2	870–928	NC_012591.1	100423481-100423539	H	CGCTGCTCACGTACCTTCATCACTTTTAGTGATGATGCAACTTTCGAGGAATGGTTCCC
						O	CGCTGCTCACGTACCTTCATCACTTTTAGTGATGACGCAACTTTCGAGGAATGGTTCCC
	VPS72-2	NM_005997.2	906–980	NC_012591.1	100423517-100423591	H	GCAACTTTCGAGGAATGGTTCCCCCAAGGGCGGCCCCCAAAAGTCCCTGTTCGTGAGGTCTGTCCAGTGACCCAT
						O	GCAACTTTCGAGGAATGGTTCCCCCAAGGGCGGCCCCCAAAAGTCCCTGTTCGGGAGGTCTGTCCAGTGACCCAT
WDR5	WDR5-1	XM_005272163.1	1523–1596	NC_012600.1	131117406-131117479	H	AGTTCAATTTAACATGCGTTGTGTTTTTTCAGTAAACGTTCTGTATCTTTTTGATATTCCATGACCCAGTGCAC
						O	AGTTCAATTTAACATGCGTTGTGTCTTTTCAGTAAACGTTCTGTATCTTTTTGATATTCCATGACCCAGTGCAC
WHSC1	WHSC1-2	XM_006713915.1	2484–2547	NC_012595.1	1976120-1976183	H	GCCAGGACACAGACGTACAGGCCTCCTCGGGAGGGAGCGCCTCCCCACCACTGAGCCATCCTCA
						O	GCCAGGACACAGACGTACAGGCCTCCTTGGGAGGGAGCGCCTCCCCACCACTGAGCCATCCTCA
	WHSC1-3	XM_006713914.1	1446–1524	NC_012595.1	1914331-1914409	H	ACCCCGACATAGGGAAGAGTACTCCTCAAAAGACGGCAGAGGCTGACCCCAGAAGAGGAGTAGGGTCTCCTCCTGGGAG
						O	ACCCCGACATAGGGAAGAGTACTCCTCAAAAGACAGCAGAGGCTGACCCCAGAAGAGGAGTAGGGTCTCCTCCTGGGAG
WHSC1L1	WHSC1L1-1	XM_005273548.1	733–782	NC_012599.1	38629309-38629260	H	GGCCAGACACCATATGAAGCTACTTTGCAGCAAGGCTTTCAGTACCCAGC
						O	GGCCAGACACCATATGAAGCTACCTTGCAGCAAGGCTTTCAGTACCCAGC
	WHSC1L1-2	XM_005273548.1	948–1023	NC_012599.1	38629094-38629019	H	TCCAAACACAAGACCACATGAAATTCTGGAAAAACCTTCCCCTCCACAGCCACCACCTCCTCCTTCGGTACCACAA
						O	TCCAAACACAAGACCACATGAAATTCTGGAAAAAGCTTCCCCTCCACAGCCACCACCTCCTCCTTCGGTACCACAA
WNT8A	WNT8A-2	NM_058244.3	1229–1308	NC_012596.1	139654315-139654394	H	AGTCCCTGGGTAAGGGCAGTGCCTGATAATACCCCACACAAGTTCACTTGATTAATTGCATCAGTGGAAGGGGACATAGC
						O	AGTCCCTGGGTAAGGGCAGTGCCTGATGATACCCCACACAAGTTCACTTGATTAATTGCATCAGTGGAAGGGGACATAGC
YAF2	YAF2-2	XM_006719185.1	493–545	NC_012603.1	41519660-41519608	H	GCCTGCATCTAGTGCTGCTTCTGCAGATCAACACAGTCAAAGCGGCTCTAGCT
						O	GCCTGCATCTAGTGCTGCTTCTGCAGATCCACACAGTCAAAGCGGCTCTAGCT
	YAF2-3	XM_006719185.1	684–741	NC_012603.1	41519469-41519412	H	TGCCAAGAGGTACCACATTTTCATGACAGATACATTCATGCACAATCCATAATTTGAG
						O	TGCCAAGAGGTACCACATTTTCATGACAAATACATTCATGCACAATCCATAATTTGAG
	YAF2-5	XR_245886.2	610–652	NC_012603.1	41520703-41520661	H	CTCGACCTGTCTCCCAGTTGGTTGCACAGCAGGTTACTCAGCA
						O	CTCGACCTGTCTCCCAGTTGGTCGCACAGCAGGTTACTCAGCA
YY1	YY1-2	NM_003403.4	2681–2757	NC_012605.1	101797374-101797450	H	TGGAAGCTAAATAAAGCAACTCAAGTTTCCTTTATTTTGCACTCAATTACAGTGATTATTGATGAAAGCGATGCATG
						O	TGGAAGCTAAATAAAGCAACTCAAATTTCCTTTATTTTGCACTCAATTACAGTGATTATTGATGAAAGCGATGCATG
ZNF317	ZNF317-2	XR_430146.1	24–98	NC_012610.1	9321227-9321301	H	GCGGAAGCCATTACTCTCTGGAGTCGATTGCCCCGAGACACATGGGCCAAGGAGGGGTCAGCGGCGAATTCTTTC
						O	GCGGAAGCCATTACTCTCTGGAGTCGGTTGCCCCGAGACACATGGGCCAAGGAGGGGTCAGCGGCGAATTCTTTC
	ZNF317-3	XR_430146.1	405–462	NC_012610.1	9338467-9338524	H	CAGAATTTGGACCTGTTCGTGTGCAGTGGTCTGGAGCCTCACACACCCAGTGTTGGTT
						O	CAGAATTTGGACCTGTTCGCGTGCAGTGGTCTGGAGCCTCACACACCCAGTGTTGGTT
ARF6	ARF6-3	NM_001663.3	1205–1266	NC_012605.1	50320879-50320940	H	ATTATCGCCACCATCACCTCTTTCAATTGCCACTTTCTCTTCTTTTGAATTTGAACTCTGGA
						O	ATTATCGCCACCATCACCTCTTTGAATCGCCACTTTCTCTTCTTTTGAATTTGAACTCTGGA
						O	ATTATCGCCACCATCACCTCTTTCAATCGCCACTTTCTCTTCTTTTGAATTTGAACTCTGGA
HDAC1	HDAC1-3	NM_004964.2	821–886	NC_012591.1	197901557-197901492	H	AGCCTAGTGCGGTGGTCTTACAGTGTGGCTCAGACTCCCTATCTGGGGATCGGTTAGGTTGCTTCA
						O	AGCCTAGTGCGGTGGTCTTACAGTGTGGCTCCGACTCCCTATCTGGGGATCGGTTAGGTTGCTTCA
KDM6A	KDM6A-2	NR_111960.1	2742–2809	NC_012614.1	45723329-45723396	H	GCCTCTTCACCATCTTCAGCCATTTCAACAGCAACACCTTCTCCAAAATCCACTGAGCAGACAACCAC
						O	GCCTCTTCACCATCTTCAGCCATTTCCACAGCAACACCTTCTCCAAAATCCACTGAGCAGACAACCAC
NANOG	NANOG-5	NM_001297698.1	1680–1778	NC_012603.1	8147866-8147966	H	GTTTGGATATCTTTAGGGTTTAGAATCTAACCTCAAGAATAAGAAATACAAGTACAAATTGGTGATGAAGATGTATTCGTATTGTTTGGGATTGGGAGG
						O	GTTTGGATATCTTTAGGGTTTAGAATCTAACCTCAAGAATAAGAAATACAAGTACAAATTGGTGTGATGAAGATATATTCCTATTGTTTGGGATTGGGAGG
RARB	RARB-3	NM_001290300.1	2103–2182	NC_012594.1	121678296-121678217	H	GATAGCCTCCAAGGCAGAAACACTTTTCAGTGTTAAGTTTTTGTTTACTTGTTCACAAGCCATTAGGGAAATTTCATGGG
						O	GATAGCCTCCAAGGCAGAAACACTTTTCAGTGTTAAGTTTTTGTTTACCTGTTCACAAGCCATTAGGGAAATTTCATGG
RNF2	RNF2-5	XM_005245413.1	1313–1375	NC_012591.1	65493841-65493779	H	TTTTGACCCAGGAATTTTTCATAGTTTCTGTATTCTTATAAGATTCAGTTGGCTGTCCTTTTC
						O	TTTTGACCCAGGAATTTTTCATAGTTTGTGTATTCTTATAAGATTCAGTTGGCTGTCCTTTTCC
SAP25	SAP25-1	XM_006715822.1	919–990	NC_012598.1	9633477-9633406	H	GACACCCATTGTCCATAGCCTTCTCAGGGCAGAGTGGGCTGGTTGTGTTGACAATAAAACAGTGTTGGTTTG

						O	GACACCCATTGTCCATAGCCTTCTCGGTGCAGAGTGGGCTGGTTATGTTGACAATAAAACAGTGTTGGTTTG
SOX2	SOX2-3	NM_003106.3	1047–1122	NC_012594.1	185447028-185447103	H	GCCCTGCAGTACAACTCCATGACCAGCTCGCAGACCTACATGAACGGCTCGCCCACCTACAGCATGTCCTACTCGC
						O	GCCCTGCAGTACAACTCCATGACCAGCTCGCAGACCTACATGAACGGCTCGCCAACCTACAGCATGTCCTACTCGC

Ori: origin of amplicon sequence, H, human and O, orangutan

Supplementary Table 4

Amplicon	REP1																									
	G10		G9		G7		G5		G3		G1		G0		I1		I2		I3		F1		F2		F3	
	HS	PA	HS	PA	HS	PA	HS	PA	HS	PA	HS	PA	HS	PA	HS	PA	HS	PA	HS	PA	HS	PA	HS	PA	HS	PA
ACTL6A-1	2902	8	1861	124	1088	220	1013	623	472	733	246	1467	11	2460	9193	519	6309	673	2534	693	3854	825	1044	755	614	1060
ACTL6A-2	2385	5	2194	191	1139	323	858	627	468	695	171	1427	4	1189	1585	663	1107	866	623	1211	1377	1265	584	1219	292	1408
AEBP2-1	12381	22	9510	432	4994	1337	4127	3421	1919	4200	562	7889	13	12924	8736	1773	6241	3189	2401	3726	3770	5192	1094	4282	643	6235
ARF3-3	2036	120	1600	212	781	142	906	285	655	917	327	2123	86	2887	2032	333	1601	594	943	902	5557	1223	1877	1249	904	1383
ARF4-4	8764	0	7282	63	3422	319	2880	750	1605	1233	406	2421	67	4467	31549	150	33080	330	18056	588	43114	157	27104	425	26067	1019
ARF5-1	11222	50	12307	941	6088	1902	3966	3494	2291	5038	1003	9182	76	9889	9234	368	10467	926	10066	1863	12134	913	11211	1983	6285	2731
ARF5-2	3699	16	3180	294	1667	850	1509	1844	845	2367	403	4437	20	4790	14627	470	11916	776	5800	845	12512	1003	4449	934	3653	1830
ARF6-3	1897	8	1590	218	885	455	970	1190	590	1718	443	3407	119	4561	24998	858	16967	1379	6287	1311	31285	1343	10739	1285	7412	2489
ARID4B-1	2052	8	1949	127	899	278	827	497	412	685	188	1508	4	1525	1646	311	1289	418	607	571	2518	669	872	680	399	734
ARID4B-2	7684	105	5949	531	3854	814	2993	1642	2279	2355	883	6147	198	7339	6124	775	4966	1394	2010	1525	8270	1988	2595	1719	1495	2584
ASF1A-1	229	0	168	20	100	66	67	83	20	109	6	217	0	488	220	107	154	128	38	129	132	133	31	149	7	182
ASF1A-2	1182	5	870	66	571	229	426	533	225	680	103	1173	5	1421	1422	507	934	735	269	636	881	958	234	648	144	728
ASH1L-4	1035	12	758	89	405	187	392	395	245	577	81	880	4	1347	581	391	339	454	78	392	998	492	253	340	111	426
ASH1L-5	4456	13	3430	295	2111	827	1692	1660	806	1789	327	3575	9	5448	1561	647	1317	1135	740	1385	4025	1428	1481	1280	1182	2330
ASXL1-2	29610	113	24523	2045	12262	4590	9533	9625	5719	15799	2109	27994	150	28107	4999	10309	3525	15406	1356	15234	13832	11835	5039	12951	2949	17722
ASXL1-3	7030	17	6099	624	3423	1232	2671	2336	1619	3310	746	6682	4	7647	1928	3117	1364	4226	600	4317	4772	3548	2064	3999	888	4727
AXIN2-1	4635	20	4683	406	2073	660	1703	1225	882	1639	413	3026	31	4833	2500	1915	1575	2209	549	2200	1306	2507	465	2338	153	2147
BAP1-3	12832	88	9760	1652	4396	3566	3441	6212	1376	6896	607	10946	32	11594	6674	6416	4913	9073	1815	8145	5781	9510	1659	8190	1016	10038
BMI1-1	234	18	161	38	47	18	79	31	149	192	72	477	14	632	249	80	184	155	105	264	4810	319	1922	338	835	359
BMI1-2	7793	11	7035	276	3529	675	2093	1526	1293	2823	475	4633	40	4537	2781	880	1955	1633	812	1943	13483	885	10133	1803	4889	2019
BRCA1-1	6399	77	6067	479	3190	953	2902	2307	1465	3422	605	6502	7	7563	6544	1811	4311	2882	2188	3472	1488	4072	545	3729	316	5587
BRCA1-2	3628	6	3148	172	1687	419	1371	828	844	1125	361	2180	46	3336	2820	821	1997	1010	856	1104	692	1567	271	1258	117	1580
BRCA2-2	166	0	185	22	92	23	87	40	30	87	28	157	0	199	487	111	220	108	78	135	48	121	8	98	11	141
BRCA2-3	13631	31	11049	515	5837	1796	5071	3576	3156	4837	1068	9934	19	12620	14130	3437	10141	5576	3736	5379	3553	7459	907	6688	585	7958
BRMS1-2	2833	82	2580	337	1405	515	1339	1170	683	1274	334	2506	10	2243	2451	1174	1449	1647	555	1526	2188	1791	732	1526	450	1889
BRMS1-3	1420	11	1038	150	697	303	453	661	240	796	103	1270	3	1808	878	488	481	533	207	604	665	798	194	641	130	978
CARM1-1	818	4	661	23	405	168	372	244	191	372	76	578	4	889	2070	215	1389	349	463	341	1326	335	382	293	178	255
CBX2-1	935	7	867	138	473	243	279	454	155	606	94	1210	6	1133	946	438	724	681	317	886	6	662	2	873	6	716
CBX2-2	318	0	332	33	190	64	116	143	88	175	16	289	2	377	365	191	225	311	103	345	29	375	12	285	9	247
CBX4-2	579	37	315	177	148	171	110	535	68	564	38	1173	26	1787	416	379	375	588	158	493	1640	262	724	512	351	790
CBX4-3	242	3	244	16	114	44	97	81	54	140	32	253	0	376	275	285	166	362	58	356	842	258	346	280	132	234
CBX5-1	570	2	572	33	240	94	217	137	212	218	55	381	8	558	2375	84	1596	150	991	172	1332	207	407	205	201	270
CBX5-2	1967	19	1776	118	959	363	727	685	405	1072	164	1710	11	2481	8047	331	6838	732	3128	734	3882	977	1509	1056	846	1277
CBX6-3	16254	90	12710	1965	6801	3622	5571	6878	3038	9164	956	14580	79	15775	13122	5539	10212	10357	4729	10067	29008	4966	13069	6181	10074	10119
CBX6-4	1318	26	852	194	517	485	509	896	253	1277	147	2699	5	4622	1399	908	762	1245	416	1442	5948	1495	1631	1409	1195	2035
CBX7-3	7771	45	6242	825	3258	1730	2882	3441	1630	4462	779	9224	17	9468	363	2469	240	3648	185	4230	2086	3740	934	4292	586	5820
CBX8-1	2043	89	1686	185	1061	469	931	1116	345	1138	183	2357	13	3373	130	1055	102	1648	31	1519	513	1917	157	1602	131	2187
CBX8-3	1940	2	1900	77	1095	193	860	324	781	399	314	1112	143	1471	588	222	571	338	179	567	1829	394	1007	455	446	579
CDK4-2	1801	5	1358	146	734	339	606	738	369	1068	175	2133	14	4374	5970	1022	4068	1568	1051	1264	8174	1295	2778	1472	1448	1976
CDK4-3	1996	3	1536	135	830	337	633	687	330	740	128	1428	3	1678	2184	415	1807	677	880	944	4908	862	1930	953	1185	1506
CDX2-1	9551	146	7202	1624	3782	3053	2802	5665	1890	7516	1011	12736	68	11331	793	12348	405	13884	250	11036	208	17760	220	12031	194	13713
CDX2-4	5523	21	4479	543	2192	1263	1697	3014	756	4324	356	6877	10	6415	143	4777	102	5711	37	4281	58	5949	78	4633	70	4619
CHAF1A-1	16736	273	14159	1036	7118	2482	6378	5219	3387	7331	1555	14584	14	16138	11068	3927	7671	5861	3355	7637	2513	9773	922	9146	303	11804
CHAF1A-3	2182	4	1865	140	844	265	789	499	444	685	169	1330	7	1524	1750	318	1161	412	660	589	254	795	154	822	38	789
CHD3-1	758	1	584	48	327	137	315	295	167	337	59	621	1	766	1566	461	858	531	293	409	7965	533	2333	471	1331	604
CHD3-2	277	1	226	47	125	90	114	164	34	159	23	380	0	694	82	89	69	233	33	262	275	222	127	294	95	466
CHD4-4	1279	5	955	99	725	218	481	446	318	652	116	1163	7	1339	7605	441	4896	591	2147	615	5124	822	1497	627	830	765
CIITA-3	8537	299	6293	1027	4510	1302	3523	2832	1761	3598	1180	7566	26	10303	144	1210	107	2276	70	2683	174	3852	114	2962	92	4371
CIITA-4	7008	32	5635	452	3060	1235	2511	2393	1642	3206	582	5297	29	5889	128	1469	71	2377	36	2244	101	2074	77	2424	26	3254
CREBBP-1	20054	105	17627	2261	9320	4233	7112	8216	4290	11706	1884	18666	129	16797	16559	5145	14077	8585	5519	8917	20758	6008	11437	8665	6081	9415
CREBBP-3	848	1	749	71	352	126	322	259	205	353	107	718	4	872	284	132	190	199	113	350	526	197	251	247	174	410
CTCF-5	8907	34	7989	797	3893	1568	3054	2537	1640	3299	640	6683	61	7193	8520	1962	6455	2973	3265	3595	6176	3608	2856	4220	1134	4048
CTCF-6	261	0	230	26	144	66	144	152	64	131	13	184	1	229	458	110	311	145	100	133	314	175	90	141	35	142
DGCR8-1	2954	14	2567	217	1378	545	1098	1190	625	1694	265	2745	7	3453	1810	1701	1211	2098	347	1544	2096	2282	567	1659	365	1898
DGCR8-2	9137	38	7663	835	4258	1731	3101	3084	2031	4563	943	8302	18	8664	2681	2417	1910	4160	777	4112	3219	4359	1369	4675	755	5476
DICER1-1	5261	11	4633	385	2453	756	1964	1467	1217	2140	443	3466	15	5809	5764	1166	4192	1974	1774	2249	4886	1910	1852	2177	955	2951
DICER1-5	21462	83	19114	1617	10662	3577	9016	6650	5390	9962	2404	17992	70	17598	16120	4364	14099	7065	6732	8424	18547	8565	7076	9022	5044	13262
DMAP1-1	11952	36	10976	1396	5351	2415	3707	4173	2268	5560	924	9460	58	14596	9971	2626	8546	4601	4161	5269	8130	5485	3777	6005	1930	7212
DMAP1-2	13285	879	12143	1598	5990	2701	4319	5668	2677	8030	1318	12943	36	13470	8587	4955	6280	7868	3426	9527	6936	8163	3416	10443	1470	10415
DNMT1-4	16405	55	15292	1300	8212	2832	6636	4838	4027	7307	1567	13484	122	13279	13355	2525	12258	4213	7185	6105	8664	6844	3082	7389	1937	8111
DNMT1-6	13240	39	12146	1147	5759	2417	4563	4727	2776	6463	951	11896	31	11249	14326	5258	10736	8027	5854	9495	6891	11157	2832	11279	1109	10940
DNMT3A-2	15392	63	12553	912	6336	2142	4984	5037	3031	6543	1229	12205	50	13716	1630	3979	1031	6137	460	5172	3033	5691	1122	5511	686	7422
DNMT3A-3	5096	7	4830	307	2281	655	1604	1210	736	1889	385	3730	11	3700	414	1159	297	1760	183	2468	908	2450	428	2705	164	2906
DNMT3A-4	105	0	70	6	44	11	29	30	11	64	15	149	0	131	3	18	0	25	1	25	14	57	5	52	4	46
DNMT3B-3	3154	17	2661	305	1659	733	1211	1183	814	1827	332	2678	20	3541	25863	807	21433	1346	9880	1380	85	1426	111	1335	114	1661
DNMT3L-1	6320	5	5219	398	2830	1060	2386	1924	1199	2520	544	4184	2	6001	122	2010	80	2268	54	2130	119	2805	52	2013	14	2764
DNMT3L-3	3453	27	2948	433	1556	1408	1384	2748	529	3423	254	5932	12	6433	126	3102	89	4440	34	3212	59	4472	18	2754	10	4054
DOT1L-3	490	4	653	37	304	76	251	172	139	217	41	348	1	193	708	235	476	446	271	523	233	334	113	405	54	609
DOT1L-5	3866	986	3562	1188	2158	1025	2456	1122	1500	2344	637	5087	397	5610	4324	1366	3727	2426	1852	3200	3579	4238	1399	3530	564	3719
EHMT1-1	3987	9	3738	350	1960	728	1453	1379	852	1971	386	3420	14	2630	3457	479	2526	927	1370	1303	2303	1260	1150	1748	481	1568
EHMT1-2	1375	0	1093	147	669	242	621	500	321	714	158	1412	0	1327	695	211	577	279	305	515	497	406	193	421	126	645
EHMT2-2	115	13	70	31	20	24	32	37	88	327	77	868	14	1120	290	81	213	171	166	296	176	398	96	366	27	382
EHMT2-3	1749	12	1658	108	759	144	538	386	324	434	195	862	9	1009	909	189	626	300	428	431	504	395	236	632	109	529
EIF2C1-1	9034	32	7754	738	4265	1337	3591	2671	1930	3919	944	6565	40	7662	7257	1500	5518	2493	2989	3149	6076	4261	2382	3648	1474	5229
EIF2C1-4	9352	35	7271	700	4485	1781	3742	3116	2160	4092	934	8193	26	10205	9532	2059	8304	3555	3517	3631	6819	5232	2768	4638	1755	5570

EIF2C2-3	569	0	465	44	213	85	224	184	123	201	42	367	0	493	503	168	353	179	144	158	333	238	106	208	33	135
EIF2C3-1	2970	103	2245	263	1520	509	1324	1033	1050	1410	349	2850	95	3646	2683	785	1780	1102	824	1257	2991	1766	887	1254	536	1763
EIF2C3-2	5997	20	5337	429	2867	836	2128	1513	1254	2202	482	3816	25	4167	6085	1270	4136	2037	1741	1840	4709	2074	1607	1918	885	2788
EIF2C4-1	221	4	252	27	123	79	109	163	43	123	12	207	0	265	85	79	64	138	24	127	141	142	40	141	15	144
EIF2C4-4	8796	42	8492	806	4025	1733	3226	3419	1863	4836	726	8403	32	9792	3510	3606	2619	4898	1081	5291	5125	5917	1966	5555	770	6585
ELP3-3	456	1	401	38	214	87	128	169	83	174	28	354	0	512	145	67	97	95	65	204	103	117	84	180	35	203
ELP3-4	48	0	35	3	20	6	20	17	4	29	2	33	0	169	18	17	14	20	6	9	9	16	2	12	1	8
EP300-1	4326	41	3850	291	2229	644	1837	1359	853	1670	445	2663	47	5793	4102	913	3430	1378	1463	1733	4078	1613	1721	1694	1046	2708
EP300-2	2764	13	2412	38	1360	98	1067	171	752	249	262	444	39	558	3051	115	2540	187	1354	177	3253	171	1480	204	916	249
EP400-2	7876	66	6387	578	3368	1372	2959	2788	1434	3612	608	6709	13	8920	5494	2274	4026	3170	1576	3309	5555	3162	1696	3309	1116	4962
ERAS-2	627	5	576	139	261	270	189	576	112	907	46	1644	4	1984	42	793	19	1152	8	974	16	1178	36	1182	5	1254
ERAS-3	910	6	801	119	425	273	321	596	168	817	67	1432	15	1864	76	693	37	908	31	1067	26	1148	56	962	23	952
ERCC6L-1	4895	56	4770	872	2491	2074	2029	3975	1113	5300	531	9409	8	8746	11455	3766	6609	5102	2707	5996	1615	10701	413	7416	306	9760
ERCC6L-3	703	7	498	118	239	305	225	626	142	1028	78	1635	2	2631	1360	841	752	1160	214	756	193	1237	21	873	9	1113
EZH1-2	6606	20	5917	577	2823	1155	2465	2147	1518	2987	644	5659	22	5181	1045	2506	619	3212	305	3610	2308	3265	975	3493	464	3838
EZH1-4	4729	13	4370	370	2305	694	1636	1265	1036	1906	375	3739	38	3960	1011	1793	634	2499	313	3131	2131	2093	1011	2808	358	2473
EZH2-1	3510	16	3123	296	1542	623	1157	1312	572	1951	332	3563	7	3646	4759	844	4036	1736	1756	1759	614	2261	263	2218	136	2659
EZH2-3	3441	288	2926	515	1734	460	1873	647	1397	2902	759	6702	175	6524	7498	1040	6129	1702	3302	2691	1615	3743	520	2927	429	3914
GATAD2A-1	3621	2	3265	261	1826	559	1773	1376	900	2082	417	3531	12	2595	4161	466	3086	869	1477	1052	3419	1164	1109	1260	831	1918
GATAD2A-3	37081	124	32227	1358	18556	3793	16490	7637	12307	11878	5508	23403	74	27564	29387	4404	26264	8451	13422	9776	22803	9244	10423	11693	6108	19640
GSG2-2	3	1	7	3	1	1	3	4	3	2	3	7	0	17	0	1	0	6	0	3	11	4	3	3	2	4
GSG2-3	81	0	81	6	48	22	39	24	22	37	6	61	0	56	83	23	46	17	12	33	6	48	0	20	0	28
GTPBP1-1	11088	78	10285	852	4747	1846	3596	3120	1792	5715	592	9132	26	9335	5212	1684	4226	3314	1696	4564	3538	4165	1918	6084	571	5935
HAT1	289	0	237	16	109	38	111	82	42	145	23	234	1	293	2741	101	1882	153	743	151	963	147	392	168	164	162
HCFC1-3	5929	690	7289	1168	1715	1207	3552	2036	3472	2687	351	5968	500	8420	7007	674	5687	1218	2832	1757	5443	2046	2009	1930	1183	2807
HDAC1-2	755	7	663	89	350	162	277	346	133	349	67	632	5	815	1595	145	1131	217	535	231	1410	217	497	211	296	340
HDAC1-3	8003	14	6591	861	3624	1572	3017	2992	1829	4004	826	7048	134	7059	13040	1405	12674	2688	5835	2930	13794	2333	6210	2713	4178	4902
HDAC10-1	1586	2	1564	53	778	137	622	389	325	524	99	825	2	497	120	127	104	196	50	225	360	238	149	259	66	476
HDAC11-1	2086	2	1669	175	832	369	663	748	348	918	174	1805	0	2115	210	1074	144	1432	56	1254	316	1699	96	1153	29	1575
HDAC11-2	2757	1	1970	194	1092	375	743	767	473	1134	238	2121	2	2312	240	864	108	952	30	770	133	951	60	780	28	876
HDAC3-1	2342	15	2172	302	1060	707	866	1498	476	1861	162	3294	44	5014	11172	1319	8108	2082	2555	1704	7132	2199	2055	1843	1171	2351
HDAC3-3	5669	16	4554	538	2807	1052	2784	2604	1678	3376	650	6075	17	6218	5904	1524	4521	2336	1431	2007	4373	2157	1496	2109	886	2633
HDAC4-1	9427	47	7179	1111	4616	2266	4016	4613	1884	5826	744	9592	47	9681	3336	2853	2430	4664	1032	4227	3623	5580	1676	4801	804	6658
HDAC4-2	237	0	149	5	101	22	58	30	47	53	22	174	2	541	51	23	23	27	5	15	45	13	3	14	3	28
HDAC5-1	33	0	31	0	17	1	12	4	0	6	2	24	0	30	0	4	0	4	0	6	2	3	0	2	0	2
HDAC5-3	852	0	805	45	494	96	362	204	140	188	40	410	0	690	162	146	86	250	39	272	223	296	66	230	22	325
HDAC6-1	208	0	181	51	112	103	77	190	30	178	23	342	1	663	250	177	152	304	45	252	125	304	33	250	11	305
HDAC6-2	149	0	115	32	87	51	77	118	28	138	5	263	4	598	508	182	283	197	99	186	242	287	79	211	29	268
HDAC7-2	2814	7	2776	152	1343	409	863	766	576	1124	260	1832	6	2370	372	673	311	952	149	1235	761	813	390	1181	134	1291
HDAC8-1	9508	32	7843	628	3788	1301	3269	2641	1447	4020	678	6526	51	7829	686	2831	641	3504	229	3712	352	4185	106	3531	189	4976
HDAC8-3	8580	374	7463	1469	3763	2573	3161	5373	1734	6863	835	11892	57	12810	6869	3970	4842	6350	2028	6803	7014	6685	2552	7030	1529	8601
HDAC9-1	7776	38	6187	636	3866	1179	3229	2202	1917	3379	836	6518	32	7274	4677	2782	2984	4250	1309	3935	2303	4668	605	4054	409	5139
HDAC9-3	195	0	124	5	76	6	38	37	18	37	4	70	0	136	26	27	8	39	5	18	0	28	2	33	2	48
HESX1-1	1621	3	1857	146	968	368	658	664	416	956	118	1204	10	1410	950	491	633	746	249	640	58	724	82	772	11	602
HESX1-2	769	1	641	58	400	127	222	264	197	430	66	877	1	946	268	220	230	347	101	455	15	689	15	683	9	742
HIF1A-1	1371	5	1285	107	652	281	561	658	309	852	142	1528	12	1898	8609	190	6594	370	4480	621	20480	377	9973	447	7903	835
HIF1A-2	2948	8	2386	229	1337	486	976	891	540	1220	387	1809	62	2687	11318	436	9546	689	3971	621	17235	406	7439	453	6198	797
HOXA1-2	12393	32	10534	620	5955	2018	4911	3634	2390	5032	1206	8531	38	12784	1105	5732	832	7268	311	6192	1674	8577	678	7194	272	8883
HOXA1-5	2222	6	1647	245	894	542	793	1220	382	1540	212	2817	0	3595	75	1706	116	2145	7	1722	117	2733	55	1582	28	2183
HOXB1-3	6827	28	5517	485	2990	1132	2440	2172	1469	2850	689	5186	15	5702	285	3027	212	3868	110	2974	114	3877	110	3624	47	3464
ING1-4	396	0	309	43	181	93	166	180	62	170	18	318	1	287	558	129	272	149	90	117	117	233	48	114	10	142
INO80-1	4152	11	3354	265	2140	559	1568	1145	843	1555	325	3106	12	3803	1430	1551	979	2254	262	2567	2317	2780	644	2710	353	3281
INO80-2	1393	351	906	2044	564	4399	387	7202	203	7823	126	12382	9	16362	260	6312	221	9849	89	7803	494	9666	191	8751	117	12343
JARID2-3	8817	317	8159	1103	3804	1940	3847	3258	3258	4550	766	10676	406	14398	11438	990	9267	1950	5238	2472	1744	4310	578	3722	332	5084
JARID2-5	6333	22	5833	469	3013	948	2043	1623	1099	2490	366	3912	37	4463	15036	594	14900	1521	8553	2491	2426	2789	1331	4294	417	3224
JHDM1D-1	5262	9	4859	465	2602	1315	2056	2375	896	2951	359	5366	9	7391	2823	2575	2107	4162	781	3494	1635	4684	422	3559	233	4993
JHDM1D-2	1353	2	1231	100	656	187	542	370	272	438	108	848	2	1217	1063	698	659	796	250	704	538	857	193	860	80	839
JMJD4-1	2942	23	2615	315	1389	759	1096	1514	550	1813	195	3043	39	4468	806	1704	469	2174	143	2188	596	2635	206	1916	89	2291
JMJD4-2	4190	17	4457	295	2042	736	1796	1441	970	2497	400	3707	3	4710	876	1512	501	2038	162	1862	816	2208	484	1954	139	2263
JMJD6-2	4199	10	3691	444	2117	725	1432	1485	901	2238	329	3625	11	4177	2223	1249	1415	1947	629	1593	3671	2044	1353	1858	559	1941
JMJD7-2	1582	6	1318	89	675	230	572	443	345	574	132	1100	7	1266	513	480	386	762	137	660	320	613	118	552	63	846
JMJD7-3	245	47	150	81	31	68	49	111	94	232	50	442	0	622	121	310	71	389	39	458	178	693	58	502	19	526
JMJD8-1	336	0	220	29	135	70	108	152	49	308	55	501	1	542	1036	121	866	244	364	197	1855	183	794	221	475	291
JMJD8-2	1408	448	1602	459	1223	443	1546	524	270	841	97	2171	130	2477	2550	434	1808	761	887	843	7701	1278	2681	1101	1317	1459
KANSL1-5	3062	12	2982	57	1666	102	1381	186	599	247	234	373	39	448	4422	216	2901	296	840	255	6661	343	1797	264	792	369
KAT2A-1	4162	13	3213	374	1758	613	1464	1246	880	1917	451	3501	12	4063	2789	1497	2182	2189	700	1946	2685	2313	974	1831	429	2477
KAT2A-3	2697	5	1955	194	1131	359	878	658	462	889	189	1698	24	2942	947	775	541	830	217	855	1007	1007	399	941	169	911
KAT2B-2	5643	8	4549	264	2226	734	1904	1191	836	1571	511	2839	14	4122	334	1644	183	1903	71	1544	1587	1907	603	1590	263	1587
KAT5-1	993	5	739	99	408	155	326	326	175	418	75	858	1	1229	522	503	319	602	126	586	812	686	266	625	125	800
KAT6A-1	3826	15	3229	296	1909	497	1376	1235	653	1389	247	2406	10	3705	1456	606	1271	1329	542	1353	2309	1241	1043	1459	567	1910
KAT6A-2	5837	14	4850	383	2485	809	2082	1698	1126	2451	469	4664	7	4976	1697	708	1353	1515	673	1940	2568	1419	952	1801	689	2609
KAT6B-2	860	2	629	42	363	127	345	254	134	236	57	458	5	797	424	211	303	378	144	406	1062	428	361	315	228	573
KAT6B-3	4358	36	3409	272	1899	509	1354	917	845	1255	410	2167	34	4475	1478	977	814	1246	319	1027	3277	1313	1002	1024	680	1487
KAT7-1	5330	56	4558	268	2426	875	1803	1719	875	2398	370	4343	4	6336	4646	878	3899	1681	1881	2200	4005	2004	1510	2412	951	3581
KAT7-2	2834	39	2069	180	1092	423	934	948	515	1111	223	2247	60	7584	3167	775	2094	1075	772	936	2528	1119	755	1182	435	1666
KAT8-1	793	3	682	87	436	229	322	420	116	477	69	854	1	1642	1032	432	690	641	326	676	1036	931	386	727	203	1005
KCTD3-1	3394	3	2754	219	1390	488	1188	1065	526	1064	247	2106	9	3027	7289	425	6055	753	3424	1080	5414	940	2398	1165	1536	1817
KCTD3-2	4101	11	3526	377	1642	554	1603	1353	874	1597	431	2599	39	2718	6710	795	5058	1192	2222	1477	5220	1410	1902	1457	1244	2139
KDM1A-1	9688	18	8544	732	4741	1414	4178	2601	2433	4145	1072	7529	78	7903	18476	1094	17079	2005	9786	2550	9859	3675	3854	3176	2124	4938
KDM1A-3	259	0	288	23	145	55	111	146	54	168	35	267	1	347	4580	178	3045	250	1165	229	928	254	206	184	106	265

KDM1B-2	3879	81	3651	278	1826	635	1705	1425	837	1642	334	2906	23	4870	1911	1315	1328	2022	500	1865	2623	2083	769	1783	640	3027
KDM1B-3	4974	10	4129	263	2243	508	1768	1079	859	1464	357	2637	21	4738	912	696	624	1386	285	1501	1989	1666	646	1565	272	2034
KDM2A-1	179	0	151	11	91	26	86	39	39	79	12	105	0	196	121	42	55	44	24	48	335	124	88	74	39	87
KDM2A-2	11228	30	8715	952	5104	1770	4385	4168	2150	5250	1112	9717	20	11571	3645	3147	2484	5073	955	5452	6461	4576	2166	4504	1286	7085
KDM2B-1	7059	23	5679	408	3199	721	2859	1636	1644	1895	822	3101	79	4270	17029	879	14277	1651	6198	1585	4786	2279	1459	1881	1059	2901
KDM3A-1	4057	1	3445	303	1887	548	1487	964	870	1527	470	2442	40	3178	2611	790	1898	1086	744	1440	2572	1361	910	1371	420	1681
KDM3A-3	4716	16	3612	287	2081	673	1692	1528	916	1845	316	3402	9	4483	3899	1417	2861	1840	1051	1948	3609	2193	1212	1608	589	2308
KDM3B-2	14225	119	10432	1277	5952	2538	4710	5292	1635	5510	735	9391	41	15781	11031	1625	11570	3330	5104	3995	9943	4479	4449	4411	2787	7272
KDM3B-3	4967	6	4466	425	2340	872	1686	1712	1005	2389	478	4658	6	6307	4325	1674	2680	2463	848	2214	2363	2208	720	1986	367	2256
KDM4A-1	1775	6	1304	232	830	539	611	1020	280	1168	131	2179	6	3136	2491	692	1781	1197	843	1087	1687	1492	617	1132	320	1804
KDM4A-3	185	1	145	19	79	32	46	48	38	84	17	171	0	199	267	54	161	73	102	55	134	91	60	54	25	73
KDM4B-1	861	2	768	81	403	195	326	415	153	468	59	822	0	1080	151	307	79	548	42	532	184	518	38	406	41	523
KDM4B-2	2138	5	1845	149	1048	396	816	776	536	965	230	1831	3	1923	2424	1524	1593	2127	634	2246	2396	2293	847	2018	456	2426
KDM4C-4	2875	1	2469	170	1486	418	1133	839	704	1315	397	2613	14	2921	435	327	256	518	126	498	936	833	263	870	212	773
KDM4D-1	6522	22	5641	415	2722	1019	1832	2092	988	3235	293	6184	8	6423	445	3070	285	4253	47	4756	135	5277	37	5393	16	5435
KDM4D-4	1015	43	832	174	372	275	268	552	113	676	42	1158	3	1102	35	636	15	756	18	661	19	792	14	824	3	815
KDM5A-1	1444	2	1138	108	561	238	425	537	225	741	92	1350	3	2133	1384	564	821	709	246	498	1718	691	363	453	197	627
KDM5A-3	3581	8	2871	241	1704	512	1303	1093	606	1190	243	1982	11	3425	1521	503	1309	843	676	1110	2779	1038	986	1112	788	1801
KDM5B-1	13788	47	11568	1316	5868	2523	5547	4775	2966	7816	1322	13083	49	14718	27753	1727	24542	3381	13031	4360	22650	5255	9364	5512	6054	8814
KDM5B-3	10541	55	7709	744	4857	1834	3891	4234	1822	4735	650	8204	30	12146	16124	1155	14225	2457	6566	3011	12156	3385	4352	3436	3127	6193
KDM6A-1	83	0	109	14	32	33	35	69	23	113	9	282	2	508	585	128	417	225	191	263	463	370	189	353	69	450
KDM6A-2	5040	186	5343	985	2330	2023	2250	4057	1008	5060	661	11560	11	9555	9123	1878	6968	3262	3642	4576	6400	4552	2592	5238	997	5044
KDM6B-4	3543	18	2806	329	1436	679	994	1123	550	1599	263	2968	5	3252	2372	1010	1433	1350	624	1719	3939	1415	1516	1392	717	1480
KDM8-1	4307	63	3597	544	1637	1130	1544	2352	575	2626	226	5310	32	7434	320	3069	285	4729	94	3801	216	4707	94	4273	36	4904
KDM8-4	1544	1	1461	212	813	602	679	1074	370	1597	103	2509	2	2203	107	559	34	915	15	968	101	1089	76	1101	14	1085
LBR-1	1666	4	1391	89	796	268	751	537	311	642	127	1072	5	898	4461	111	3860	262	2004	396	1407	496	515	479	301	772
LBR-3	3120	7	2664	411	1588	1180	1282	2222	645	2758	310	4716	4	5913	4489	595	4109	1154	2752	1701	1942	1701	784	1925	617	3271
LEFTY2-3	450	2	371	60	219	156	185	272	74	370	53	629	0	854	497	495	274	554	111	530	15	694	6	496	5	742
LEFTY2-4	3024	5	2554	169	1448	528	1076	1068	558	1427	220	3012	0	3337	2027	1053	1100	1355	374	1613	33	2469	9	1858	1	2318
LMNA-7	22505	110	18960	1926	10124	3774	7824	6553	4626	9935	2429	16799	89	16607	19435	4297	20012	8490	9721	9303	43904	2013	31101	3631	22187	5326
LMNA-8	3434	628	3599	810	2719	826	2698	873	742	1331	334	2476	164	2659	5643	1067	4588	1779	1714	2092	25507	976	13007	1437	7630	1705
LMNB1-2	3019	70	2393	103	1700	48	1659	129	851	980	447	2224	151	2351	12137	488	10444	742	5063	1094	1069	1228	273	1218	195	1499
LMNB1-3	13412	77	11638	783	6788	1600	5142	3448	2768	4620	1206	7942	129	8415	16624	934	16731	1758	8143	2408	3653	4491	1901	4981	1160	6116
LMNB2-1	11358	35	10920	1185	5054	2040	4149	4508	2625	6019	857	10994	29	10729	17923	2264	16325	3467	8436	5029	12230	6060	5311	6761	2606	9124
LMNB2-2	878	9	837	104	344	253	334	339	149	449	72	1081	3	916	1385	261	1141	377	539	498	727	502	316	552	168	506
LMX1B-4	2778	4	2562	211	1372	448	1179	1017	541	1083	238	2397	14	3068	61	800	62	1277	23	1427	75	1443	96	1346	25	1813
MAD1L1-2	4642	18	3900	236	2125	695	2076	1601	1026	2064	450	3689	10	4463	1710	1328	1295	2079	462	2085	1455	2551	548	2320	262	3117
MAD1L1-3	1469	21	1248	153	585	416	411	591	200	618	86	1210	4	1458	687	464	490	694	229	799	554	725	294	857	118	1021
MAK-1	4630	28	4241	404	2067	683	1442	1129	883	1860	398	3425	17	4999	1049	1050	761	1614	330	1882	928	2017	385	2134	137	2316
MAK-2	4064	10	3865	258	1950	664	1780	1427	1151	1959	456	3296	13	4767	953	1007	884	1637	277	1732	758	1753	295	1612	135	2351
MBD1-3	242	3	266	22	108	72	98	140	58	183	9	221	2	308	499	145	313	197	82	158	800	194	243	179	72	151
MBD1-4	16186	46	13296	1341	7077	2945	6183	5871	3953	7757	1609	14987	83	14943	6009	7217	4699	10927	1576	9474	10039	10850	3951	9111	2214	13368
MBD2-1	1564	11	1405	105	658	240	546	503	389	689	158	1379	1	1119	1	455	2	688	0	821	475	725	184	730	118	1097
MBD2-3	6762	4797	3798	6617	757	6386	1138	6654	3679	6966	1605	14372	25	18384	534	4577	340	8399	156	9346	8088	10492	2507	9399	1158	12737
MBD3-1	567	1	501	27	276	41	228	118	122	152	99	235	17	257	221	81	130	97	41	103	207	148	74	123	58	176
MBD3-3	619	0	576	34	381	50	209	102	132	110	86	292	10	379	5344	92	4403	153	1653	160	8368	175	3304	198	1374	163
MBD4-2	124	0	85	9	38	16	46	56	31	71	15	154	2	193	548	96	240	88	93	52	305	100	62	59	28	69
MBD4-3	999	0	845	59	459	175	378	381	224	458	67	867	2	1297	1161	161	849	266	451	344	998	329	368	375	255	653
MECP2-1	2544	21	2796	459	1409	953	1040	2017	631	2302	570	4023	97	4306	1861	1476	1221	2483	574	2676	2708	2862	1016	2419	552	3221
MECP2-3	9231	63	8559	1631	3961	3362	3356	6135	1786	7892	840	13276	67	12402	6566	3770	5137	6375	2443	7496	9301	6154	3722	6117	2270	7961
MEIS1-2	1022	5	860	151	383	270	298	579	179	772	77	1486	5	1603	163	1155	79	1447	42	1756	3772	696	1584	996	801	1215
MEIS1-4	14453	84	12041	1233	6722	2472	5538	4796	2761	6097	1440	11254	42	13269	2041	8019	1280	9754	409	8577	27890	4616	11803	5101	7947	8132
MEOX2-2	1090	1	687	41	426	108	307	209	159	284	83	542	0	673	110	271	61	294	16	284	13	425	10	229	7	282
MEOX2-4	14684	21	12561	490	6935	1352	5236	2832	2839	3974	1040	7400	22	12569	908	4796	764	6963	202	5676	493	6319	242	4533	56	7151
MLL-5	2279	4	1771	123	974	334	806	699	401	749	210	1362	9	2186	2974	506	2103	745	794	876	2797	772	1165	885	651	1011
MTA1-1	137	0	169	14	86	23	82	57	31	67	12	186	6	215	3384	83	2970	112	1165	134	1944	114	620	120	467	152
MTA1-2	1400	60	1149	102	819	104	933	150	348	680	151	1596	128	1956	2948	191	2376	390	1132	554	3592	766	1085	726	672	1028
MTA2-1	3640	4	3116	224	1868	502	1442	1180	822	1577	417	2656	15	2124	5441	560	4401	885	2156	1009	3345	1081	1276	1415	722	1732
MTA2-2	3020	7	2748	214	1473	561	1064	1094	764	1371	271	2326	11	2947	3750	529	2727	894	1381	999	2733	1026	1091	1110	399	1165
MTA3-2	6163	9	5186	263	2937	692	2240	1595	1225	2109	564	3939	21	5157	12557	669	11572	1131	5733	1589	6787	1533	3152	2039	1888	2817
MTF2-2	12837	48	11426	745	5709	1671	4752	3048	2411	4535	954	9183	30	11935	18498	735	17045	1228	10728	2251	5114	5348	2271	6223	1339	7521
MTF2-3	3012	10	2621	394	1403	692	1146	1532	603	1817	331	3030	68	5029	11697	696	9636	1271	4086	1520	993	1971	325	1784	249	2680
MYL4-2	8899	12	8097	636	4323	1440	3270	2894	1954	4107	761	7664	3	8415	710	3706	422	4820	220	4872	190	5156	111	3936	28	5249
NANOG-5	398	0	378	11	226	12	162	65	61	69	19	123	0	153	64	45	39	54	12	60	15	74	9	61	0	84
NCOR1-1	4477	16	3323	399	2071	929	1518	1824	642	2005	248	3240	7	4915	3527	650	2975	1384	1320	1636	4752	1067	1862	1443	1443	2472
NCOR1-2	1815	7	1635	183	854	347	596	660	448	1076	170	1976	13	3262	3232	706	3200	1120	1107	1114	4028	898	1908	1271	1108	1701
NCOR2-3	572	2	432	38	258	98	196	149	115	234	48	441	0	561	1187	246	837	390	335	334	1159	378	431	331	202	416
NCOR2-6	492	22	417	45	287	53	215	47	189	316	103	639	11	669	1341	148	1065	291	497	311	2847	490	877	367	505	460
NEUROG1-2	2626	33	3084	673	1374	1193	1470	2195	745	2865	239	4835	18	4031	164	2389	169	4478	81	4911	79	4800	154	5390	43	6373
NEUROG1-3	2845	6054	1799	6712	600	6030	677	6456	1498	4909	526	8153	73	6477	429	4471	177	6583	180	6543	160	10500	135	7067	45	9152
NKX2-2-1	1077	4	1072	82	444	147	387	341	196	658	116	1160	2	1304	149	1005	51	1376	17	1511	92	1477	59	1419	13	1637
NKX2-2-2	802	2	752	86	364	187	313	297	207	428	80	1129	0	1788	80	612	27	493	6	452	16	781	19	592	1	682
NODAL-1	3877	15	3907	387	2135	935	1747	1869	1002	2899	478	4965	11	4826	204	1821	164	2545	53	2326	116	3306	35	1984	16	2873
NODAL-3	17764	46	15561	1363	8028	3025	6677	5942	3531	9122	1637	15058	8	16338	3950	11746	2689	15098	1048	13218	1433	17213	691	14322	166	15036
NSD1-2	11025	26	9735	882	4744	1812	3719	3599	2260	5160	1068	8788	45	9636	10254	2857	7104	4822	3358	5258	6927	4962	2440	4843	1610	6596
OGT-1	5073	52	4567	551	2227	1248	1713	2621	926	3214	473	5600	60	5304	7853	814	7090	1669	3329	1887	12827	1274	7403	1732	5145	2483
OGT-2	1586	4	1404	166	851	456	664	983	354	1369	140	2530	7	2783	3245	309	2271	621	1404	965	7142	584	3686	765	2656	1396
OLIG2-1	289	12	258	46	113	83	127	195	68	170	29	357	5	480	50	141	58	175	32	205	2	196	5	247	2	244
OLIG2-2	520	5	303	70	185	144	163	267	58	382	40	777	2	1102	169	547	104	655	59	661	15	712	16	670	0	745
ONECUT1-2	15179	26	13064	770	6902	2039	5894	4096	3104	5075	1436	9808	61	13684	11778	3258	9549	5790	4219	5648	154	8796	133	6625	73	8963

ONECUT1-3	11328	22	8823	679	5165	1680	4851	3334	2447	5578	1212	10103	10	12998	16381	4331	11656	7156	4047	6412	187	8694	131	6733	42	9442
OTX1-2	1909	16	1537	265	705	413	573	936	333	1392	174	2701	1	3934	338	916	229	1284	59	1895	50	1839	158	2169	25	2449
OTX2-5	7201	26	5942	787	3332	1994	2630	4277	1606	5649	701	9914	27	11797	10127	2588	7212	3960	3246	4358	117	7708	82	5063	61	7323
PADI4-3	3706	18	3243	322	1658	529	1529	1249	836	1700	426	2820	29	3351	151	1353	168	2099	47	1882	114	2101	74	1692	33	2220
PARP1-1	11125	255	11980	445	7941	305	8295	613	3114	3603	1404	7796	1345	10126	18028	453	19740	886	12670	1504	11726	3951	4405	4144	2701	5373
PARP1-3	1360	0	1335	69	670	164	599	356	307	580	120	948	2	459	5217	145	3368	243	2027	339	712	400	281	433	146	601
PARP11-1	2425	10	1934	167	1142	284	1015	672	553	996	213	1680	3	2911	702	413	412	626	229	795	898	1008	362	836	244	1466
PARP11-3	1886	13	1661	182	925	333	602	631	362	1017	122	1778	13	3690	539	745	300	990	136	878	753	1210	185	803	118	1034
PARP12-3	413	71	197	64	36	48	61	103	232	548	103	1102	1	1718	59	262	41	404	17	565	304	932	81	692	47	903
PARP12-5	6164	35	5384	608	2985	925	2817	1984	1511	3083	693	5589	28	4063	436	1206	300	1889	121	2122	1205	2549	558	2601	300	3330
PARP14-4	12826	57	10524	1256	5834	2524	4493	4008	3149	5966	1244	11031	47	10953	3830	3489	3036	5604	1208	5424	10864	4225	4936	4714	2590	5865
PARP15-1	9397	12	8010	861	4240	1738	4004	3581	1973	5074	1053	9314	5	9956	226	3174	145	4334	117	4724	289	4638	143	4382	77	5912
PARP16-1	3604	7	3573	292	1806	520	1650	839	929	1306	741	2381	100	2302	739	1168	460	1703	168	1671	1027	1718	388	1508	162	1800
PARP16-3	2284	2	2004	194	1245	577	910	1106	438	1333	181	2308	0	3254	214	732	179	995	51	1144	367	1160	174	1045	72	1519
PARP2-1	4003	23	2612	293	1933	578	1665	1256	724	1377	286	2427	6	3040	2674	796	1781	1037	803	1049	1345	1290	481	1117	253	1484
PARP2-3	662	1	541	27	266	88	260	165	122	193	53	396	3	688	1363	150	999	226	376	193	352	227	120	163	104	280
PARP3-3	4445	34	4522	355	1945	880	1530	1451	962	1974	348	3701	30	4048	263	1413	197	2324	141	2723	2556	1924	1424	2425	551	2339
PARP4-1	8534	55	6594	2568	2935	4872	2107	7679	1227	10600	651	16499	73	17972	5927	5860	3913	8382	1519	8439	6060	9463	1900	8201	1121	11369
PARP6-1	510	7	486	79	300	147	198	251	145	351	70	686	10	1025	408	224	295	271	124	252	1178	335	432	252	201	399
PARP6-4	14852	63	12609	1595	7190	3155	6168	6072	3621	8452	1430	15090	37	18214	4394	6534	3575	8194	1064	7378	11303	6903	3367	5915	2247	9272
PARP8-1	403	1	379	30	185	93	99	259	63	297	13	556	1	955	298	196	137	298	61	412	89	399	20	525	7	483
PARP8-2	15452	49	12339	561	6979	1813	6264	3655	3165	5809	1906	11878	17	12915	12916	3150	9102	5087	3681	5470	5131	7276	1885	6531	951	9142
PARP9-1	488	1	421	22	245	54	175	107	98	145	32	217	1	307	20	70	17	113	5	136	616	173	264	138	93	127
PARP9-4	80	0	57	9	49	36	45	45	20	78	11	128	2	154	2	57	1	64	1	50	163	79	43	64	23	53
PAX7-4	4704	11	4361	338	2382	989	2123	1712	1241	2685	635	4525	4	4575	300	2057	201	2569	41	2641	88	3395	24	2347	8	3156
PCGF1-1	13926	28	12470	1108	7366	1829	5879	3676	4127	5362	3532	8934	429	11458	5878	3025	4091	5144	1672	4803	10928	3745	4006	3954	2467	5716
PCGF2-1	3259	6	2467	327	1261	636	1172	1415	532	1449	240	2784	6	3413	3554	940	2779	1589	1252	1735	5439	1536	2023	1788	1378	2401
PCGF3-1	14517	317	13681	582	6687	1569	5679	2893	3081	4737	1189	9330	54	9155	11361	2895	8086	4590	4457	6642	12164	5542	5859	6331	2714	7229
PCGF3-2	6168	28	4717	461	2514	1064	2099	1885	1163	2666	456	4958	60	5838	3572	1381	2788	2236	1300	2513	5116	2260	2112	2488	1241	3052
PCGF5-1	407	0	372	26	234	60	169	109	46	144	37	317	2	418	13	12	22	52	9	88	689	95	253	120	101	154
PCGF5-2	932	6	614	55	574	183	346	328	239	492	141	1388	14	4439	227	125	162	122	66	189	4044	368	972	329	639	284
PCYOX1-2	1814	3	1297	74	775	224	646	454	251	489	95	697	6	1109	3093	492	1547	598	436	480	9033	697	2146	493	1295	609
PCYOX1-3	12512	38	10976	844	5119	1903	3899	3742	2111	5625	882	11186	204	12243	15543	1065	17551	2631	11013	3733	25664	916	20165	1742	13640	2756
PHC1-2	13764	18	12035	417	6752	862	5209	1746	3815	3015	1729	6641	67	7656	21344	300	24249	660	17832	1010	15559	1404	8281	2417	4006	2613
PHC1-4	10156	529	11177	1213	4911	1578	4167	2042	3866	2449	1043	7149	814	6842	5975	508	6787	1154	6289	2563	2641	4516	1445	6658	471	5098
PHC2-1	5315	35	4089	290	2401	932	1979	1898	1168	2030	512	3811	13	4607	3171	1894	2207	2914	950	2780	5473	2714	2106	2541	1292	3492
PHC2-2	4593	5	3678	381	2082	846	1763	1480	1065	2032	508	3864	11	3709	6125	898	5142	1647	2404	1996	10294	1510	4841	2036	2591	2620
PHC3-5	16026	77	14247	1123	6563	2289	4918	3787	2695	5112	1227	9124	139	9667	8321	4340	5487	6300	2662	6757	21447	3511	11116	5679	4363	4798
PHC3-6	2366	7	2312	169	1492	380	1010	725	760	1179	225	1574	12	1830	2835	936	1468	911	542	891	10390	1169	3219	915	1528	1115
PHF19-2	967	0	779	119	561	220	450	425	203	505	117	959	11	1063	766	269	547	361	253	442	611	385	315	437	147	474
PHF19-3	401	1	361	52	164	76	126	127	65	231	33	389	1	387	377	125	261	180	153	244	426	305	192	392	58	461
PHF8-2	9851	40	7825	1646	4807	3503	3409	6795	1922	8636	851	14464	30	15728	6115	4283	4867	6672	1723	7891	4637	8592	1806	6808	1065	9485
PHF8-3	496	3	443	44	201	146	252	345	127	474	52	1002	6	1478	590	320	230	326	102	284	347	289	66	234	56	343
POLR2A-6	4739	15	4079	268	2161	759	1926	1539	904	2069	467	3471	28	3219	9730	755	8707	1236	4381	1584	8929	1194	4639	1615	2537	1940
POLR2B-1	14617	68	11400	1307	6071	2698	5057	5081	2653	6479	1087	10679	60	14860	24936	1180	24201	2324	12792	3672	20772	3760	9676	4650	6681	7482
POLR2B-2	10127	483	8093	871	4208	2589	3660	5290	1494	5340	589	9409	32	15353	13962	1058	13458	2065	6446	3103	12912	3147	4984	3832	3944	8203
PRC1-2	12820	77	11190	1317	5754	2454	4368	4089	2839	6566	1127	11691	20	12159	75	1854	127	3104	106	4103	7545	5862	2754	6416	1362	6853
PRC1-3	2326	4	1881	173	1016	412	856	809	392	1210	244	2309	3	2837	3379	569	2460	1068	1224	1069	816	1143	312	1212	184	1679
PRDM2-4	1469	4	1089	89	581	232	438	413	302	581	115	1089	6	1321	75	113	48	274	41	448	98	249	57	361	36	513
PRDM9-1	3906	9	3446	133	2049	339	1603	603	825	863	338	1327	131	2317	99	413	66	684	52	583	131	761	127	722	69	764
PRMT1-3	2193	5	2023	81	1155	261	1279	454	998	544	228	1295	96	1644	11110	121	9951	245	5656	301	7521	468	2618	436	1828	659
PRMT2-1	382	2	301	31	124	99	110	150	67	270	28	535	0	661	420	183	279	280	100	306	848	286	223	270	144	288
PRMT5-1	1819	2	1526	138	856	285	708	697	322	771	155	1654	1	2008	1427	277	1152	427	539	552	1095	619	398	593	197	704
PRMT5-2	1890	6	1443	31	1062	104	885	196	457	214	235	329	48	721	1829	88	1283	106	630	144	1424	211	384	168	253	217
PRMT6-1	2086	4	2014	138	924	400	854	735	456	943	185	1708	2	1600	1677	616	1336	779	717	957	1247	1109	394	1052	227	1258
PRMT7-2	6215	18	5325	476	2815	875	2373	1727	1322	2068	524	3815	25	4993	3158	1612	2404	2948	1120	3158	2275	3365	949	3686	417	3699
PRMT7-3	11887	109	11306	828	5128	2030	3947	3191	2207	4668	1087	8517	13	8398	4765	2432	3549	3885	1990	5670	3099	5453	1827	6167	712	6286
PTEN-1	1908	19	1388	131	1189	407	891	854	530	991	241	2301	19	3392	1442	358	966	480	474	657	10170	1188	2935	1030	1627	1176
PTEN-2	1399	7	1271	114	641	291	528	516	421	718	184	1332	4	1814	2208	468	1151	618	529	658	8249	968	2476	817	1447	1151
RAD54L-1	4740	10	3952	296	2127	906	1795	1555	904	2016	482	4151	13	5323	3879	1674	1974	1979	789	1484	121	1808	44	1414	32	2102
RAD54L-2	4410	14	3742	271	2095	451	1537	873	887	1281	369	2717	12	3938	3439	835	2651	1797	1295	2242	1116	2535	414	2857	204	3041
RARB-2	15574	42	12182	1115	7339	2687	6357	5530	3646	6737	1467	13027	88	14859	856	7257	541	9885	195	7489	269	10268	178	6275	29	9831
RARB-3	240	0	163	6	115	32	112	41	80	79	14	169	0	224	70	86	27	83	12	70	22	71	7	63	5	70
RB1-1	5573	18	4663	503	2509	1051	2052	1787	1093	2664	429	4966	9	4849	2601	980	1876	1742	1271	2675	5288	1074	3466	2032	1812	2702
RB1-2	770	3	724	102	334	153	216	361	125	492	67	969	3	1208	444	290	360	666	140	674	1792	604	573	535	311	686
RBBP4-1	1380	0	1255	75	650	155	591	343	258	520	99	770	6	1166	4488	347	2892	427	1049	467	6944	596	2238	497	1171	678
RBBP4-3	2460	14	2478	233	1073	526	702	880	330	1315	158	2263	7	2695	805	907	588	1646	262	2113	536	1930	313	2634	101	2410
RBBP7-1	15614	839	13886	951	10140	746	12134	1091	3159	4128	1208	8873	2629	10904	15916	621	18165	1305	10542	2251	18176	3279	7196	3801	5891	7101
RBBP7-2	546	2	477	66	250	168	206	305	128	371	48	657	5	846	2559	111	1910	143	1214	208	1478	328	815	340	318	440
RBL2-4	15801	49	12949	1282	7139	2508	5928	5071	4014	7799	1785	14732	62	15475	7639	4805	5307	6932	2277	7868	14345	7232	5635	7239	3115	8706
REST-2	3154	7	2802	163	1420	426	1144	805	571	1214	307	2166	18	2973	6103	663	5066	1201	2328	1344	4803	1111	1931	1323	1285	2056
RING1-1	9936	53	8244	837	4684	1783	3529	3644	2131	5100	1193	8602	71	10705	3995	2728	3146	4709	1920	5906	10305	3611	5766	4802	3086	6531
RING1-2	1923	12	1709	209	930	550	643	1027	466	1529	237	2582	6	3028	1683	984	1463	1722	617	2016	5546	1533	2271	1867	1262	2617
RNF168-1	3724	7	3088	225	1826	473	1513	1055	597	1199	247	2348	2	3857	3635	582	2871	993	1095	1054	2601	1336	829	1234	543	1891
RNF168-4	1707	1	1104	18	536	91	532	138	191	207	97	431	1	1330	1589	152	891	154	203	131	686	226	171	166	80	170
RNF2-5	420	0	335	17	157	68	110	75	60	159	24	307	3	448	2129	106	1068	131	413	137	384	129	92	108	65	197
RNF24-1	2300	14	1749	229	956	506	850	1118	501	1334	267	2719	13	3820	2684	1708	1376	2191	588	2301	6321	2526	1987	2218	1117	2950
RNF24-2	911	0	636	53	331	121	245	204	137	270	54	449	0	919	866	272	411	301	113	206	1730	343	397	229	194	266
RNF25-3	1539	4	1184	46	623	114	552	319	257	422	90	986	11	1783	998	320	548	531	158	527	1268	560	399	411	192	543
RNF26-1	6880	9	5894	625	3009	1374	2373	3043	1466	4130	846	7480	3	7617	5615	2456	4171	3622	1817	4189	4381	3852	1520	3853	839	4780

RNF26-2	29	0	22	0	11	1	9	13	3	1	3	14	0	38	131	41	62	23	25	43	51	41	11	51	15	12
RNF31-2	9643	13	9335	520	4759	964	3836	1982	2381	2961	1129	6180	38	6149	4594	2142	2983	3392	1459	3681	4371	3566	1841	3501	705	3872
RNF32-1	11623	65	11390	1365	6390	2304	5006	4504	2802	5787	1266	10455	22	9356	1654	4557	855	5350	389	6591	1079	7770	296	6234	151	7128
RNF32-2	454	0	321	46	152	106	126	226	107	299	19	540	1	900	64	286	28	274	6	223	64	278	11	189	5	263
RNF34-4	9358	34	6954	735	4050	1616	2969	3204	1573	4673	584	7789	95	10617	7705	2385	5464	3601	2007	3687	6768	4367	2253	4419	1235	5463
RNF38-3	3203	8	2545	125	1583	355	1243	660	655	954	274	1964	11	2426	3052	563	1966	854	932	949	2210	1088	642	814	397	1226
RNF40-1	4819	15	4436	428	2254	917	2016	1768	1162	2717	479	4939	17	5166	5081	4067	2983	4924	1337	4786	4616	5248	1711	4606	669	5648
RNF40-6	17056	106	14602	1774	8523	3315	7301	7230	4011	8944	1934	16432	20	15690	6902	9559	4667	11182	1866	9931	9661	13059	2724	9893	1876	12394
RNF8-1	35219	334	30255	2675	16845	6707	13993	13519	9356	16901	4367	31211	85	33968	4058	18678	2154	24139	664	20569	3480	27544	908	20154	365	27012
RNF8-4	842	6	541	75	377	150	228	341	98	310	40	516	1	994	998	282	621	352	205	357	627	444	173	305	56	366
RPIA-1	4196	7	3796	355	1867	820	1220	1527	736	2376	324	3884	5	4537	3619	515	3166	1204	1599	1667	1577	1184	844	1832	473	2371
RPIA-2	20566	21	17604	404	9782	1148	7393	2955	3900	5307	1356	12167	102	17218	19641	933	23080	2720	9669	3105	14448	3105	5995	4430	3260	8863
RPRD1A-1	6732	23	5418	397	3018	1114	2309	2381	1083	4246	344	8990	34	12156	19149	1005	15846	2102	6393	2357	8400	3127	2841	3356	1553	5405
RPRD1A-2	7506	4	5828	433	3665	1076	3017	2183	1396	2779	517	5432	20	7660	11964	818	9814	1477	5487	1807	7793	2185	2923	2158	2070	3595
RYBP-2	30324	81	25096	1752	13970	4240	12113	8347	6130	11462	2946	19591	54	29317	20545	3308	18207	6433	8015	8299	22704	4884	10126	6973	9135	13194
RYBP-3	11708	37	8894	788	5090	1908	3485	4278	1982	5846	826	10763	34	14460	19379	1826	19085	4226	7057	4349	18982	3479	7621	4125	5779	7835
SAP130-2	19416	85	15902	1653	9119	3343	7140	7536	3563	9672	1615	16482	110	21543	16313	2900	15255	5779	7332	7216	10182	9163	4379	8720	2067	12331
SAP130-3	3864	311	3094	717	1797	764	1739	1383	965	1262	295	2694	155	3655	1249	649	1114	1275	543	1446	1117	1930	347	1756	213	2461
SAP18-2	4618	18	5179	249	2450	719	1502	1206	756	1722	307	3081	30	3676	3947	749	3246	1502	1854	2228	4412	1334	2740	2674	1040	2391
SAP18-3	1494	1	1371	47	737	99	643	243	355	314	170	765	2	882	1423	112	1311	140	519	175	2155	233	725	269	341	260
SAP25-1	108	0	106	18	67	28	41	74	34	77	13	183	0	231	743	157	422	186	180	230	170	208	62	200	23	218
SCMH1-3	5617	5	5109	491	2622	1075	2485	1992	1556	2983	676	5818	12	5516	3399	2313	2678	3766	1295	3805	5641	4503	2123	4151	945	4598
SCMH1-4	8794	22	7396	928	4023	1650	3192	2790	2024	4269	969	7542	18	8926	3620	1949	2558	2737	1371	3863	5389	3869	2461	4013	1166	5647
SENP2-3	5065	34	4741	455	2761	977	1993	1857	1279	2499	563	4538	15	4492	757	1576	481	2555	258	2684	1986	2832	708	2687	354	3042
SETD1A-2	455	1	374	52	187	63	147	195	90	191	42	411	2	673	595	187	285	196	107	190	409	257	122	190	38	202
SETD1A-3	6879	29	6452	457	3688	1082	2523	2304	1446	2980	693	5339	56	5545	4256	1813	3045	2803	1529	3749	4492	3217	1753	3165	784	3652
SETD1B-1	1570	4	1416	410	549	851	524	1398	223	1757	100	2790	0	2623	472	1855	250	2529	121	2942	847	2440	355	2479	166	2680
SETD2-2	934	1	742	48	480	198	403	276	194	344	112	614	14	552	1112	227	803	305	264	294	1209	365	382	257	178	352
SETD2-4	10603	29	9949	705	4897	1555	4005	3179	2216	4121	972	7487	29	7541	4246	2189	3632	3462	2023	4434	7128	3560	3276	3758	1599	4514
SETD7-1	453	0	475	36	150	67	164	77	128	177	66	357	2	737	111	107	54	116	18	115	1351	162	515	155	219	172
SETD7-2	2072	10	1972	104	972	265	625	424	345	716	112	1516	10	1903	350	664	245	947	59	724	4612	760	1692	759	943	1062
SETD8-1	3336	14	3756	290	1691	592	1629	1233	811	1653	460	3220	54	3329	2032	1582	1470	2164	583	2361	5702	1506	2413	1801	1448	2191
SETD8-2	4597	7	4170	245	2727	572	2351	1310	1197	1408	416	2207	6	2801	1446	931	810	1322	361	1214	9196	2111	2891	1736	1215	1762
SETDB1-1	255	0	190	18	113	18	71	54	44	67	23	147	2	288	73	50	32	35	15	23	16	37	8	32	5	60
SETDB1-2	150	1	144	12	66	15	64	42	32	44	15	83	1	350	87	18	37	33	10	35	27	58	22	32	13	106
SETDB2-1	941	2	651	92	386	240	265	494	182	650	74	1382	0	1733	250	261	187	438	84	525	515	750	197	772	140	1225
SETDB2-3	1443	3	1327	117	713	303	717	575	350	639	194	1386	4	1891	237	186	230	358	78	346	553	596	183	507	141	664
SETMAR-2	3231	3	2876	202	1501	510	1385	1048	615	1354	324	2805	8	3564	1368	1018	813	1534	247	1111	956	1397	330	1339	144	1516
SFMBT1-3	13070	118	11253	971	6432	2613	4696	5180	2778	6105	912	10287	64	13608	3478	4209	2784	5841	886	5991	2812	6886	896	5311	342	7968
SFMBT1-4	116	1	88	8	79	20	60	56	35	81	13	152	0	161	86	108	32	111	8	89	34	123	19	120	15	135
SIN3A-3	1301	4	1072	94	649	213	550	444	258	453	141	960	5	1166	1217	167	1003	321	482	430	1231	463	558	489	232	568
SIN3B-1	8328	25	6696	582	3818	987	3048	2335	1829	2843	756	5313	79	6361	1459	1910	1083	2568	409	2344	1962	3255	735	2973	380	3552
SIN3B-4	16850	41	13939	1144	8211	2317	6396	4865	3422	6441	1399	11813	47	15018	8723	3711	6835	6284	3085	7357	9215	5763	4195	6845	2189	7460
SIRT1-3	3456	26	2478	279	1593	720	1636	1424	701	1651	314	3458	7	4028	5557	851	4090	1300	1923	1386	3556	1604	1172	1432	745	2227
SIRT2-2	4322	11	3960	543	2138	844	1962	1656	1152	2428	396	4663	19	5016	915	1938	591	2851	287	3084	4256	2503	1956	2569	947	2910
SIRT2-3	7836	15	7604	551	3988	1551	2995	2363	1685	2840	683	5230	9	6198	314	4522	206	5152	185	5066	392	5875	252	5390	184	5127
SIRT3-2	2578	1	1971	153	1064	401	898	795	335	840	141	1774	1	2372	218	673	141	1047	57	1031	314	1158	143	894	72	1397
SIRT3-4	453	0	354	26	153	83	152	137	106	192	47	347	1	390	67	134	27	139	14	127	86	192	34	143	15	132
SIRT4-1	653	2	523	54	260	109	239	167	126	314	71	714	0	1588	82	169	28	192	7	166	32	281	21	142	12	247
SIRT4-3	1541	3	1315	47	788	104	568	192	299	284	160	626	6	1587	956	622	520	719	150	553	545	847	153	588	89	855
SIRT5-1	143	0	140	20	71	27	60	78	38	85	8	167	0	252	289	63	225	80	93	101	140	150	27	133	19	119
SIRT5-2	4245	20	3886	231	2176	752	1702	1559	790	1805	293	3479	10	4063	1798	1295	1352	1939	527	2086	1506	2459	653	2160	232	2759
SIRT6-1	6113	21	4752	389	2727	976	2392	2215	1257	2745	574	5025	32	6998	2486	2959	1350	3759	461	3307	2303	4061	588	2901	468	4791
SIRT6-2	40	0	39	1	18	8	12	11	6	11	4	15	0	30	14	12	0	8	1	2	13	15	4	9	4	18
SIRT7-2	60	4	43	70	30	129	18	298	9	247	3	551	0	1097	71	176	34	325	31	362	96	290	45	397	27	449
SMARCAD1-1	91	1	86	5	33	4	32	33	17	23	12	77	0	57	360	25	224	33	106	42	119	38	54	57	29	56
SMARCAD1-3	874	2	655	72	370	149	273	253	198	358	99	597	6	565	1093	230	680	266	242	276	629	314	145	234	109	359
SMARCAL1-1	4294	7	3716	231	1898	583	1513	1170	824	1736	419	3284	12	3587	1495	1136	980	1930	392	1991	2523	1828	688	1659	450	2287
SMARCAL1-3	1304	2	1305	117	724	218	539	461	290	632	176	1282	4	1214	683	779	332	982	174	1090	1157	1297	427	1106	183	1236
SMYD2-1	12898	27	11172	596	6587	1249	4720	2480	2959	3763	1277	8027	45	9395	7400	1774	6482	3435	3153	4711	10337	2581	6052	3815	3090	4891
SMYD2-3	3718	8	3014	303	1690	555	1711	1244	766	1812	335	2863	13	2710	1768	1055	1399	1702	605	1545	3436	1552	1245	1477	767	2244
SMYD3-6	10038	25	8290	768	4875	1819	4208	2997	2319	4669	1207	9790	32	12687	9857	1809	7792	3017	3527	3172	15386	3157	6176	3351	3360	4880
SMYD3-7	1698	30	1371	117	827	242	641	390	382	553	182	1112	32	1475	1707	349	1035	490	415	452	1754	718	512	659	265	565
SOX2-3	748	2	878	104	415	185	425	326	190	554	100	1114	9	1236	5781	86	4604	114	3408	200	45	1219	20	955	51	925
SOX6-1	17442	140	14597	1523	7704	2806	6104	5197	3812	8025	1653	12919	38	14645	1647	10122	830	11881	363	8305	1149	12998	494	8893	128	10512
SOX6-2	7653	27	6910	529	3718	1071	2921	2490	1789	3246	905	5996	4	6374	267	2342	302	3618	71	3518	252	3238	139	3119	71	4033
SSRP1-3	5908	20	5076	545	2882	1136	2007	2288	1327	3511	614	5797	39	6385	17448	763	17503	1455	9812	1931	13375	2838	5763	3033	3514	4737
SSRP1-4	4525	18	3737	465	2181	985	1690	2079	876	2430	417	4845	24	5575	7777	496	7260	1289	3793	1412	5418	1826	2089	2072	1164	3036
STAG1-1	14915	25	13638	494	8611	1167	7313	2635	3669	3715	1521	6363	112	9234	9617	2535	6997	3774	2919	4332	22250	2985	9173	2738	6973	5093
STAG1-3	18801	41	18178	1064	8610	2618	7379	4971	4355	7584	2130	12740	30	17882	12343	6197	10527	10346	4007	11289	26656	3971	13985	6275	9173	9690
STAT3-2	5767	10	4275	378	2229	871	2139	1933	1058	2106	469	3773	11	5053	7631	935	6022	1476	2971	1753	14201	1158	6033	1353	4493	2229
STAT3-4	30263	204	25648	2838	13532	6500	10677	11128	6668	16894	3048	26046	145	28169	34598	4416	32380	8326	17206	11548	47816	4533	25921	6723	22195	12244
SUDS3-1	3	0	0	0	0	0	6	0	0	0	0	0	0	2	0	0	0	0	0	1	5	0	0	0	0	0
SUDS3-3	1784	2	1517	38	746	92	464	177	215	280	59	609	5	867	1900	158	1482	296	609	434	2193	403	847	659	289	617
SUV39H1-2	1727	18	1590	421	635	783	492	1269	192	1444	100	2408	2	3008	436	1178	327	1664	152	1985	178	2062	104	2420	31	2177
SUV39H2-2	2776	4	2307	231	1054	387	850	852	571	1119	251	2165	3	3645	5425	792	3535	1007	1233	874	703	1053	271	823	143	1200
SUV420H1-2	15452	338	13512	939	7305	2376	5502	5111	3412	7424	1699	12614	107	16386	14761	3256	13079	5463	6105	6624	14331	5681	6155	6770	4034	9601
SUZ12-2	12394	13	10947	721	5857	2391	4525	4890	2461	7659	947	12923	24	19078	21672	1343	18551	2468	9406	3629	14654	3392	5874	4176	4323	7481
SUZ12-3	3287	7	2880	206	1472	515	1108	1029	573	1576	273	2765	20	2644	12701	739	11165	1571	4969	1902	6309	1467	2930	1936	1372	1955
TBX3-2	3206	6	2783	197	1383	612	1193	1098	591	1549	297	2593	10	3684	103	1253	66	1716	29	1389	9159	1556	3037	1299	1823	1913

TBX3-3	1362	0	995	17	538	46	503	99	258	118	96	191	1	217	21	26	2	31	8	61	5355	141	2203	95	1435	244
TCF3-1	2208	18	1935	259	1035	636	728	1286	423	1581	212	2950	12	2771	602	590	572	949	206	1207	351	1332	190	1532	85	1763
TET1-1	9675	23	7633	645	4306	1324	3498	2806	2024	3303	803	5917	32	8283	8475	2104	6555	2953	2716	3034	3410	4223	1089	3367	559	4278
TET2-1	2474	2	2064	156	1114	377	888	739	418	779	178	1608	10	2087	383	612	199	922	123	933	1004	949	374	954	218	1264
TET2-3	20504	662	18429	1941	9022	4208	7890	7318	4949	10609	2647	20501	34	22113	5793	10516	4015	13124	1629	13142	12214	12155	4764	11729	2565	14884
TET3-1	485	1	442	38	245	95	154	172	118	268	40	384	6	457	185	169	107	222	45	159	151	234	59	196	36	196
TET3-6	15935	70	12135	1079	7725	3110	6311	6941	3737	9250	1541	16261	17	16254	2976	4996	2025	7430	740	6661	2933	7229	792	5252	406	7551
TK1-1	1575	6	1182	157	693	274	534	492	257	566	127	1112	3	1594	1399	451	1051	784	424	721	440	746	150	660	93	1009
TK1-2	20810	1520	18537	1875	14076	1691	15501	2369	5254	8633	2818	19601	2463	24442	36151	2379	33029	4283	15871	5127	17290	10828	4915	8673	3030	13221
TP53-2	917	1	909	11	540	45	408	47	187	77	61	69	1	59	109	15	60	6	32	13	730	17	174	18	68	15
TP53BP1-4	3558	3	3151	300	1745	653	1362	1218	859	1947	374	3468	4	4620	6139	868	4720	1218	2133	1488	5416	1924	1727	1659	1003	2366
TP53BP1-7	15471	114	12982	1231	7030	2568	6030	4956	3499	6472	1494	11681	42	14197	17499	1622	16312	3199	9664	4145	16784	4533	7795	5049	5415	7945
TRDMT1-2	20285	1602	17477	2816	8390	3555	6682	7103	3774	10383	1462	18756	47	18640	3186	8986	2130	12416	1046	15067	3608	16251	1257	16761	414	16967
TRDMT1-4	6062	40	5384	745	2601	1320	1973	2858	968	3276	371	6608	8	7915	1684	3085	974	4099	326	3720	646	4565	255	3662	117	4818
UHRF1-1	1541	12	1746	90	772	252	513	558	214	912	67	1835	8	2796	6614	250	5808	502	2662	802	1060	1447	359	1929	191	2136
UHRF1-2	1905	2	1864	122	976	309	765	636	418	680	199	1620	2	1792	1139	231	1014	535	519	668	385	702	162	772	64	799
VPS72-1	983	2	721	51	407	115	298	230	111	304	78	523	0	1354	1616	184	1268	269	432	295	471	328	195	274	124	409
VPS72-2	1286	9	1288	112	589	187	404	389	251	682	143	1102	0	2231	2592	360	1824	520	1214	887	1500	900	639	1050	245	985
WDR5-1	2107	10	1676	195	969	519	692	1228	389	1616	126	2778	17	4316	1596	1349	763	1578	319	1692	1002	1888	266	1485	120	1969
WHSC1-2	403	24	318	60	187	109	183	188	97	223	40	344	1	467	493	234	330	350	136	419	208	513	74	370	24	387
WHSC1-3	383	1	330	36	154	82	182	96	59	134	28	218	1	357	873	63	574	134	275	159	298	112	123	124	57	128
WHSC1L1-1	2173	4	2174	278	1054	517	627	792	355	1063	179	1957	8	1963	3440	470	3314	1059	1771	1556	5349	1134	3311	1886	1255	1595
WHSC1L1-2	923	2	824	73	470	114	323	216	208	446	106	1008	0	1277	1414	123	855	227	447	272	2244	319	777	303	398	413
WNT8A-2	3619	8	3439	379	1669	710	1440	1429	750	2023	386	3863	2	4155	103	2659	101	3227	54	3168	68	3565	74	2702	5	3745
YAF2-2	4555	14	4410	591	2075	1317	1631	2273	975	3383	314	5753	3	6685	2127	1951	1589	3124	618	3665	4751	3294	1972	3985	668	3530
YAF2-3	1915	2	1726	104	873	181	649	570	408	597	159	1245	6	1893	765	257	567	599	200	585	2110	723	720	641	350	952
YAF2-5	6588	103	6660	598	2855	1306	2126	2445	1071	2983	398	5664	19	7736	3267	1552	2708	3476	1277	3863	6185	1903	3253	3258	1835	4616
YY1-2	1287	2	954	75	527	163	516	276	231	489	116	787	1	1492	865	174	668	240	368	358	752	283	243	274	297	670
ZNF317-2	1062	43	665	136	573	200	385	438	210	522	124	1212	0	1303	41	130	38	228	29	325	144	467	39	393	16	569
ZNF317-3	1795	9	1326	117	823	271	592	520	294	695	124	1362	6	2106	560	528	364	719	105	743	479	838	188	943	124	1149

Amplicon	REP2																									
	G10		G9		G7		G5		G3		G1		G0		I1		I2		I3		F1		F2		F3	
	HS	PA	HS	PA	HS	PA	HS	PA	HS	PA	HS	PA	HS	PA	HS	PA	HS	PA	HS	PA	HS	PA	HS	PA	HS	PA
ACTL6A-1	126	5	116	8	61	5	78	39	68	44	17	73	14	149	413	11	315	15	326	31	119	17	92	20	56	23
ACTL6A-2	983	3	984	66	674	143	809	384	573	631	138	731	6	617	360	91	389	170	495	335	251	155	183	198	139	289
AEBP2-1	2239	15	520	51	84	44	223	156	73	150	37	282	19	358	228	22	157	31	55	34	35	46	19	39	29	39
ARF3-3	111	1	108	12	48	14	54	22	61	41	13	30	6	90	30	6	35	3	52	4	75	12	37	8	31	25
ARF4-4	4242	6	4674	30	2927	75	1730	207	1098	284	302	555	144	902	39084	28	27255	46	23530	101	47812	81	42099	81	42253	205
ARF5-1	25825	84	18692	784	13351	1985	13142	5841	9614	10070	3387	17162	123	19528	22516	308	27057	641	39739	2003	18667	504	24653	1512	17471	2073
ARF5-2	1160	15	1121	98	616	172	550	531	387	714	111	1008	36	1333	4490	97	3734	132	3865	276	2456	93	1713	228	1471	365
ARF6-3	939	10	824	39	478	123	418	321	315	439	102	669	55	1002	6527	151	3687	135	2174	199	6684	162	3883	197	2236	198
ARID4B-1	534	4	681	57	523	161	493	307	244	447	82	646	10	773	675	62	520	102	595	212	851	153	470	210	337	278
ARID4B-2	2665	10	2568	93	1608	293	1147	579	667	602	178	1159	44	1625	1420	105	886	103	973	233	1572	163	698	199	677	377
ASF1A-1	8	0	3	0	5	0	0	8	1	0	1	4	1	3	4	0	0	2	0	2	0	0	1	1	0	0
ASF1A-2	536	6	153	10	41	22	62	101	54	140	10	162	3	255	123	12	55	41	22	31	23	16	8	33	6	24
ASH1L-4	361	1	341	20	185	46	189	158	107	170	39	235	4	418	226	61	132	95	78	112	193	48	104	86	65	87
ASH1L-5	193	6	216	3	131	55	153	105	80	122	26	193	11	215	192	16	48	20	69	43	225	33	115	34	120	78
ASXL1-2	28579	162	30513	1334	20210	4203	15244	10795	7589	18568	2078	26396	675	31369	6608	3255	4879	6005	4551	13508	15864	4134	10575	7423	7396	13649
ASXL1-3	2392	38	2369	162	1479	572	1161	912	807	1805	178	2511	11	2884	699	378	490	698	404	1036	1181	355	1085	673	617	1043
AXIN2-1	1078	15	910	49	754	129	592	300	403	534	107	747	7	1255	794	178	540	326	487	567	315	294	194	487	90	545
BAP1-3	1317	23	1244	242	674	528	654	1034	369	1507	106	1669	18	1883	1211	460	877	637	984	1535	763	704	514	938	257	1144
BMI1-1	40	0	21	1	28	9	23	21	39	35	15	15	9	44	1	0	1	0	2	4	58	5	38	10	24	13
BMI1-2	5311	32	4621	114	3152	346	2139	685	1593	1127	323	2007	47	3538	988	117	602	175	781	384	6533	224	6660	336	4361	536
BRCA1-1	2974	22	3155	198	1829	642	1863	1683	1340	2340	404	3670	10	5398	1395	219	1091	337	1349	788	170	330	133	452	126	820
BRCA1-2	287	3	204	11	151	41	197	102	116	150	33	145	13	200	209	25	147	31	181	99	33	51	22	34	12	120
BRCA2-2	22	1	24	0	21	2	46	22	16	19	6	11	0	19	30	2	33	4	12	2	0	2	0	1	4	0
BRCA2-3	19958	90	21750	736	13640	2713	12014	5319	6142	8217	1773	12221	69	17835	17324	1513	11049	2283	9632	3185	1816	1802	965	2960	687	5027
BRMS1-2	3103	20	3074	226	1806	606	1515	989	893	1288	210	1750	8	1580	1219	361	1132	475	1310	1105	1203	503	858	767	711	1114
BRMS1-3	113	3	150	17	108	34	107	97	34	149	20	185	4	213	28	12	15	33	26	87	22	34	28	65	24	109
CARM1-1	1801	4	1719	57	477	104	756	454	400	564	130	785	8	1059	4559	170	2502	220	880	142	1360	169	681	177	231	175
CBX2-1	341	4	353	23	220	30	244	126	201	290	55	496	1	498	526	90	451	149	583	400	11	145	5	341	3	242
CBX2-2	1260	9	1387	86	719	364	644	849	364	1269	101	1724	10	1805	1368	340	770	427	846	883	6	266	8	531	6	553
CBX4-2	34	3	40	17	12	46	17	167	28	246	9	385	6	388	56	74	63	160	71	329	196	100	187	220	61	235
CBX4-3	3049	13	4119	209	2877	771	2087	1414	1502	1515	345	3023	10	2994	1710	673	770	688	952	1197	2515	427	1929	739	1549	1006
CBX5-1	53	1	31	8	24	14	38	13	36	17	6	28	7	58	362	8	245	9	432	26	108	12	43	15	39	7
CBX5-2	1720	14	2116	209	1385	380	1066	839	626	1346	165	2111	20	2983	6693	106	6135	178	6315	471	2790	313	1991	424	1298	656
CBX6-3	28196	111	29510	1530	18389	5443	16190	10679	9044	16798	2917	23080	198	20351	10474	2389	8762	3876	7965	7328	18986	1715	17745	3760	15600	6795
CBX6-4	1740	12	1482	145	887	304	882	734	419	1090	111	1410	13	2714	146	113	80	127	91	225	745	117	462	215	252	404
CBX7-3	777	20	567	62	452	213	484	444	295	788	72	903	15	1279	17	64	23	94	14	244	143	106	116	193	80	289
CBX8-1	598	9	528	46	389	70	342	226	253	325	63	335	4	472	35	55	34	79	23	219	68	67	44	91	39	197
CBX8-3	629	1	468	25	380	32	316	134	244	194	98	239	253	202	77	16	63	57	76	90	352	81	249	50	111	135
CDK4-2	1519	27	1414	153	974	331	693	672	497	1230	193	1637	15	4489	1268	205	623	205	532	306	2938	393	1517	402	903	645
CDK4-3	152	6	132	12	78	12	86	69	76	99	20	91	11	145	138	12	96	21	91	60	448	38	281	54	127	60
CDX2-1	8674	114	7989	1055	4646	2863	3973	5788	2293	7484	753	9766	38	11891	288	5672	264	5780	239	9357	209	5062	83	6473	68	8245
CDX2-4	3672	29	3613	101	1867	408	1720	1074	841	1725	210	2160	3	2580	88	991	45	1006	25	1421	23	741	25	1116	1	858
CHAF1A-1	2879	36	2913	182	1580	364	1672	1119	1173	1832	266	2453	26	3295	1692	184	1500	310	1719	706	224	381	156	574	94	926
CHAF1A-3	201	2	226	12	135	39	175	121	96	146	35	212	6	209	359	29	295	45	417	125	42	27	36	51	19	84

CHD3-1	171	1	112	8	84	36	76	42	68	51	13	58	10	110	221	28	91	28	84	47	627	25	351	49	257	62
CHD3-2	36	0	22	4	22	8	15	29	5	38	3	76	1	124	14	14	12	5	1	11	20	14	3	20	4	29
CHD4-4	727	6	189	9	65	24	107	63	71	95	6	98	12	220	1118	36	724	34	351	43	352	24	150	30	112	27
CIITA-3	1317	22	1324	43	776	164	755	440	368	562	83	751	5	1110	7	18	1	43	2	140	7	70	2	74	10	216
CIITA-4	7035	36	6955	651	5083	1601	4613	3322	3072	5058	971	7064	20	7920	46	594	64	1252	66	2093	78	1093	37	1595	28	2082
CREBBP-1	59948	338	57687	4625	39721	14348	29527	29166	16662	43670	6570	60276	480	70335	40989	4800	37386	7308	40617	18359	40929	4960	43360	10022	30838	17144
CREBBP-3	84	2	79	6	64	5	65	45	49	75	8	115	2	127	45	7	67	5	90	42	92	26	90	19	71	57
CTCF-5	2201	31	1867	134	1353	404	1278	857	910	1749	205	2155	24	2559	3413	335	3260	515	3577	1352	1914	592	1697	913	838	1179
CTCF-6	20	0	16	2	14	3	16	7	12	11	6	17	2	30	9	1	14	2	7	4	4	6	7	4	1	0
DGCR8-1	4565	25	5032	318	2923	1022	2285	1934	1375	2566	344	3484	10	4834	2182	654	1046	781	733	1038	2204	1014	1177	1266	484	1153
DGCR8-2	5030	36	5559	558	3628	1354	3137	2435	1518	3638	697	5098	26	6199	2063	1101	1349	1293	1359	2750	1908	1516	1360	1988	1019	3116
DICER1-1	3442	32	3933	271	2491	626	2534	1446	1605	2549	462	3664	13	4863	3119	284	2586	430	1752	621	2342	398	1585	662	1099	940
DICER1-5	6964	62	7002	416	4491	1301	4014	2513	2554	4041	746	5026	61	6099	5699	528	5186	961	5477	2101	5235	1137	4368	1940	3003	3010
DMAP1-1	18464	137	19164	1717	13518	4965	9457	8619	5289	13085	2062	20927	68	32212	15296	2224	10499	3005	10219	5720	6239	2637	4908	5404	3268	7981
DMAP1-2	13150	104	13265	1195	8715	4032	8230	8562	4961	12660	1775	16170	53	18517	7978	1994	7165	3240	9080	7997	5666	3604	4220	5987	2837	7399
DNMT1-4	13232	61	15359	960	11414	2273	10153	5951	6730	8579	2171	12378	55	13459	17770	952	16630	1647	20280	4605	8617	2417	7373	4802	4647	6630
DNMT1-6	4459	52	3565	274	2458	970	2574	2213	1653	3692	467	4362	29	4991	4394	698	3342	1084	3781	2126	1007	768	820	1272	366	1676
DNMT3A-2	2987	30	2545	163	1618	413	1442	1011	988	1958	243	2483	23	3507	94	145	74	277	72	554	201	239	129	403	109	511
DNMT3A-3	824	10	765	60	497	110	517	290	262	443	78	620	7	814	43	63	35	75	43	157	112	149	65	197	57	202
DNMT3A-4	10	1	2	0	10	2	11	4	1	18	1	26	0	20	0	2	0	1	1	5	1	0	0	4	1	9
DNMT3B-3	1937	21	2364	187	1582	541	1462	1181	786	1436	273	1876	22	1818	16965	176	13233	328	9423	466	28	133	21	133	18	110
DNMT3L-1	168	7	185	25	101	40	90	86	55	101	28	101	6	174	7	52	2	34	2	89	12	77	0	50	1	74
DNMT3L-3	1029	12	888	103	516	357	477	730	302	1058	76	1185	3	1541	10	282	11	538	13	1003	14	502	3	774	9	820
DOT1L-3	265	1	223	11	210	47	154	99	122	157	34	175	1	211	45	14	40	22	33	30	15	2	6	7	5	7
DOT1L-5	2172	70	2306	84	1879	389	1157	838	756	1144	259	1748	61	2627	1774	192	1289	328	1120	446	715	357	416	464	263	681
EHMT1-1	916	7	997	56	510	173	657	471	506	831	155	972	6	1245	886	62	974	113	1163	294	552	172	340	266	209	337
EHMT1-2	118	2	138	8	55	27	62	40	88	110	8	134	1	182	87	9	87	17	139	59	49	28	24	23	42	53
EHMT2-2	33	0	55	12	42	15	42	47	38	67	6	51	1	115	51	5	22	12	23	13	6	9	24	14	5	17
EHMT2-3	5284	21	4555	231	3404	617	2699	1318	1991	2368	801	3270	15	4411	3865	317	3125	600	4213	1311	1410	587	1580	1215	649	1293
EIF2C1-1	2884	30	3299	198	1842	466	1996	1289	1156	1987	298	2401	29	2774	3809	272	3147	491	3182	1017	2529	596	1645	891	996	1027
EIF2C1-4	1503	16	1675	129	946	359	886	704	517	919	190	1276	21	2161	1167	95	1077	198	1379	535	1073	365	688	431	599	854
EIF2C2-3	97	2	170	3	84	25	49	33	56	48	5	75	2	102	363	43	198	61	239	45	57	21	33	21	27	28
EIF2C3-1	315	6	277	32	181	48	243	136	124	148	15	177	5	301	100	12	95	19	113	52	74	27	33	13	19	37
EIF2C3-2	3796	11	3874	149	2442	508	2176	1083	1330	1745	414	2691	17	2859	1996	140	1490	215	1345	413	1516	257	876	419	603	659
EIF2C4-1	32	0	21	1	15	12	10	17	13	30	1	29	0	20	1	2	3	5	1	3	3	5	4	3	1	2
EIF2C4-4	4247	116	4141	276	2639	965	1904	1989	1256	2916	328	3719	12	5152	697	326	688	543	616	1037	894	583	625	795	388	1065
ELP3-3	6	2	11	0	4	7	4	3	10	10	3	16	1	29	10	4	2	4	2	1	6	2	0	4	1	1
ELP3-4	33	0	31	2	21	12	12	10	8	18	2	38	0	63	18	3	17	13	7	10	17	4	8	5	5	10
EP300-1	2000	17	1723	57	1160	181	1048	515	598	612	183	1017	19	2137	1053	62	762	55	804	165	697	66	441	127	421	209
EP300-2	1714	8	1560	62	1137	122	1009	292	623	407	229	484	112	611	2092	54	1813	127	2024	254	1558	86	1144	131	802	190
EP400-2	3751	24	4097	261	2433	788	2060	1833	1086	2693	349	4059	19	5632	1551	219	1004	384	1066	695	1187	413	787	655	634	1165
ERAS-2	1749	14	1901	208	1121	920	733	2186	483	2472	135	4024	1	4947	37	557	24	884	15	1304	15	783	10	978	6	1336
ERAS-3	200	4	239	12	101	53	177	149	77	254	53	308	3	368	16	152	11	168	14	383	7	173	8	231	0	283
ERCC6L-1	546	27	470	122	357	257	450	790	334	1497	95	1625	16	2234	1437	222	1208	510	1245	831	70	299	57	505	28	598
ERCC6L-3	34	2	41	8	27	42	31	80	13	77	5	124	1	208	141	48	69	75	59	49	2	36	3	56	7	48
EZH1-2	1702	18	1782	117	1158	310	1164	756	842	1108	203	1388	91	1758	227	236	218	414	243	1145	609	343	506	647	428	857
EZH1-4	1711	14	1686	123	1016	259	920	707	686	1029	239	1631	15	1833	509	359	384	557	411	977	591	385	533	541	379	654
EZH2-1	1203	13	1059	73	633	208	544	481	446	910	141	1386	7	1967	844	57	607	70	633	203	19	48	6	54	13	44
EZH2-3	2017	28	1997	176	1355	444	1058	1004	647	1275	158	1645	29	1975	2310	153	1881	231	1889	471	173	182	72	191	64	436
GATAD2A-1	640	8	645	41	350	122	353	250	231	417	78	552	13	677	470	29	458	65	428	102	285	72	218	80	143	130
GATAD2A-3	34416	190	38911	3021	25092	7709	19862	16266	12181	23818	3831	33540	283	35974	27984	2105	24221	3556	30291	8374	19463	4292	15802	8510	16276	15734
GSG2-2	4854	29	5004	303	3385	1164	1996	2167	1269	2763	398	4264	2	5592	2072	734	1155	870	670	833	102	609	69	846	18	844
GSG2-3	23	0	15	4	2	0	9	4	1	7	6	6	0	2	13	0	6	0	6	6	0	0	0	0	0	1
GTPBP1-1	4504	44	4385	245	2620	759	1929	1449	1321	3334	361	5742	42	6869	3320	279	2388	463	2539	1268	1295	537	929	979	442	1663
HAT1	124	0	97	6	66	10	67	27	36	52	2	60	1	98	258	3	164	1	242	22	134	9	70	10	29	12
HCFC1-3	401	26	373	69	328	234	291	770	193	998	77	1792	41	3085	1799	98	1526	236	1740	466	992	208	435	260	392	471
HDAC1-2	140	2	120	7	58	22	102	70	72	144	30	149	3	211	205	9	202	14	283	44	181	11	162	35	141	71
HDAC1-3	8518	52	7886	630	5432	2015	4575	4092	2820	5273	1161	7876	176	9314	6694	318	5436	628	6627	1204	4825	487	3407	651	2941	1306
HDAC10-1	1022	4	872	15	629	77	446	182	202	312	30	358	1	560	27	18	34	54	37	93	47	51	42	61	19	83
HDAC11-1	585	5	501	45	341	144	299	289	165	455	32	625	3	662	44	119	18	181	41	334	53	147	29	227	13	364
HDAC11-2	266	3	220	20	135	60	135	110	75	133	24	216	3	244	36	67	18	73	7	198	23	86	20	82	9	96
HDAC3-1	336	10	358	31	190	108	201	249	127	301	31	487	16	671	1107	72	848	128	727	209	728	172	441	213	198	268
HDAC3-3	5815	34	5289	257	4184	1147	3561	2042	2212	3187	610	4063	11	4798	4067	323	2929	440	2072	807	1962	385	1293	571	500	473
HDAC4-1	413	16	364	51	233	110	220	293	179	547	54	484	11	648	57	36	67	65	113	204	99	107	79	127	71	224
HDAC4-2	0	0	3	1	3	1	3	2	2	2	1	4	1	8	7	0	1	1	0	0	1	2	2	0	0	1
HDAC5-1	209	1	202	10	124	72	136	117	41	111	38	167	0	384	7	13	0	20	7	38	6	20	7	36	4	37
HDAC5-3	97	0	81	4	60	3	62	11	39	50	5	32	2	67	8	2	9	4	7	20	11	5	5	8	0	1
HDAC6-1	48	2	35	9	15	17	8	44	15	66	4	47	0	88	22	7	23	13	23	34	13	8	6	19	4	20
HDAC6-2	36	1	15	0	15	23	39	19	19	42	1	57	0	161	60	7	11	1	22	7	11	5	1	3	1	2
HDAC7-2	1612	16	1917	137	1206	360	1235	992	1003	1695	285	2390	3	2459	630	347	600	608	756	1551	873	401	666	873	474	1139
HDAC8-1	2769	14	2348	75	1367	177	1056	361	593	725	216	873	9	1392	52	50	86	98	77	253	22	93	21	128	28	270
HDAC8-3	7462	73	7530	962	4501	2499	4125	5883	2463	7955	684	11550	48	13408	4424	1125	3821	2149	3847	3969	3904	1926	3003	2745	2125	4072
HDAC9-1	1276	10	1227	70	694	200	740	494	517	845	133	1142	14	1305	631	177	489	247	545	680	242	297	202	486	140	675
HDAC9-3	2	1	8	1	1	0	0	0	0	0	0	15	0	0	0	0	0	0	0	0	0	0	0	6	0	0
HESX1-1	795	7	766	43	605	142	456	331	242	490	99	556	1	576	126	39	75	67	58	49	2	30	1	47	3	23
HESX1-2	158	0	146	15	72	39	60	77	55	108	19	179	1	223	14	14	3	15	13	16	1	13	0	10	0	45
HIF1A-1	40	0	50	5	40	13	40	28	97	38	39	21	33	37	476	17	477	13	404	30	1316	14	623	12	478	23
HIF1A-2	1010	5	1157	83	676	192	561	485	406	579	140	941	54	1157	3904	77	2987	101	2727	156	5203	74	4182	74	3060	147
HOXA1-2	6214	62	6645	326	4344	1191	4138	2142	2292	3435	699	5272	25	6843	323	1073	177	1528	159	2491	336	1030	258	1934	81	2922
HOXA1-5	475	7	438	39	241	93	210	273	152	361	42	448	5	541	16	238	14	247	19	383	26	218	5	259	2	234

HOXB1-3	3040	8	2689	169	1675	416	1507	847	797	1167	337	1946	6	1949	206	938	35	686	65	1457	24	571	33	901	2	691
ING1-4	587	10	734	69	493	232	373	524	257	716	79	1129	4	1155	577	94	408	234	279	311	103	179	76	220	34	259
INO80-1	592	12	457	31	315	91	290	188	205	318	53	452	6	757	69	35	66	64	61	138	134	85	72	111	55	174
INO80-2	3928	178	2644	4733	1751	12036	1039	13849	439	13817	173	17228	11	20137	334	4650	120	5423	127	7094	340	5460	208	7895	226	12583
JARID2-3	4471	42	5015	410	2962	1202	2295	2756	1597	4269	602	6104	81	8724	9562	319	7050	558	8668	1234	686	707	302	694	271	1686
JARID2-5	10720	72	9654	513	7297	1761	5645	3420	4017	6969	1117	11904	56	11007	29514	587	29681	908	32533	2667	2909	1225	2286	3046	1046	2577
JHDM1D-1	508	15	407	71	314	153	288	331	141	489	66	584	4	932	205	112	215	171	199	392	120	152	68	234	58	369
JHDM1D-2	88	0	86	9	67	19	38	40	32	38	5	75	5	87	16	7	16	11	25	22	12	9	5	8	4	10
JMJD4-1	1856	19	1599	91	1049	399	1160	768	641	927	167	1353	8	2313	429	388	291	577	254	1073	300	663	182	1134	158	1438
JMJD4-2	16123	66	16880	808	13767	2182	11018	4481	5438	6495	1805	11367	11	13108	2331	887	1065	1335	568	1330	1265	947	700	1698	141	687
JMJD6-2	1855	14	2173	142	1656	425	1319	920	766	1344	200	1651	11	1586	425	175	222	170	136	150	428	169	213	168	55	105
JMJD7-2	1292	4	1301	38	823	174	722	324	536	498	133	589	3	989	83	27	50	29	68	99	40	18	27	29	12	49
JMJD7-3	108	3	80	14	64	29	79	74	48	122	10	111	1	164	15	11	9	12	13	28	13	21	1	28	3	45
JMJD8-1	321	3	387	22	270	50	183	142	174	269	38	280	2	590	440	18	243	24	326	70	460	10	269	24	242	56
JMJD8-2	587	19	645	111	351	187	348	437	230	810	84	1225	53	1633	1353	204	797	269	746	506	2157	266	1203	348	972	694
KANSL1-5	57916	55	62161	425	50798	1307	28363	2277	12764	2349	3250	4497	77	4806	38455	432	18880	362	7286	356	28115	427	15861	532	3508	280
KAT2A-1	5208	38	5070	249	3460	635	2479	1294	1675	2188	591	3025	18	5103	3994	499	2561	696	1437	890	2198	662	1584	925	768	960
KAT2A-3	276	4	171	12	114	21	65	47	65	74	22	167	4	564	66	17	56	24	43	68	41	23	14	20	16	28
KAT2B-2	1318	11	1216	44	935	154	835	519	461	681	156	748	4	1282	63	104	61	197	29	388	473	198	305	315	107	346
KAT5-1	101	7	80	9	63	21	80	57	50	79	13	115	1	131	37	7	31	18	16	21	30	21	16	30	18	23
KAT6A-1	1287	8	1191	56	868	169	812	468	495	643	162	1003	0	1302	458	74	544	164	524	371	700	152	699	278	354	355
KAT6A-2	855	13	793	62	443	148	428	309	365	638	64	796	9	903	238	67	286	110	361	316	360	96	328	176	243	371
KAT6B-2	18	1	23	0	13	1	9	16	15	16	2	13	3	24	17	10	9	6	7	5	12	2	2	7	8	25
KAT6B-3	9021	47	8867	578	6023	1457	4682	3114	3069	4692	1009	6299	14	7128	3287	927	2392	1402	1772	1991	5228	981	3740	1562	2297	2458
KAT7-1	2493	9	2462	112	1551	367	1599	992	793	1289	235	2037	13	2642	1744	105	1405	195	1802	395	1309	181	945	281	663	651
KAT7-2	67794	288	69824	4615	44721	14534	27619	22489	12135	31454	3780	56482	140	71407	61510	4974	42198	7273	36946	14564	36574	9396	28938	18227	19135	29616
KAT8-1	68	4	31	2	48	17	40	27	37	38	6	74	1	182	14	2	4	3	19	5	7	5	13	3	6	7
KCTD3-1	100	6	111	6	62	24	55	28	87	79	31	56	20	119	730	21	537	24	477	41	260	22	95	19	99	36
KCTD3-2	706	9	904	44	464	148	339	258	268	465	114	539	8	557	1979	116	1497	116	1145	279	933	119	483	149	229	168
KDM1A-1	11475	71	10882	567	7173	1502	6550	3547	3913	5773	1222	9035	50	11310	23851	438	21503	738	25958	1939	10399	1739	9306	2662	6554	4640
KDM1A-3	20	1	7	1	13	2	10	6	7	10	2	6	7	24	222	5	122	4	134	3	8	1	8	2	4	2
KDM1B-2	420	8	375	26	222	71	237	224	154	258	40	314	5	554	26	27	29	17	43	44	47	27	32	23	38	59
KDM1B-3	273	8	273	17	202	34	221	100	101	136	26	153	5	291	22	8	37	35	47	74	88	40	81	89	69	107
KDM2A-1	16	1	22	4	8	5	3	9	10	9	0	4	1	11	4	0	3	3	4	4	26	1	18	4	7	6
KDM2A-2	1506	30	1420	136	1023	333	852	677	577	1055	204	1360	13	1847	644	132	512	320	480	597	926	226	611	411	388	487
KDM2B-1	219	4	249	20	140	42	158	42	107	74	29	111	19	117	797	18	758	32	541	47	104	22	70	36	46	32
KDM3A-1	554	3	551	32	386	66	341	266	225	349	89	543	5	630	638	52	399	112	283	163	360	108	195	100	104	96
KDM3A-3	5604	18	1076	90	364	118	617	519	281	631	133	812	10	1357	1058	152	685	176	364	169	430	121	259	176	89	135
KDM3B-2	1050	21	508	49	327	104	265	292	217	346	76	421	28	764	494	32	474	41	471	104	352	57	315	115	198	157
KDM3B-3	693	16	478	33	259	97	297	173	137	335	27	542	5	1201	1261	161	552	131	427	309	189	59	102	130	59	165
KDM4A-1	304	4	38	6	39	30	36	55	30	105	10	128	2	184	124	12	109	31	54	43	33	9	14	12	13	34
KDM4A-3	33	0	22	2	20	1	19	5	10	22	1	24	0	36	88	4	53	6	75	23	11	0	6	6	4	8
KDM4B-1	418	3	380	38	309	91	242	220	181	227	40	476	2	587	77	68	79	132	89	370	84	154	81	233	79	348
KDM4B-2	774	12	618	32	357	84	368	264	234	466	45	486	13	618	372	68	222	109	208	199	182	66	148	138	105	154
KDM4C-4	773	5	717	62	475	152	411	269	244	418	65	568	3	919	143	38	117	49	94	108	317	139	112	112	122	196
KDM4D-1	2083	23	1885	88	1396	526	1370	1391	708	1926	181	3498	12	4524	61	417	90	649	53	1352	13	610	5	991	8	1214
KDM4D-4	443	18	323	36	163	142	182	327	129	505	20	690	0	682	33	394	14	420	17	923	7	401	7	507	2	323
KDM5A-1	378	5	270	18	169	105	132	125	79	164	36	237	5	501	566	96	217	83	204	157	195	54	134	104	60	113
KDM5A-3	53	2	63	4	48	15	29	61	36	45	8	29	3	65	34	5	32	7	45	21	45	10	25	24	17	28
KDM5B-1	12537	101	12284	1080	8458	3522	6164	5935	3751	8467	1030	12375	101	14914	21187	602	19101	1112	21599	2359	11642	1521	8151	2386	6209	3825
KDM5B-3	371	18	274	38	216	76	203	188	138	203	45	206	37	450	370	14	368	39	478	54	266	47	190	57	173	87
KDM6A-1	9	0	0	2	10	1	3	11	4	25	1	4	3	58	41	17	33	13	12	16	13	15	17	7	2	14
KDM6A-2	2250	93	2917	699	1795	1497	1680	3123	1050	4874	351	6469	30	7983	6846	865	5612	1193	7169	2912	3748	1959	2310	2257	1700	3074
KDM6B-4	221	5	248	22	129	53	140	99	147	220	42	281	10	325	600	152	254	195	297	320	797	154	362	134	161	168
KDM8-1	299	18	296	70	121	147	184	323	59	375	19	651	3	857	24	115	29	248	15	496	31	200	5	354	14	519
KDM8-4	96	4	107	29	77	60	93	174	45	270	10	250	2	352	11	11	5	20	0	32	3	28	4	23	2	24
LBR-1	24	3	45	4	11	9	38	9	18	38	6	45	4	37	98	1	83	2	109	13	24	3	9	1	13	5
LBR-3	257	3	351	16	201	75	245	236	202	378	42	498	9	642	563	25	742	67	998	151	140	74	152	112	113	204
LEFTY2-3	33	1	55	7	22	19	27	25	15	32	10	37	0	79	5	8	1	13	3	15	2	1	0	1	0	6
LEFTY2-4	1351	13	1525	71	860	212	823	750	583	1140	129	1608	3	2107	424	144	275	277	270	539	4	194	2	436	2	723
LMNA-7	38551	224	35412	2657	27161	7116	23819	15223	19012	25068	4947	37434	223	41704	24632	1892	21165	3409	27685	9660	46833	1137	47764	1772	42901	3444
LMNA-8	2886	17	2789	176	1886	634	1321	1078	838	1406	281	1975	92	2531	3981	311	2847	383	2952	624	13550	274	10225	473	9269	717
LMNB1-2	1563	3	1945	106	1190	181	890	244	462	429	163	534	28	671	4067	57	2946	66	2888	157	119	67	48	71	88	158
LMNB1-3	3209	25	3407	80	2233	298	1779	647	1458	940	840	1429	57	1794	5498	65	4965	119	6428	317	600	188	524	288	302	437
LMNB2-1	21162	234	20222	1154	13445	3311	12046	6980	6142	12149	1937	17067	86	18445	19876	973	17703	1938	19819	4099	9718	2147	6905	4078	5081	6335
LMNB2-2	2042	14	2131	163	1486	529	1058	1047	728	1317	217	2090	10	1911	4786	361	4347	748	4585	1312	2070	770	1538	1058	1012	1518
LMX1B-4	1029	8	1049	83	712	215	616	494	435	778	102	896	7	1255	12	70	3	114	4	213	17	114	0	207	5	299
MAD1L1-2	3376	22	3347	142	2215	541	1871	1060	1225	1782	274	2438	7	2893	571	147	468	245	457	502	350	270	151	467	179	729
MAD1L1-3	662	8	654	95	520	235	419	323	196	532	29	640	4	887	373	120	224	198	279	392	218	157	188	274	151	397
MAK-1	1372	12	1380	92	989	243	1016	714	644	1011	223	1512	61	1694	328	138	215	193	318	641	218	321	189	418	141	726
MAK-2	1653	16	1937	127	1173	252	1074	607	707	1069	223	1282	3	1480	388	116	335	257	244	382	195	227	176	364	130	435
MBD1-3	1308	9	1144	109	1188	374	629	600	264	970	101	1584	1	2307	514	80	204	89	119	107	442	106	237	115	68	59
MBD1-4	30279	374	27241	1384	18467	4573	15268	8912	9544	10900	3552	16566	105	21575	7504	2338	5474	4030	5977	6815	9378	2816	7396	4309	4994	6988
MBD2-1	213	3	186	21	105	44	91	56	79	168	28	133	0	237	0	2	0	9	0	9	2	6	3	7	2	20
MBD2-3	26207	314	30921	1216	21133	3967	14058	7537	6604	11112	1549	18963	46	22664	123	1113	85	1688	68	3256	3897	2556	1637	2727	1724	5887
MBD3-1	416	1	417	3	244	23	185	65	195	95	46	86	5	174	117	29	110	19	52	24	158	38	60	34	20	32
MBD3-3	5762	5	1011	33	416	43	394	107	324	175	66	144	23	398	9725	95	6301	162	3946	154	8214	88	4931	95	2773	115
MBD4-2	8	0	23	1	5	5	4	9	8	19	2	10	0	33	59	0	24	4	18	20	22	4	17	7	1	3
MBD4-3	33	2	36	2	28	7	25	21	10	21	3	32	5	39	69	8	38	4	28	8	33	13	8	11	9	12
MECP2-1	346	11	301	63	196	118	137	197	150	563	40	845	4	882	316	110	213	209	194	233	287	139	176	230	82	217

MECP2-3	4570	37	1611	174	813	556	1175	1537	687	2120	187	2442	31	3437	1616	306	1441	552	1146	880	1712	508	1207	669	659	837
MEIS1-2	226	5	208	40	94	119	129	371	114	595	16	586	6	721	28	75	16	142	13	220	563	55	423	129	212	146
MEIS1-4	4840	46	4468	323	2923	713	2775	1878	1544	2542	512	3147	57	4240	364	689	280	1073	204	2095	6288	523	5364	881	4016	1285
MEOX2-2	65	1	76	3	63	8	25	26	26	27	6	31	0	45	19	13	9	20	5	18	3	10	2	15	0	10
MEOX2-4	3234	37	3306	153	2139	310	1860	681	907	1217	215	1856	10	3246	111	382	83	519	102	1183	58	593	49	953	25	1374
MLL-5	1021	4	903	86	662	183	568	524	386	656	94	973	7	1085	1110	116	937	162	619	256	868	120	655	296	325	325
MTA1-1	119	0	78	3	87	9	40	9	67	40	14	50	7	53	477	11	301	13	365	28	151	10	120	7	77	11
MTA1-2	45	1	70	6	30	17	25	17	36	36	11	31	9	112	81	1	73	2	86	9	60	8	20	4	27	18
MTA2-1	548	5	476	40	342	58	383	97	238	263	120	371	13	496	984	27	791	49	884	123	356	46	216	64	129	98
MTA2-2	3772	20	4583	250	2977	728	2739	1900	1895	2558	455	3908	25	4155	3798	229	3567	378	4162	944	2622	390	2211	924	1627	1221
MTA3-2	1185	9	1288	23	831	114	668	217	469	343	170	581	32	710	3011	64	2446	70	2430	190	946	69	893	153	604	227
MTF2-2	3452	27	3829	142	2459	438	1896	1115	980	1756	232	2343	39	3705	6847	135	5567	198	6187	421	1010	516	525	811	499	1327
MTF2-3	152	9	167	25	95	36	77	98	43	120	21	210	9	286	1098	27	707	32	422	53	38	31	16	25	18	45
MYL4-2	4227	38	5077	267	2794	818	2176	1552	1406	2339	563	3836	10	5015	170	564	107	768	159	1809	24	837	16	1560	15	2236
NANOG-5	118	0	116	1	74	4	97	19	59	16	6	29	0	35	2	1	2	2	0	0	0	0	0	0	0	2
NCOR1-1	123	5	143	27	71	28	62	70	69	117	25	154	9	220	84	12	99	29	204	50	138	34	128	27	112	54
NCOR1-2	5187	25	5194	197	3842	721	3467	1964	2284	3092	1216	5035	25	7478	3498	185	1931	247	2068	409	1791	149	1256	215	1166	329
NCOR2-3	623	4	460	34	284	56	319	177	210	317	49	434	4	583	277	10	151	27	160	33	172	12	78	30	69	38
NCOR2-6	215	2	268	12	163	30	174	111	104	141	35	165	6	238	333	7	235	15	290	30	327	34	197	20	177	36
NEUROG1-2	1953	22	1910	137	1104	375	1181	807	660	1629	143	1868	3	1861	5	53	4	110	12	324	4	56	0	83	3	183
NEUROG1-3	17809	329	20641	1152	13319	3787	10294	7227	6230	9816	2170	13009	38	13919	293	3348	313	4039	106	7222	49	5039	93	5099	47	8436
NKX2-2-1	1931	12	2315	114	1500	266	1529	734	817	1184	203	1640	3	2176	18	182	11	271	4	420	6	124	6	268	6	322
NKX2-2-2	776	8	673	49	437	174	361	332	456	707	122	1193	1	1675	21	166	8	209	3	324	3	114	1	279	10	395
NODAL-1	5062	21	5486	405	3713	1145	3044	2208	1751	3531	547	5626	4	6048	299	890	216	1600	238	3127	48	1470	44	2477	17	3496
NODAL-3	33092	173	35282	1733	22853	5940	15991	12987	8850	19455	2446	29022	25	31955	6366	9041	3076	9138	2233	17652	933	7387	615	11107	367	15772
NSD1-2	8343	53	8335	453	5677	1222	4379	2891	3399	4199	1106	5879	32	7348	5896	519	4818	812	5685	1704	2561	677	2044	1144	1799	2044
OGT-1	1741	22	1730	151	1112	459	792	1056	606	1670	293	2519	56	3238	4587	143	3865	266	4370	628	5897	197	5312	433	4270	663
OGT-2	63	2	40	7	34	10	39	38	68	62	14	62	14	110	132	5	157	16	146	35	297	12	247	18	232	41
OLIG2-1	17	2	25	4	11	12	25	11	10	27	5	35	1	39	1	2	0	13	7	35	0	12	0	24	1	15
OLIG2-2	208	1	123	15	110	34	93	182	65	336	5	328	0	413	15	56	20	56	5	183	0	46	0	68	0	107
ONECUT1-2	1779	20	1877	72	1100	167	1148	468	681	613	167	910	19	1431	1497	155	1314	265	1442	552	8	249	22	320	15	550
ONECUT1-3	14560	64	14875	510	8718	1352	7944	2986	4290	4726	1004	6101	23	11905	11706	923	6193	1004	4842	1850	19	704	19	1208	28	1677
OTX1-2	1499	17	1128	88	861	270	695	804	440	1180	126	1649	1	3212	35	68	27	211	35	352	10	142	5	325	1	468
OTX2-5	842	25	877	87	666	327	562	717	359	1005	148	1413	8	1889	1386	175	1146	272	1439	677	5	362	10	603	7	939
PADI4-3	3539	24	3589	288	2578	710	2185	1427	1403	2083	492	3074	14	3163	148	853	96	1015	134	1937	78	1040	33	1822	28	2112
PARP1-1	24628	171	31449	1712	21349	6076	16052	10966	7452	15177	2780	22492	721	24525	33923	326	30746	658	38519	1562	15857	3022	11317	4829	10149	9122
PARP1-3	32	1	37	7	12	3	24	9	23	21	14	22	3	31	185	2	184	3	211	10	9	2	7	5	6	1
PARP11-1	583	7	480	17	380	66	318	142	205	166	60	274	3	696	47	6	30	16	28	32	39	25	36	30	30	48
PARP11-3	341	2	307	41	300	96	172	178	126	194	45	328	4	472	112	57	50	77	21	81	119	98	54	107	31	93
PARP12-3	172	2	197	12	150	28	126	76	77	134	19	134	0	255	2	2	7	7	2	24	7	21	8	19	10	21
PARP12-5	1082	15	895	52	570	134	655	359	414	634	119	767	10	957	59	52	33	90	31	192	129	121	73	156	49	193
PARP14-4	8353	60	8595	774	6655	2014	5586	4034	3762	6902	991	9463	43	9457	2037	902	1780	1436	1639	2585	6373	1129	4798	2058	3747	3433
PARP15-1	1205	10	1214	102	834	304	829	635	515	1003	172	1244	5	1528	19	120	8	273	28	429	17	214	24	353	19	460
PARP16-1	3275	17	3748	190	2598	567	2442	1061	1273	1853	357	2751	17	2745	870	526	720	741	376	1077	856	626	491	916	298	754
PARP16-3	1098	7	276	23	81	33	162	148	92	190	21	212	2	335	22	19	20	27	7	47	15	34	8	28	11	43
PARP2-1	136	5	123	12	72	27	80	59	88	75	23	98	3	98	102	7	101	15	95	44	59	20	35	28	18	18
PARP2-3	66	0	76	7	53	2	44	14	22	15	14	52	1	42	47	12	52	5	60	7	9	2	10	3	9	6
PARP3-3	3805	30	3964	279	2308	710	2344	1512	1545	2607	410	3631	243	3793	374	837	376	1792	244	2962	2275	722	2118	1579	1371	1975
PARP4-1	4749	99	5004	1388	3112	4853	2616	9234	1345	12571	421	16812	41	18335	6578	2169	4731	3722	3451	5980	5366	3297	3047	4561	1702	6230
PARP6-1	251	8	209	26	142	57	176	150	96	191	31	290	77	403	138	41	133	66	190	127	373	83	308	100	255	158
PARP6-4	26001	117	30033	1954	18880	5594	14623	11162	9016	16152	2968	23652	65	24970	9432	4533	6231	6302	3999	8696	13488	4260	10999	6362	7964	9771
PARP8-1	135	1	77	6	86	14	101	39	39	117	13	175	1	196	46	8	19	4	22	19	3	5	7	7	0	7
PARP8-2	3486	42	2962	154	1782	437	1849	895	987	1424	288	1925	20	3134	899	105	761	212	760	329	232	170	141	247	133	413
PARP9-1	362	1	69	3	18	7	51	31	31	29	17	58	1	64	4	1	1	3	1	13	83	3	34	17	14	9
PARP9-4	3	1	4	0	1	5	2	5	2	0	1	3	0	10	0	0	0	0	0	2	8	0	2	0	1	2
PAX7-4	7231	46	8076	623	6359	1391	5503	3513	2850	5069	1081	6811	2	7483	136	649	104	1086	109	1587	39	740	16	1507	13	2271
PCGF1-1	13250	53	15963	555	10059	1599	8389	3379	5377	5201	1504	8218	56	9531	4210	1037	2906	1386	1793	1862	8006	1241	5282	1485	2826	2267
PCGF2-1	525	17	429	43	251	75	233	294	191	372	54	393	8	578	330	36	353	78	469	198	460	65	368	102	226	110
PCGF3-1	6832	31	5951	184	4125	730	4392	1726	2690	2620	819	4085	38	5319	2987	284	2587	491	3262	955	3073	524	2550	711	1733	988
PCGF3-2	592	11	591	40	294	82	323	313	245	392	91	541	34	529	407	69	400	136	446	276	529	100	503	213	231	270
PCGF5-1	296	1	327	16	184	45	179	105	84	175	14	269	1	337	17	10	17	8	15	51	550	49	314	66	161	121
PCGF5-2	214	1	135	12	134	57	81	57	70	92	3	114	2	550	49	5	18	12	24	21	731	36	286	44	252	40
PCYOX1-2	15	2	9	4	14	8	21	7	50	24	10	12	21	11	49	6	36	2	31	8	143	4	57	5	21	8
PCYOX1-3	19727	87	18594	874	13526	3044	10072	6013	5768	9806	1804	20081	171	24692	38263	1387	33621	2142	37575	5476	43508	1012	50351	2282	43629	3826
PHC1-2	12592	34	16901	270	11484	1069	9880	1945	5950	2730	1731	4029	77	5282	27656	198	24499	308	33254	801	15512	936	12920	1010	9600	1546
PHC1-4	10330	55	9088	547	7088	1784	5878	3425	4270	5719	1288	7634	235	9078	10312	174	10190	393	17456	1366	2469	1939	2272	3736	1186	4450
PHC2-1	1044	28	962	70	561	189	598	444	436	598	127	729	20	988	437	80	377	147	414	323	670	121	448	175	364	281
PHC2-2	4408	22	3611	222	2714	733	2625	1516	1382	2320	484	2811	34	4122	2919	217	2343	268	2135	541	3992	258	3825	542	2401	865
PHC3-5	9930	60	8590	644	6380	1777	5455	3835	3554	5950	1102	7774	74	8946	7925	1760	5857	2581	6681	5632	17674	1600	16467	3469	10550	4985
PHC3-6	1561	13	1933	92	1398	286	1223	552	672	743	179	1060	16	1138	747	99	490	118	296	115	2781	140	1337	195	345	100
PHF19-2	552	2	382	48	248	110	290	190	183	398	87	414	4	536	270	45	240	71	157	174	189	87	176	82	78	117
PHF19-3	204	3	128	10	115	28	112	43	84	111	6	145	1	138	75	14	66	35	131	64	86	36	65	52	34	55
PHF8-2	5893	41	1717	248	654	596	868	1633	537	2034	138	2774	16	3327	1061	226	701	354	370	591	525	367	300	431	166	622
PHF8-3	20	3	24	3	13	14	12	14	17	29	5	51	0	60	41	9	15	5	18	11	4	5	6	9	5	7
POLR2A-6	1448	20	1535	112	1010	288	993	739	732	904	190	1409	27	1463	3592	104	3351	183	3424	339	2668	173	2541	300	1587	376
POLR2B-1	809	23	721	80	468	192	395	450	299	561	108	937	72	1550	2149	53	2099	138	2440	268	954	103	717	193	648	281
POLR2B-2	412	21	371	70	196	181	186	370	159	374	49	442	27	814	414	29	377	30	559	87	336	54	246	94	218	170
PRC1-2	7640	59	8699	666	6020	2025	4634	3958	2929	5231	971	7653	34	8878	33	594	55	1140	76	2606	3559	1858	2460	2993	1935	4214
PRC1-3	1018	14	1049	52	656	192	543	526	333	718	65	1122	4	1406	1973	127	1523	205	1435	390	247	178	139	331	116	511
PRDM2-4	280	3	238	38	134	41	179	96	131	237	21	165	0	316	22	11	11	40	31	66	21	33	40	49	20	127

PRDM9-1	3373	8	3470	101	2295	317	1915	531	992	680	299	1001	23	1760	1	21	8	30	6	24	10	37	0	49	2	24
PRMT1-3	244	2	208	11	124	35	159	40	91	97	34	107	20	138	754	6	747	5	1141	15	408	21	238	27	209	23
PRMT2-1	509	5	554	75	298	137	279	360	159	517	48	749	3	1028	507	134	288	169	159	322	389	108	286	153	153	230
PRMT5-1	157	1	144	15	110	28	126	56	81	151	12	138	2	206	239	8	221	12	224	29	137	48	69	35	41	59
PRMT5-2	159	0	125	1	87	4	65	8	51	8	11	16	4	18	95	1	71	0	94	8	68	6	24	6	20	4
PRMT6-1	410	5	434	32	266	60	326	192	194	292	57	282	2	407	319	33	326	62	394	160	115	51	71	81	62	102
PRMT7-2	902	22	867	89	470	206	559	389	386	608	97	867	9	941	218	50	261	143	324	236	131	127	108	156	49	228
PRMT7-3	3083	27	3150	117	2284	524	2362	943	1583	2097	492	2234	21	2981	1442	237	1235	379	1396	952	804	424	574	696	301	994
PTEN-1	79	3	79	9	55	20	51	37	47	71	24	55	20	98	35	7	16	16	36	15	320	16	116	9	83	31
PTEN-2	212	2	282	14	180	49	142	155	154	180	34	241	14	390	93	16	82	25	73	49	651	36	373	44	268	75
RAD54L-1	266	3	205	14	124	58	120	117	90	114	14	166	2	445	301	56	124	42	143	106	2	33	1	45	4	46
RAD54L-2	1516	10	1528	66	1004	119	805	375	574	558	256	857	5	1272	1627	102	1173	206	1258	417	327	167	250	442	154	380
RARB-2	8068	74	8175	450	5427	1585	4593	2803	2559	3774	809	5481	19	7473	368	1554	278	1902	269	3351	89	2276	27	3111	29	4888
RARB-3	5	0	9	0	4	3	6	2	2	2	0	2	3	7	1	0	0	0	0	0	0	0	0	0	1	0
RB1-1	770	12	784	67	420	164	572	421	359	735	154	842	24	913	427	101	395	183	564	506	906	100	691	176	571	425
RB1-2	287	7	284	24	198	84	198	259	133	461	26	563	1	962	38	12	45	29	65	49	160	23	101	29	62	49
RBBP4-1	280	2	356	19	215	38	212	77	158	141	48	236	20	228	394	8	453	18	301	46	560	24	401	39	271	50
RBBP4-3	2184	13	2594	146	1377	614	1136	1558	614	2052	118	2833	1	2975	1010	368	640	688	704	1922	406	914	329	1427	155	1683
RBBP7-1	880	17	792	98	447	267	382	542	303	698	108	580	140	921	935	9	1099	57	1600	90	810	107	689	139	598	260
RBBP7-2	54	1	41	23	50	24	57	75	33	79	13	90	4	175	394	9	286	8	344	22	65	9	57	8	23	13
RBL2-4	2831	37	3017	279	2066	613	1889	1328	1286	2100	272	2697	35	3845	1599	366	1332	607	976	1125	2566	550	2031	900	1204	1236
REST-2	1651	13	1827	81	1296	304	1053	595	572	691	133	1036	14	1673	7106	298	4379	364	3692	553	2567	296	1552	323	1156	576
RING1-1	16946	70	18935	1078	13855	3579	11321	6293	7822	9921	2358	14237	105	17244	6726	1828	5673	2731	6889	7086	11636	2175	10569	4442	9842	6941
RING1-2	2554	18	2507	295	1397	667	1336	1437	965	2718	246	3329	10	3549	534	160	455	277	433	637	1055	201	709	277	586	522
RNF168-1	132	8	92	17	65	23	84	43	47	60	17	85	9	169	74	10	62	7	73	30	80	16	50	13	35	26
RNF168-4	85	0	51	0	24	2	66	6	17	7	5	10	2	15	350	0	65	12	69	2	42	11	27	11	7	2
RNF2-5	17	0	16	2	6	5	24	7	14	22	1	21	3	34	98	2	83	5	36	5	14	2	6	3	4	1
RNF24-1	817	15	801	67	512	175	509	396	337	675	146	879	13	1396	408	110	191	157	227	219	790	119	472	198	413	321
RNF24-2	95	1	72	5	53	22	41	15	11	27	14	29	8	50	215	30	79	30	60	27	282	20	161	38	54	16
RNF25-3	3587	20	3772	100	2370	322	2071	747	1108	1318	364	2198	8	2796	2541	244	1717	442	1681	816	2617	470	2269	665	1292	1204
RNF26-1	2254	20	1619	155	1213	421	1079	1124	624	1716	158	2797	7	3525	2657	388	1640	519	1944	1098	903	440	547	698	318	1035
RNF26-2	55	0	54	5	30	3	6	0	5	8	8	5	1	14	202	8	71	12	46	6	32	2	13	11	13	9
RNF31-2	6677	28	6150	161	4973	361	4881	915	3065	1503	949	2468	25	3092	3382	364	2685	448	2726	878	1979	265	1536	601	1028	777
RNF32-1	3078	41	3340	238	2126	627	2454	1854	1698	2624	466	3105	15	3422	288	290	184	575	253	1204	110	484	55	698	59	926
RNF32-2	39	1	12	2	12	3	4	9	2	1	0	15	1	12	0	3	1	2	0	16	0	1	1	0	0	0
RNF34-4	2167	21	1870	140	1246	504	1058	1183	532	1410	195	1982	84	2379	1863	287	1442	383	1535	960	1439	502	1184	746	877	1205
RNF38-3	532	3	513	23	308	64	318	141	246	149	72	285	14	286	549	28	447	37	437	99	286	40	202	70	137	60
RNF40-1	3823	26	4206	188	2716	587	2276	1284	1333	2010	379	2543	14	3174	1846	357	987	432	997	802	892	309	527	437	358	612
RNF40-6	8348	49	7871	741	5286	1753	4607	3805	3155	5925	809	7345	28	8378	4312	2058	3230	3018	3420	5960	3831	2285	2910	3815	1671	5065
RNF8-1	169070	793	186248	16479	127950	47517	95564	70424	59581	90107	23068	129488	118	150211	16026	55955	9219	57696	6587	88143	9661	57983	3506	72461	2492	105821
RNF8-4	8	0	2	2	13	4	1	3	5	9	1	1	0	14	16	5	11	11	8	11	14	3	5	3	4	2
RPIA-1	613	16	520	50	351	116	291	272	198	419	88	581	10	758	376	45	406	84	572	304	170	84	149	135	123	247
RPIA-2	16968	58	15366	163	11220	671	7228	1345	3763	3147	838	5563	49	12721	7703	107	4631	125	3798	228	1474	114	705	90	508	276
RPRD1A-1	5851	54	5473	240	3252	1051	2213	1712	1164	2828	269	5379	38	8766	9008	185	6172	301	5298	578	2543	416	1599	525	1069	1233
RPRD1A-2	556	9	477	48	399	74	342	205	237	280	96	353	23	558	1266	31	1064	45	1021	88	461	70	356	77	309	161
RYBP-2	4267	47	4333	181	2705	605	2440	1538	1612	2149	312	3037	79	4891	2115	125	2335	255	3282	726	2856	294	2423	579	2449	1078
RYBP-3	6425	50	5082	430	3712	1158	2884	3213	1535	4595	449	6033	51	9552	4578	212	3385	302	3461	633	2834	269	1692	366	1237	641
SAP130-2	8046	73	9303	604	6082	1939	4564	4405	2340	5063	820	8556	40	10066	6444	602	5277	940	5899	2197	3366	2017	2618	2484	1802	4980
SAP130-3	264	12	223	12	143	41	224	114	122	143	33	142	2	244	62	14	62	20	70	58	42	43	30	38	17	48
SAP18-2	7768	46	8217	349	5976	1073	4668	1906	2552	3934	639	6106	36	7585	6607	437	6261	1082	7994	2282	6018	811	6638	1859	2957	2084
SAP18-3	1281	3	1566	18	859	51	771	62	294	71	173	112	4	222	1012	6	723	19	784	30	1462	34	926	66	579	74
SAP25-1	29	1	22	3	7	5	25	16	14	21	7	25	1	28	23	5	14	6	6	6	4	1	3	4	2	9
SCMH1-3	3997	19	3149	204	1968	524	1616	1105	1273	1650	349	2252	9	3283	745	151	588	311	645	620	991	342	704	494	463	743
SCMH1-4	10385	64	10430	715	7391	2231	6727	4755	4018	7627	1233	9220	41	12512	2464	646	2044	941	2014	1793	3413	1076	2597	1916	2225	2952
SENP2-3	9082	50	9113	688	6107	1770	6270	3817	4640	7001	1160	7623	27	8951	977	733	902	1410	666	2522	1998	1515	1582	2125	846	2401
SETD1A-2	119	1	83	13	39	6	43	27	23	47	7	53	2	116	323	51	172	75	130	102	151	35	40	36	25	29
SETD1A-3	3173	27	3055	184	2133	642	2031	1429	1607	2492	390	3694	15	4019	2532	580	2374	877	2413	2148	2072	875	1578	1355	1195	1880
SETD1B-1	481	11	373	207	155	430	176	1090	140	1844	54	2191	6	2023	79	322	92	540	92	1216	145	341	54	636	43	822
SETD2-2	484	0	516	12	334	50	323	162	204	269	57	245	3	300	701	58	406	36	261	85	469	44	322	62	139	48
SETD2-4	9331	29	4079	246	2322	533	2718	1565	1737	2314	445	3079	26	3778	2848	313	2491	731	1744	1076	3018	427	2031	616	1078	898
SETD7-1	254	4	171	17	159	18	180	47	120	86	14	141	6	619	33	16	28	34	45	40	601	10	402	59	197	39
SETD7-2	6142	29	6459	228	4703	746	3696	1529	2280	2464	652	3997	23	5549	1146	384	434	429	344	664	7670	369	5794	769	5183	1197
SETD8-1	1161	11	1086	82	782	171	713	400	641	816	177	1094	21	1106	577	136	450	336	609	782	2067	292	1762	400	1024	536
SETD8-2	931	3	912	15	651	110	614	219	431	327	121	317	15	374	65	28	46	41	19	21	304	50	128	49	40	25
SETDB1-1	10	0	1	0	0	1	0	2	0	2	0	0	0	4	0	0	1	1	0	0	0	1	0	0	2	2
SETDB1-2	1	2	0	0	0	0	0	0	0	2	0	0	0	1	1	0	0	0	0	0	0	0	0	0	0	0
SETDB2-1	26	1	14	3	15	9	11	18	11	30	4	32	2	70	13	4	16	7	0	14	4	13	9	4	8	11
SETDB2-3	45	1	37	3	30	12	21	27	21	22	13	43	1	104	2	4	3	4	6	10	14	7	15	10	4	21
SETMAR-2	1059	10	1312	58	867	281	788	394	409	565	113	791	9	1183	1134	269	755	508	809	911	736	452	332	594	303	1082
SFMBT1-3	1695	27	1593	128	1050	330	1084	888	642	1124	165	1519	17	1730	377	213	291	363	293	733	244	431	204	650	120	906
SFMBT1-4	39	2	51	5	17	13	20	7	21	14	1	23	0	34	9	2	4	4	0	1	5	4	4	2	1	8
SIN3A-3	41	0	47	1	24	16	51	36	40	43	8	44	5	57	48	4	65	6	86	18	71	11	52	24	38	24
SIN3B-1	962	10	831	59	590	117	690	362	452	502	154	653	72	787	172	80	183	166	152	292	257	184	187	213	115	283
SIN3B-4	3539	32	3857	226	2858	635	2541	1682	1901	2709	424	3631	38	4416	2431	386	2445	767	2699	1937	2447	702	2037	1251	1325	1495
SIRT1-3	164	3	149	17	94	44	106	85	92	146	35	182	16	224	361	35	318	42	276	74	106	45	81	36	58	54
SIRT2-2	1657	20	1630	136	1158	404	964	778	882	1319	282	1918	14	2279	399	242	361	395	265	666	1402	331	1212	538	595	587
SIRT2-3	740	12	623	72	481	133	530	365	375	564	134	638	7	903	20	89	13	206	21	446	28	215	14	282	14	366
SIRT3-2	317	5	99	4	22	13	36	25	23	47	9	51	0	75	7	6	4	5	0	10	5	3	7	6	2	6
SIRT3-4	65	0	46	1	19	1	24	29	39	35	6	60	0	36	16	6	12	2	2	3	9	8	7	0	3	6
SIRT4-1	83	1	39	1	45	2	23	28	3	6	1	29	1	163	2	3	5	2	1	2	0	6	0	7	0	4

SIRT4-3	3420	9	3138	105	1933	232	1852	542	1106	986	399	1369	9	3405	152	41	61	27	72	29	61	17	58	22	28	69
SIRT5-1	12	1	8	3	1	2	5	4	8	20	0	20	0	18	4	1	5	1	5	2	6	0	0	4	1	4
SIRT5-2	647	9	577	47	384	123	480	426	294	531	65	714	4	915	338	64	307	153	338	273	197	185	156	280	95	362
SIRT6-1	1482	15	1384	119	876	283	861	597	435	758	137	932	17	1879	315	163	271	252	281	553	292	251	208	345	135	656
SIRT6-2	63	0	60	5	32	11	60	25	34	63	7	33	0	60	32	11	20	15	21	15	7	8	8	7	8	14
SIRT7-2	74	0	4	4	1	17	7	66	5	59	2	66	1	175	11	10	2	35	9	60	6	18	7	60	0	58
SMARCAD1-1	9	0	2	0	8	0	4	0	0	2	0	0	0	6	21	0	25	0	26	1	7	2	4	1	1	4
SMARCAD1-3	154	0	148	3	96	35	73	54	62	91	13	110	1	97	215	16	180	8	93	19	52	11	68	22	18	22
SMARCAL1-1	761	5	701	63	455	71	408	274	305	369	98	685	6	824	332	81	216	126	266	387	508	139	325	248	198	246
SMARCAL1-3	481	5	470	38	276	90	264	182	201	365	36	369	4	512	97	59	44	68	58	108	100	55	68	64	35	88
SMYD2-1	14299	48	13694	308	10073	1095	7807	1960	4597	3422	1255	6405	61	8305	8971	614	7022	986	5851	2327	9041	802	7804	1617	5585	2024
SMYD2-3	1002	16	932	76	651	177	555	337	353	535	117	752	7	825	198	50	174	93	250	235	326	97	253	113	181	220
SMYD3-6	11175	76	15354	796	10357	2541	7962	4174	4300	6727	1482	10377	48	13520	10769	991	7095	1335	6264	2270	17238	2157	10778	2359	8832	4159
SMYD3-7	364	3	404	19	253	56	237	90	90	143	41	170	6	247	289	17	196	18	208	71	273	40	90	48	87	47
SOX2-3	242	3	183	20	123	47	164	152	128	250	35	250	4	403	2333	10	2283	41	3395	91	2	115	4	200	12	212
SOX6-1	31971	194	33428	2131	23055	7086	16261	11835	8067	14138	3040	24067	48	26116	3021	11441	1242	10673	939	14929	1095	9196	688	12507	371	11974
SOX6-2	2018	22	1944	122	1205	323	1208	703	731	1099	307	1659	4	2068	81	209	40	339	58	680	69	296	49	611	40	872
SSRP1-3	3377	34	3522	220	2601	810	1815	1721	1373	2800	365	3382	63	3841	17811	330	15372	507	17776	1242	8836	980	5913	1414	3631	2001
SSRP1-4	1960	18	2285	169	1261	473	1226	977	743	1662	166	2011	21	2388	3198	117	2834	129	3723	418	2060	367	1520	466	1111	932
STAG1-1	1303	8	1412	21	862	58	844	157	526	188	155	270	52	447	727	34	521	81	537	157	2570	81	2127	112	1776	186
STAG1-3	24658	136	31154	2049	18446	4612	16627	9939	9455	13506	2756	19942	109	24116	12804	3489	7017	3717	6534	6711	23382	1963	19315	3720	13749	5789
STAT3-2	372	7	298	22	227	70	219	151	178	260	73	287	28	375	736	36	623	73	699	105	939	42	817	92	585	97
STAT3-4	56547	350	59951	4801	38535	15233	29432	27410	13661	35970	6343	51669	279	58397	57782	3532	51323	5704	55901	12288	65919	2996	62914	6681	63525	13338
SUDS3-1	261	2	275	5	192	17	93	11	52	15	13	45	0	109	331	4	189	4	111	11	136	4	97	12	69	10
SUDS3-3	1183	4	1300	24	884	67	658	184	393	204	60	415	7	554	2437	24	1524	73	1341	153	1460	109	965	190	318	142
SUV39H1-2	1369	14	1219	247	628	689	410	1225	221	1421	48	1881	0	2581	264	341	151	509	181	969	28	468	51	902	8	902
SUV39H2-2	469	11	411	23	201	81	169	138	151	168	32	352	6	847	658	60	537	62	340	103	38	24	24	43	19	99
SUV420H1-2	19764	120	21386	1212	13851	2954	12208	7800	6621	11067	2416	16560	100	19952	12278	984	9748	1613	11898	3177	10057	1590	8502	2632	6604	4949
SUZ12-2	2418	29	2867	148	1737	608	1621	1186	954	2254	282	2931	46	4087	3547	161	3507	288	4069	678	2389	300	1695	546	1510	1029
SUZ12-3	830	8	876	64	553	170	557	364	406	638	127	833	25	1152	975	31	639	49	774	140	233	22	167	38	92	59
TBX3-2	678	4	400	36	325	80	268	197	173	202	49	347	10	754	10	69	8	50	9	132	1079	75	709	106	497	160
TBX3-3	66	1	74	1	48	1	41	2	33	7	5	7	9	8	7	0	0	4	1	0	305	1	163	4	164	3
TCF3-1	3115	31	2660	256	1589	766	1513	1659	855	2130	271	3308	6	3413	1056	337	886	506	920	1268	370	662	315	1120	192	1409
TET1-1	742	9	613	48	379	76	407	180	170	191	60	251	18	526	1927	121	1188	153	1200	277	357	148	190	166	90	137
TET2-1	111	2	62	4	46	8	51	24	16	33	25	68	3	62	2	8	3	3	14	12	6	5	3	9	8	7
TET2-3	17624	121	18988	1140	13053	3424	10678	8006	6893	11224	2333	15493	54	20436	3172	2090	2271	3458	2495	6772	6476	2466	4282	4254	3469	7310
TET3-1	1180	2	1132	71	663	194	599	590	432	708	154	954	67	1050	377	138	388	232	347	480	339	200	260	325	193	439
TET3-6	10507	85	11955	802	6796	2706	6496	5752	3532	10223	1063	13375	33	15712	2293	1575	1730	2855	1018	4868	1488	2330	1202	3408	694	4295
TK1-1	76	2	51	8	74	14	58	44	41	62	15	67	1	127	20	5	13	2	13	6	5	5	0	2	0	7
TK1-2	12200	107	11834	864	7447	2637	5268	4153	3322	6324	925	8696	781	19230	22278	1038	14313	1321	11853	2037	3260	1362	1714	1691	1599	2986
TP53-2	735	3	819	13	499	29	578	94	345	83	71	131	5	204	468	30	449	93	700	215	1704	78	1374	96	974	142
TP53BP1-4	1565	10	1656	141	1175	332	916	662	585	943	182	1257	10	1539	1232	84	1161	133	1187	355	981	186	646	202	474	453
TP53BP1-7	6810	56	6325	479	4570	1367	3913	2556	2740	3888	730	5144	102	6930	5976	303	5824	562	6277	1084	4525	688	3611	1223	3216	1807
TRDMT1-2	7422	93	6285	665	4387	1563	4183	4333	2264	7144	574	8027	16	11123	557	787	463	1564	493	3359	271	1644	263	2765	133	3710
TRDMT1-4	272	16	263	37	161	88	168	172	95	272	39	315	6	504	47	46	24	71	31	177	33	91	16	136	7	220
UHRF1-1	738	13	717	71	563	250	282	467	146	858	29	1364	16	2512	4030	78	2743	174	2162	525	236	357	147	593	86	813
UHRF1-2	529	3	568	32	383	120	442	238	269	419	61	616	1	741	555	40	527	102	706	240	173	145	108	133	70	196
VPS72-1	72	4	26	3	14	14	6	7	4	9	4	17	3	95	70	0	57	2	44	6	10	1	17	6	7	3
VPS72-2	572	5	450	26	305	53	315	208	193	358	92	457	5	1115	720	43	452	41	541	126	115	37	81	30	37	57
WDR5-1	124	3	149	13	73	61	102	110	72	180	12	221	3	268	120	33	66	49	54	89	57	60	27	71	21	52
WHSC1-2	973	5	1052	58	681	208	616	488	397	723	85	846	1	1181	524	106	324	186	524	413	176	164	108	247	109	298
WHSC1-3	58	1	66	0	46	14	45	23	33	44	5	43	3	101	148	5	121	4	109	16	26	11	31	12	19	18
WHSC1L1-1	1219	16	1047	84	806	376	604	856	447	1672	118	2141	19	2136	3152	214	2744	480	3636	1443	3871	461	3382	975	1542	1005
WHSC1L1-2	271	1	196	14	156	50	144	86	77	137	10	202	2	287	350	21	277	8	312	77	580	54	284	53	240	69
WNT8A-2	808	15	724	54	406	173	403	312	233	489	62	542	3	921	9	71	2	95	1	164	4	38	2	78	0	145
YAF2-2	2083	25	1880	205	1393	808	1324	1595	554	2150	172	3375	14	3763	366	224	275	233	180	585	716	403	466	529	96	270
YAF2-3	653	6	650	28	394	55	279	126	108	265	29	316	5	606	49	5	45	37	36	45	190	22	71	62	128	106
YAF2-5	16693	92	18359	1284	13101	4445	7435	6965	3788	8480	1266	13091	53	15387	6065	2443	4041	2987	3268	4877	7897	1520	6552	3795	5479	6723
YY1-2	20	2	15	1	9	2	7	10	9	0	4	17	2	12	45	3	24	1	15	1	15	3	6	3	3	0
ZNF317-2	105	1	112	4	97	34	63	36	39	90	6	114	0	170	1	4	4	3	15	14	12	8	3	12	1	16
ZNF317-3	230	4	183	15	153	43	134	83	67	141	29	206	2	275	43	6	44	17	49	43	62	12	33	51	29	48
